# Adaptive treatment allocation and selection in multi-arm clinical trials: a Bayesian perspective

**DOI:** 10.1186/s12874-022-01526-8

**Published:** 2022-02-20

**Authors:** Elja Arjas, Dario Gasbarra

**Affiliations:** 1 University of Helsinki, Helsinki, Finland; 2 University of Oslo, Oslo, Norway

**Keywords:** Superiority trial, Phase II, Phase III, Adaptive design, Likelihood principle, Posterior inference, Decision rule, Frequentist performance, Binary data, Time-to-event data, Vaccine efficacy trial

## Abstract

**Background:**

Adaptive designs offer added flexibility in the execution of clinical trials, including the possibilities of allocating more patients to the treatments that turned out more successful, and early stopping due to either declared success or futility. Commonly applied adaptive designs, such as group sequential methods, are based on the frequentist paradigm and on ideas from statistical significance testing. Interim checks during the trial will have the effect of inflating the Type 1 error rate, or, if this rate is controlled and kept fixed, lowering the power.

**Results:**

The purpose of the paper is to demonstrate the usefulness of the Bayesian approach in the design and in the actual running of randomized clinical trials during phase II and III. This approach is based on comparing the performance of the different treatment arms in terms of the respective joint posterior probabilities evaluated sequentially from the accruing outcome data, and then taking a control action if such posterior probabilities fall below a pre-specified critical threshold value. Two types of actions are considered: treatment allocation, putting on hold at least temporarily further accrual of patients to a treatment arm, and treatment selection, removing an arm from the trial permanently. The main development in the paper is in terms of binary outcomes, but extensions for handling time-to-event data, including data from vaccine trials, are also discussed. The performance of the proposed methodology is tested in extensive simulation experiments, with numerical results and graphical illustrations documented in a Supplement to the main text. As a companion to this paper, an implementation of the methods is provided in the form of a freely available R package *’barts’*.

**Conclusion:**

The proposed methods for trial design provide an attractive alternative to their frequentist counterparts.

**Supplementary Information:**

The online version contains supplementary material available at (10.1186/s12874-022-01526-8).

## Introduction

From the earliest contributions to the present day, the statistical methodology for designing and executing clinical trials has been dominated by frequentist ideas, most notably, on testing a precise hypothesis of “no effect difference” against an alternative, using a fixed sample size, and applying a pre-specified significance level to control for Type 1 error, as a means to guard against false positives in long term. An important drawback of this basic form of the standard methodology is that the design does not include the possibility of interim analyses during the trial. Particularly in exploratory studies during phase II aimed at finding effective treatments from among a number of experimental candidates it is natural look for extended designs that allow the execution of the trial to be modified based on the results from interim analyses. For example, such results could provide reasons for terminating the accrual of additional patients to some treatments for lack of efficacy or, if the opposite is true, for allocating more patients to the treatments that turned out more successful. Allowing for earlier dissemination of such findings may then also benefit the patient population at large.

These motivations have led to the development of a whole spectrum of adaptive trial designs, and of corresponding methods for the statistical analysis of such data. An authoritative presentation of group sequential methods is provided in the monograph [1]. More general reviews of adaptive clinical trial designs, from the perspective of classical inference, can be found in, e.g., Chow and Chang [2], Mahajan and Gupta [3], Chow [4], Chang and Balser [5], Pallmann et al. [6] and Atkinson and Biswas [7]. While such adaptive designs allow for greater flexibility in the running of actual trials, their assessment is usually based on selected frequentist performance measures. In the standard version, interim analyses are planned before the trial is started, and need then to be accounted for, due to the consequent multiple testing, in computing the probability of Type 1 error. Although such rigid form of planning can be relaxed when employing the so-called alpha spending functions (e.g., Pocock [8], O’Brien and Fleming [9], Demets and Lan [10]), looking into the data before reaching the pre-planned end of the trial carries a cost either in terms of an inflated probability of Type 1 error or, if that is fixed, in a reduced power of the test to detect meaningful differences between the considered treatments.

These classical approaches in the design and execution of clinical trials have been challenged from both foundational and practical perspectives. Important early contributions include, e.g., Thompson [11], Flühler et al. [12], Berry [13], Spiegelhalter et al. [14], Berger and Berry [15], Spiegelhalter et al. [16] and Thall and Simon [17]; for a brief historical account and a large number of references, see Grieve [18]. Comprehensive expositions of the topic are provided in the monographs Spiegelhalter et al. [19], Berry et al. [20] and Yuan et al. [21].

The key argument here is the change of focus: instead of guarding against false positives in a series of trials in long term, the main aim is to utilize the full information potential in the observed data from the ongoing trial itself. Then, looking into the data in interim analyses is not viewed as something incurring a cost, but rather, as providing an opportunity to act more wisely. The foundational arguments enabling this change are provided by the adoption of the likelihood principle, e.g., Berger and Wolpert [22].

In practice, this also implies a change of the inferential paradigm, from frequentist into Bayesian. In Bayesian inference, the conditional (posterior) distribution for unknown model parameters is being updated based on the available data, via updates of the corresponding likelihood. In a clinical trial, it is even possible to continuously monitor the outcome data as they are observed, and thereby utilize such data in a fully adaptive fashion during the execution of the trial. The advantages of this approach are summarized neatly in the short review paper Berry [23], in Berry [24], Lee and Chu [25], and more recently, in Yin et al. [26], Ruberg et al. [27] and Giovagnoli [28]. Importantly, the posterior probabilities provide intuitively meaningful and directly interpretable answers to questions concerning the mutual comparison of different treatments, given the available evidence, and do so without needing reference to concepts such as sampling distribution of a test statistic under given hypothetical circumstances.

Much of the recent literature on adaptive methods in clinical trials falls into two categories: adaptive randomization (AR) designs, also called response adaptive randomization (RAR), and multi-arm multi-stage (MAMS) designs. In AR, the patients are randomized to the different treatment arms sequentially, with probabilities updated from the preceding outcome data either continuously or at the times of pre-planned interim analyses. For reviews on AR designs, see Chow and Chang [2] and Robertson et al. [29]. Villar et al. [30] contains a useful review of the theoretical background, connecting the theory of the optimal design of clinical trials with that of *multi-armed bandit* problems. Of particular interest to us are papers dealing with Bayesian versions of AR, where the randomization probabilities are updated directly by applying Bayes’ rule, e.g., Trippa et al. [31], Wathen and Thall [32], Wathen and Thall [33], Viele et al. [34], Viele et al. [35] and Bassi et al. [36].

MAMS designs, on the other hand, aim at selecting the best treatments, or even the single best if there is one, of several that are tested in a multi-arm trial. This is often done indirectly by applying pre-specified stopping boundaries, to determine whether a considered treatment should be dropped. Recent contributions to such designs include Wason and Jaki [37], Wason and Trippa [38], Jacob et al. [39], Wathen and Thall [32], Yu et al. [40], Ryan et al. [33] and Ryan et al. [35].

Unfortunately, general results on optimal strategies are largely lacking and their application in practice often infeasible because of computational complexity; however, see Press [41] and Yu et al. [40]. Recently, simulation based approximations have been used for applying Bayesian decision theory in the clinical trials context, e.g., Müller et al. [42], Yuan et al. [21], Alban et al. [43] and Bassi et al. [36].

Here we consider adaptive designs mainly from the perspective of multi-arm phase II clinical trials, in which one or more experimental treatments are compared to a control. However, the same ideas can be applied, essentially without change, in confirmatory phase III trials, where usually only a single experimental treatment is compared to a control, but the planned size of the trial is larger. In both situations, treatment allocation of individual trial participants is assumed to take place according to a fixed block randomization, albeit with an important twist: The performance of each treatment arm is assessed after every measured outcome in terms of the posterior distribution of a corresponding model parameter. Different treatments arms are then compared to each other according to pre-defined criteria. If a treatment arm is found to be inferior in such a comparison to the others, it can be closed off either temporarily or permanently from further accrual.

We consider first, in The case of Bernoulli outcomes section, the simple situation in which the outcomes are binary, and they can be observed soon after the treatment has been delivered. In namerefsection:no:3 section, the approach is extended to cover situations in which either binary outcomes are measured after a fixed time lag from the treatment, or the data consist of time-to-event measurements, with the possibility of right censoring. This section includes also some notes on vaccine efficacy trials. The paper concludes with a discussion in Discussion section. A Supplement accompanied with the main text reports results from extensive simulation experiments, which follow closely the settings of two examples in Villar et al. [30] but apply the adaptive methods introduced in The case of Bernoulli outcomes section. The presentation is to a large extent comparative and expository. As a companion to this paper, we provide an implementation of the proposed method in the form of a freely available R package called *barts*, Marttila et al. [44], that facilitates the simulation of clinical trials with adaptive treatment allocation and selection.

## The case of Bernoulli outcomes

### An adaptive method for treatment allocation: rule 1

As in Villar et al. [30] and Jacob et al. [39] and numerous other papers, we consider first the ‘prototype’ example of a trial with binary outcomes and two types of treatments, one type representing a *control* or *reference* treatment indexed by 0, and *Kexperimental* treatments indexed by *k*,1≤*k*≤*K*. Motivated by a *conditional exchangeability* postulate between trial participants (with conditioning corresponding to their allocation to the different treatment arms), independent Bernoulli outcomes can in this case be assumed for all treatments, with respective response rates *θ*_0_ and *θ*_1_,*θ*_2_,…,*θ*_*K*_ considered as model parameters. We write *θ*=(*θ*_0_,*θ*_1_,…*θ*_*K*_) and use, for clarity, boldface notation ***θ***_*k*_ when the parameters are unknown and considered as random variables. Denote also, for later use, ***θ***_∨_= max{***θ***_0_,***θ***_1_,…,***θ***_*K*_}.

We index the participants in their order of recruitment to the trial by *i*,1≤*i*≤*N*_max_, where *N*_max_ is an assumed maximal size of the trial. If no such maximal size is specified, we choose *N*_max_ to be infinite. In this prototype version it is assumed that, for each *i*, the outcome *Y*_*i*_ from the treatment of patient *i* is observed soon after the treatment has been delivered. This assumption simplifies the consideration of adaptive designs, as the rule applied for deciding the treatment given to each participant can then directly account for information on such earlier outcomes. The meaning of ‘soon’ here should be understood in a relative sense to the accrual of participants to the trial. If the considered medical condition is rare in the background population, accrual will usually be slow with relatively long times between the arrivals. Then this requirement of outcome information being available when the next participant arrives may apply even if ‘soon’ is not literally true in chronological time. Extensions of this simple situation are considered in namerefsection:no:3 section.

We assume that, before starting the trial, a sequential block randomization to the treatment arms 0,1,...,*K* has been performed. We index by *n*≥1 the positions on that list, calling *nlist index*, and denote by *r*(*n*) the corresponding treatment arm. Thus, we have a fixed sequence *r*=((*r*(1),*r*(2),...,*r*(*K*+1)),(*r*(*K*+2),*r*(*K*+3),...,*r*(2(*K*+1)),...) of randomized blocks of length *K*+1, where the blocks are independent uniformly distributed random permutations of the treatment arm indexes 0,1,...,*K*.

Allocation of the participants to the different treatment arms is now assumed to follow this list, but with the possibility of skipping a treatment arm in case it has been determined to be in the *dormant* state for the considered value of *n*. This leads to a balanced design in the sense that, as long as no treatment arms have been skipped by the time of considering list index *n*, the numbers of participants allocated to different treatments can differ from each other by at most 1, and they are equal when *n* is a multiple of *K*+1.

Denote by *I*_*k*,*n*_ the binary indicator variable of arm *k* being in *active* state at list index value *n*, *n*≥0,0≤*k*≤*K*, and let *I*_*n*_=(*I*_0,*n*_,*I*_1,*n*_,...,*I*_*K*,*n*_) be the corresponding activity state vector. The values of these vectors are determined in an inductive manner to be specified later.

By inspection we find that, at the time a value *n*≥1 of the list index is considered, altogether

1 $$\begin{array}{*{20}l}  N(n) = \sum_{m=1}^{n}I_{r(m),m-1} \end{array} $$

trial participants have so far arrived and been allocated to some treatment. Clearly *N*(*n*)≤*n*. Let now the sequence {*N*^−1^(*i*);*i*≥1} be defined recursively by

2 $$\begin{array}{*{20}l} {}N^{-1}(1) &\,=\, 1;\;\\ {}N^{-1}(i) &\,=\, \min\! \left\{ n > N^{-1}(i-1): I_{r(n),n-1} = 1 \right \}, i > 1. \end{array} $$

Then *N*^−1^(*i*) is the value of the list index *n* at which participant *i* is assigned to a treatment, while *A*_*i*_=*r*(*N*^−1^(*i*)) is the index of the corresponding treatment arm. Having postulated independent Bernoulli outcomes with treatment arm specific parameters *θ*_*k*_,0≤*k*≤*K*, we then get that *Y*_*i*_ is distributed according to *Bernoulli*$\phantom {\dot {i}\!}(\theta _{r(N^{-1}(i))}).$

The distinction between active and dormant states is that no trial participants are allocated, at a value *n* of the list index, to a treatment arm *r*(*n*) if it is in the dormant state. Generally speaking, treatments whose performance in the trial has been poor, in a relative sense to the others, are more likely to be transferred into the dormant sate. However, with more data, there may later turn out to be sufficient evidence for such a trial arm to be returned back to the active state.

For *n*≥1, the activity states *I*_*n*_ will be determined in an inductive manner during the trial, and will then depend, according to criteria specified below, on the earlier treatment allocations and on the corresponding observed outcomes. The data *D*_*n*_ that have accrued from the trial when it has proceeded up to list index value *n* consist of the values of the state indicators *I*_*k*,*m*−1_, 0≤*k*≤*K*,1≤*m*≤*n*, and of treatments *A*_*i*_ and outcomes *Y*_*i*_ for *i*≤*N*(*n*).

To explain the algorithm, suppose that initially, for list index value *n*=0, all treatment arms are active so that *I*_0_=(*I*_0,0_,*I*_1,0_,...,*I*_*K*,0_)=(1,1,...,1). More generally, their activity states can be determined from the prior of ***θ***. The first participant recruited to the trial, indexed by *i*=1, is allocated to arm *r*(1) and then given the respective treatment *A*(1)=*r*(1). The outcome *Y*_1_ is then measured and included, as part, in the data *D*_1_. After this, the value of the activity vector *I*_0_ is updated into *I*_1_ as follows: If *r*(1)=*k* is an experimental treatment arm, we let *I*_*k*,1_=0 if *ℙ*_*π*_(***θ***_*k*_=***θ***_∨_|*D*_1_)<*ε*, and otherwise *I*_*k*,1_=1. Similarly, if *r*(1)=0 is the control arm, we let *I*_0,1_=0 if *ℙ*_*π*_(***θ***_0_+*δ*≥***θ***_∨_|*D*_1_)<*ε*, and otherwise *I*_0,1_=1. In a 2-arm trial obviously *ℙ*_*π*_(***θ***_0_+*δ*≥***θ***_∨_|*D*_1_)=*ℙ*_*π*_(***θ***_0_+*δ*≥***θ***_1_|*D*_1_) and *ℙ*_*π*_(***θ***_1_=***θ***_∨_|*D*_1_)=*ℙ*_*π*_(***θ***_1_≥***θ***_0_|*D*_1_).

Here the threshold values *ε*>0 and *δ*≥0 are selected design parameters of the algorithm. A smaller value of *ε* reflects then a more conservative attitude towards moving a treatment arm into the dormant state. The value of *δ* can be viewed as specifying the *minimal important difference* (MID) or *minimal clinically important difference* (MCID) in the trial; if positive, it provides some extra protection to the control arm from being moved into the dormant state.

The general step of the induction follows the same pattern: Consider a list index *n*≥1. If *r*(*n*)=*k* is an experimental treatment arm, we let *I*_*k*,*n*_=0 if *ℙ*_*π*_(***θ***_*k*_=***θ***_∨_|*D*_*n*_)<*ε*, and otherwise *I*_*k*,*n*_=1. Similarly, if *r*(*n*)=0 is the control arm, we let *I*_0,*n*_=0 if *ℙ*_*π*_(***θ***_0_+*δ*≥***θ***_∨_|*D*_*n*_)<*ε*, and otherwise *I*_0,*n*_=1. The earlier activity state vector *I*_*n*−1_ has thereby been updated to a new value *I*_*n*_. After this, the value of the list index is increased by 1, from *n* to *n*+1. Again, in a 2-arm trial, *ℙ*_*π*_(***θ***_0_+*δ*≥***θ***_∨_|*D*_*n*_)=*ℙ*_*π*_(***θ***_0_+*δ*≥***θ***_1_|*D*_*n*_) and *ℙ*_*π*_(***θ***_1_=***θ***_∨_|*D*_*n*_)=*ℙ*_*π*_(***θ***_1_≥***θ***_0_|*D*_*n*_).

A pseudocode of this algorithm, called *BARTA* (for *Bayesian adaptive rule for treatment allocation*), is provided in Section A of the Supplement.

As a byproduct, successive applications of *BARTA* give us an explicit expression for the likelihood *L*(*θ*|*D*_*n*_)=*L*_*n*_(*θ*),*n*≥1, arising from observing data *D*_*n*_. According to this rule, the likelihood expression *L*(*θ*|*D*_*n*_) is updated only at values of *n* at which *I*_*r*(*n*),*n*_=1, and is then performed by multiplying the previous value *L*(*θ*|*D*_*n*−1_) by the factor $\theta _{r(n)}^{Y_{N(n)}} \left (1- \theta _{r(n)} \right)^{1 - Y_{N(n)} }$. By repeatedly applying the chain multiplication rule for conditional probabilities, we get that

3 $$ {}\begin{aligned} L \left(\theta \vert D_{n} \right) &= \prod_{m=1}^{n}\theta_{r(m)}^{I_{r(m),m}Y_{N(m)}} \left(1- \theta_{r(m)} \right)^{I_{r(m),m}(1 - Y_{N(m)})}\\ &= \prod_{k=0}^{K} \theta_{k}^{N_{k,1} \left(n \right)} \left(1- \theta_{k} \right)^{N_{k,0} \left(n \right)}. \end{aligned}  $$

The right hand side expression is obtained by re-arranging the terms and denoting by

4 $$ {} \begin{aligned} N_{k,1}(n) &= \sum_{m=1}^{n}I_{k,m}{ 1}_{\{ Y_{N(m)}=1 \}}, \; \\ N_{k,\,0}(n) & = \sum_{m=1}^{n}I_{k,m} {1}_{\{ Y_{N(m)}=0 \}}, \; 0 \leq k \leq K, \; n \geq 1, \end{aligned}  $$

respectively, the number of successful and failed outcomes from treatment *k* when considering list index values up to *n*. Of intrinsic importance in this derivation is that, when conditioning sequentially at *n* on the data *D*_*n*_, the criteria according to which the values of the indicators *I*_*k*,*n*_ are updated to *I*_*k*,*n*+1_ do not depend on the parameter *θ*. As a consequence, these updates do not contribute to the likelihood terms that would depend on *θ*. Different formulations of this result can be found in many places, e.g., Villar et al. [30].

As a consequence, we can change the focus from the full data {*D*_*n*_,*n*≥1}, indexed according to the original list indexes used for randomization, to “condensed” data $\{D_{i}^{*}, i \geq 1 \}$ indexed according to the order in which the participants were treated. We denote by

5 $$ \begin{aligned} S_{k}(i) &= \max \left\{ N_{k,1}(n): N(n) \leq i \right\},\\ \;F_{k}(i) &= \max \left\{ N_{k,0}(n): N(n) \leq i \right\}, \; 0 \leq k \leq K, \end{aligned}  $$

respectively, the number of successful and failed outcomes from treatment *k* when considering the first *i* participants. Let

6 $$\begin{array}{*{20}l} S(i) = \sum_{k=0}^{K}S_{k}(i),\;F(i) = \sum_{k=0}^{K}F_{k}(i) \end{array} $$

be the corresponding total number of successes and of failures, across all treatment arms.

Following the usual practice in similar contexts, we assume that the unknown parameter values ***θ***_0_,***θ***_1_,…,***θ***_*K*_ have been assigned independent *Beta*-priors, with *Beta* (*θ*_*k*_|*α*_*k*_,*β*_*k*_) for treatment arm *k*, where *α*_*k*_ and *β*_*k*_ are separately chosen hyperparameters. The choice of appropriate values of these hyperparameters (e.g., Thall and Simon [17]) is always context specific, and is not discussed here further. Then, due to the well-known conjugacy property of the *Beta*-priors and the Bernoulli-type likelihood (3), the posterior $ p \left (\theta _{k} \vert D_{k,i}^{*} \right)$ for ***θ***_*k*_, corresponding to data $D_{i}^{*}$, has the form of Beta-distribution with its parameters updated directly from the data:

7 $$ {} \begin{aligned} p \left(\theta_{k} \vert D_{i,k}^{*} \right) &= \text{Beta} \left(\theta_{k} \vert \alpha_{k}+S_{k} (i), \beta_{k}+F_{k} (i) \right),~ i \geq 1,\\ k&=0, 1, \ldots,K. \end{aligned}  $$

This, together with the product form of the likelihood (3) and the assumed independence of the priors *π*, allows then for an easy computation of the joint posterior distribution for (***θ***_0_,***θ***_1_,…,***θ***_*K*_) for any *i*. The density $ p_{\pi }\!\left (\theta _{0}, \theta _{1}, \ldots, \theta _{K} \vert D_{i,k}^{*} \right) $ becomes the product of *K*+1*Beta*-densities. For example, posterior probabilities of the form *ℙ*_*π*_ (***θ***_*k*_=***θ***_∨_|*D*_*n*_), or posterior distributions for pairwise differences of the type ***θ***_*k*_−***θ***_0_ or ***θ***_*k*_−***θ***_*l*_, can be computed numerically, in practice either by numerical integration as in Jacob et al. [39], or by performing Monte Carlo sampling from this distribution; see also Zaslavsky [45]. In our numerical examples in namerefsection:no:3 section we have applied this latter possibility.

While application of *BARTA* may at least temporarily inactivate some less successful treatment arms and thereby close them off from further accrual, this closure need not be final. As long as a treatment arm is in the dormant state, and given that the priors for different treatments have been assumed to be independent, the posterior for the corresponding parameter ***θ***_*k*_ remains fixed. In contrast, with the accrual of participants to active treatment arms still continuing, the posteriors for their parameters can be expected to become less and less dispersed. As a consequence, returns from dormant to active state tend to become increasingly rare.

*BARTA* has much in common with the adaptive randomization (AR) methods considered in the literature, briefly reviewed in the Introduction. The similarity to *BARTA* is particularly close to the Bayesian versions of AR, where such adaptive updating is performed by a direct application of Bayes’ rule. The idea of employing a single initial block randomization *r*=((*r*(1),*r*(2),...,*r*(*K*+1)),(*r*(*K*+2),*r*(*K*+3),...,*r*(2(*K*+1)),...), together with considering the treatment arms to be momentarily either active or dormant, appears to be novel, however. As a result, once *r* and the operating characteristics *ε* and *δ* have been fixed, no further ’coin tossing’ is performed during the trial since each treatment allocation of a new patient is fully determined by *r* and the posterior probabilities computed from the preceding outcome data. In principle, the list *r* could be even made available in advance to all parties concerned; if this is not done, it will nevertheless be easy to check afterwards that the selected allocations were consistent with the design.

Note also that, in *BARTA*, all currently active treatment arms in a block are considered symmetrically, with exactly one patient allocated to each active treatment; after this has been done, the algorithm proceeds to considering the next permutation of the *K*+1 treatments, etc. Unless this is not regulated differently by the prior, fully balanced block randomization of all *K*+1 treatments, reminiscent to a burn-in, is applied during the early part of the trial, until there is one arm that is made dormant.

To compare, most AR-designs suggested in the literature update the randomization probabilities only at the times of a few pre-planned interim analyses, whereas in the prototype version of *BARTA*, the posterior probabilities for determining the activity states are computed after every new measured outcome. If such a continuous monitoring scheme is difficult to employ in practice, for example, for logistic reasons, it can in principle be replaced by any more appropriate non-informative stopping rule. However, the results in Viele et al. [35] suggest that, in AR designs, more frequent checks and updates are advantageous from the perspective of several different performance measures, and the same is likely to hold for *BARTA* as well.

**Thompson’s rule.** We restrict the numerical comparison of *BARTA* to a single AR design, by considering the historically oldest, classical Thompson’s rule ([11], see also, e.g., Thall et al. [46], Villar et al. [30]). In its standard version, Thompson’s rule randomizes new patients to different treatment arms *k*,0≤*k*≤*K*, directly according to the posterior probabilities *ℙ*_*π*_(***θ***_*k*_=***θ***_∨_|*D*_*n*_), updating the values of these probabilities as described above. Fractional versions of Thompson’s rule use probability weights for this purpose, based on powers (*ℙ*_*π*_(***θ***_*k*_=***θ***_∨_|*D*_*n*_))^*κ*^, with 0≤*κ*≤1, normalized into probabilities by dividing such terms by their sum over different values of *k*. Thus, for *κ*=0, the randomization is symmetric to all *K*+1 treatments, and its adaptive control mechanism becomes stronger with increasing *κ*. The results from these comparative simulation experiments are given in Sections B, C and D of the Supplement.

### An adaptive method for treatment selection: BARTS

While an open end recipe such as *BARTA* or Thompson’s algorithm may seem attractive, for example, from the perspective of drawing increasingly accurate inferences on the response parameters, practical considerations will often justify incorporation of rules for more definitive selection of some treatments and elimination of others. This is the case if the continued availability of more than one experimental treatment alternative at a later point in time is judged to be impracticable, as when entering the study into phase III. Another reason is that incorporation of such decision rules enables us to make more direct comparisons to trial designs utilizing classical hypothesis testing ideas.

With this in mind, we complement *BARTA* with an optional possibility to conclusively terminate the accrual of additional participants to the less successful treatment arms. The consequent algorithm *BARTS* (for *Bayesian adaptive rule for treatment selection*), is provided in the form of a pseudocode in Section A of the Supplement. The treatment allocation procedure is identical to that in *BARTA*, and makes a treatment arm dormant if its performance, according to pre-specified criteria, is assessed to be poor when compared to the current best. *BARTS* does the same, but will actually drop a treatment arm permanently if such judgement holds with respect to an even stricter criterion. *BARTS* can therefore be said to be an adaptation of corresponding ideas and definitions in, e.g., Thall and Wathen [47], Berry et al. [20], Xie et al. [48], Jacob et al. [39] and Wathen and Thall [32]. In the commonly adopted terminology of adaptive designs, it can be said to combine elements from different versions of AR and MAMS designs.

After every new observed outcome, the algorithm of *BARTS* determines the current state of each treatment arm, choosing between the three possible options: active, dormant, or dropped. All moves between these states are possible except that the dropped state is absorbing: once a treatment arm has been dropped, it will stay. If an arm is in dormant state, it is at least momentarily closed from further patient accrual.

Next, we explain how *BARTS* works; for the exact definition of this algorithm, see the pseudocode in the Supplement.

The posterior probability *ℙ*_*π*_ (***θ***_*k*_≥*θ*_*low*_|*D*_*n*_) for an experimental arm *k* expresses how likely it is, given the currently available data, that its response rate ***θ***_*k*_ exceeds a pre-specified level *θ*_*low*_ of *minimum required treatment response rate* (MRT), e.g., Xie et al. [48]. The first criterion in *BARTS* then says that if this probability is below a selected threshold value *ε*_1_, treatment *k* is dropped from the trial. For the control arm, the corresponding comparison is based on the posterior probability *ℙ*_*π*_ (***θ***_0_+*δ*≥*θ*_*low*_|*D*_*n*_), thereby involving an extra safety margin *δ* against accidental removal. The value of *ε*_1_ can then be said to represent an acceptable risk level of error when concluding that {***θ***_*k*_≥*θ*_*low*_} or {***θ***_0_+*δ*≥*θ*_*low*_} would not be true. This part of *BARTS* will obviously not be active if either *θ*_*low*_=0 or *ε*_1_=0.

The second criterion in *BARTS* makes a comparison of the response rate of a treatment and that of the best treatment in the trial. Both values are unknown, and the comparison is made in terms of the posterior probabilities $\P _{\pi }\left (\boldsymbol {\theta }_{k} = \max \limits _{\ell \in \mathbb {T}}\boldsymbol {\theta }_{\ell } \big \vert D_{n} \right)$ for the experimental arms and $\P _{\pi }\left (\boldsymbol {\theta }_{0}+\delta \geq \max \limits _{\ell \in \mathbb {T}}\boldsymbol {\theta }_{\ell } \big \vert D_{n}\right)$ for the control. Here $\mathbb {T} \subset \{0, 1,..., K\} $ is the set of treatment arms left in the trial at time *n*. The composition of $\mathbb {T}$ is determined in an inductive manner, starting from $\mathbb {T} = \{0, 1,..., K\} $ at *n*=1. A treatment is dropped from the trial if the corresponding posterior probability falls below the selected threshold level *ε*_2_. Thus, for small *ε*_2_, the decision to drop an experimental treatment *k* is made if, in view of the currently available data *D*_*n*_, the event $ \left \{\boldsymbol {\theta }_{k} = \max \limits _{\ell \in \mathbb {T}}\boldsymbol {\theta }_{\ell } \right \}$ is true only with probability close to 0, with *ε*_2_ representing the selected risk level. The control arm is protected even more strongly from inadvertent removal from the trial if a positive safety margin *δ* is employed. The comparison to experimental arms becomes symmetric if *δ*=0, whereas a sufficiently large value for *δ* would make it impossible to drop the control arm. This entire mechanism of eliminating treatments based on mutual comparisons is inactivated by letting *ε*_2_=0.

For later use, we denote by *n*_*k*,*last*_ the largest value of the list index for which treatment arm *k* is still left in the trial, 0≤*k*≤*K*, and by *N*_*k*,*last*_=*N*(*n*_*k*,*last*_) the index of the last patient who got treatment *k*.

The third criterion of *BARTS* copies *BARTA*: An experimental treatment arm $k \in \mathbb {T} $ is made dormant if $\P _{\pi }\left (\boldsymbol {\theta }_{k} = \max \limits _{\ell \in \mathbb {T}}\boldsymbol {\theta }_{\ell } \big \vert D_{n} \right) < \varepsilon $, and the control arm if $\P _{\pi }\left (\boldsymbol {\theta }_{0}+\delta \geq \max \limits _{\ell \in \mathbb {T}}\boldsymbol {\theta }_{\ell } \big \vert D_{n}\right) < \varepsilon $, where *ε* is a selected threshold. For this part of *BARTS* to function in a nontrivial way, we need to choose *ε*>*ε*_1_ and *ε*>*ε*_2_. If either *ε*=*ε*_1_ or *ε*=*ε*_2_, then the possibility of moving a treatment arm to the dormant state is ruled out, and if *ε*_1_=*ε*_2_=0, then *BARTS* is easily seen to collapse into the simpler rule *BARTA*. Finally, if also *ε*=0, then treatment allocation will follow directly the original block randomization, which was assumed to be symmetric between all treatment arms, and no treatments are dropped before reaching *N*_*max*_.

The selection of appropriate threshold values *δ* and *θ*_*low*_ in *BARTS* should be based on substantive contextual arguments in the trial. If a positive value for *δ* is specified, then, as already mentioned in the context of *BARTA*, this is commonly viewed as the *minimal clinically important difference* (MCID) in the trial. Employing such a positive threshold value when comparing the control to the experimental treatments reflects the idea that the design should be more conservative towards moving the control arm to the dormant state, let alone dropping it from the trial for good.

Once selected, the design parameters *ε*,*ε*_1_ and *ε*_2_ in applying *BARTS*, and then deciding to either drop the treatment or putting it into the dormant state, can be interpreted directly as upper bounds for the risk that this decision was in fact unwarranted. By *risk* is here meant the posterior probability of error, each time conditioned on the current data actually observed. Suppose, for example, that a finite value for *n*_*k*,*last*_ has been established due to $\P _{\pi }\! \left (\boldsymbol {\theta }_{k} \geq \theta _{low} \big \vert D_{n_{k,last}} \right) < \varepsilon _{1}$. Further accrual of trial participants to treatment arm *k* is then stopped after the patient indexed by *N*_*k*,*last*_ because the response rate *θ*_*k*_ from that arm is judged, with only a small probability ≤*ε*_1_, given the data, to be above the MRT level *θ*_*low*_.

If all experimental treatments have been dropped as a result of applying *BARTS*, the trial ends with a negative result, *futility*, e.g. Thall and Wathen [47]. On the other hand, if the control arm has been dropped, at least one of the experimental arms was deemed better than the control, which is a positive finding. In case more than two experimental arms were left at that time, the trial design may allow for a continued application of *BARTS*, with the goal of ultimately identifying the one with the highest response rate.

As remarked earlier, the application of *BARTS* is optional. If it is not enforced, *BARTA* is open ended and will only control the allocation of new participants to the different treatments. Then, if the trial size *N*_max_ has been specified and fixed in advance, and regardless of whether *BARTA* was previously employed or not, the posterior probabilities $\P _{\pi }\left (\boldsymbol {\theta }_{k} \geq \theta _{low} \vert D_{N_{\max }}^{*} \right),\P _{\pi } \left (\boldsymbol {\theta }_{0}+\delta \geq \boldsymbol {\theta }_{\vee } \vert D_{N_{\max }}^{*} \right)$ and $\P _{\pi } \left (\boldsymbol {\theta }_{k} = \boldsymbol {\theta }_{\vee } \vert D_{N_{\max }}^{*} \right)$ can be computed routinely after all outcome data $D_{N_{\max }}^{*}$ have been observed, to provide the final assessment of the results from the trial.

The above version of *BARTS* is intended to be used in *superiority trials*, where the goal is to select, if possible, the best treatment among those *K*+1 considered in the trial. It is another matter whether the phrase ’dropping a treatment arm’ should then be understood literally or not. For example, in a 2-arm trial, one is supposed to keep track on the posterior probabilities of the form *ℙ*_*π*_(***θ***_0_+*δ*≥***θ***_∨_|*D*_*n*_)=*ℙ*_*π*_(***θ***_0_+*δ*≥***θ***_1_|*D*_*n*_) and *ℙ*_*π*_(***θ***_1_=***θ***_∨_|*D*_*n*_)=*ℙ*_*π*_(***θ***_1_≥***θ***_0_|*D*_*n*_), and then drop an arm if either of them falls below the selected threshold value *ε*_2_. In reality, dropping the control may only mean that the experimental arm is selected for further study, perhaps in phase III. As a reviewer of this paper has pointed out, what is proposed in *BARTS* is not a *drop-the-losers* type approach, as the latter, often used in group-sequential selection, involves treatment ranking, e.g., Gerber, Gsponer, et al. [49].

This terminology is even less fitting if *BARTS* is modified to be appropriate for *non-inferiority* or *equivalence* trials, e.g. Lesaffre [50]. For example, in a 2-arm trial for the former purpose, one would be led to considering, for some *δ*>0, posterior probabilities of the form *ℙ*_*π*_(***θ***_0_−*δ*≥***θ***_1_|*D*_*n*_), and then conclude non-inferiority if such probabilities would fall below a selected *ε*_2_.

Finally, one should note that, while *BARTA* is compatible with the likelihood principle, *BARTS* has an element which violates it. This is because, in multi-arm trials with *K*>1, when considered at times *n* at which some treatment arms have already been dropped, the definition of the maximal response parameter value $ \theta _{V} = \max \limits _{\ell \in \mathbb {T}}\theta _{\ell } $ ignores those indexed in $ \{0, 1,..., K\} \setminus \mathbb {T}$. Sequential elimination of treatments, as embodied in *BARTS*, although it has an obvious practical appeal in running a clinical trial, it also renders properties such as standard Bayesian consistency inapplicable.

**A frequentist perspective**. A different perspective to the application of *BARTS* is offered by the classical frequentist theory of statistical hypothesis testing. While the main point of this paper is to argue in favor of reasoning directly based on posterior inferences, this may not be sufficient to satisfy stake holders external to the study itself, including the relevant regulatory authorities in question, which may be concerned about frequentist measures such as the overall Type 1 error rate at a pre-specified significance level (Chow and Chang [2]).

From a frequentist point of view, the posterior probabilities *ℙ*_*π*_(***θ***_0_+*δ*≥***θ***_∨_|*D*_*n*_) and *ℙ*_*π*_(***θ***_*k*_=***θ***_∨_|*D*_*n*_), via their dependence on the data *D*_*n*_, can be viewed as test statistics in respective sequential testing problems, with *BARTS* defining the stopping boundaries. In the case *K*=1, they correspond to considering two overlapping hypotheses (e.g., Richards [51]), null hypothesis *H*_0_:*θ*_1_≤*θ*_0_+*δ* and its alternative *H*_1_:*θ*_1_≥*θ*_0_. For *K*≥1, the null hypothesis becomes *H*_0_:*θ*_∨_≤*θ*_0_+*δ*, and the alternative *H*_1_:*θ*_∨_≥*θ*_0_. The posterior probabilities *ℙ*_*π*_(***θ***_0_+*δ*≥***θ***_∨_|*D*_*n*_) can then be used as test statistics in testing *H*_0_, and *ℙ*_*π*_(***θ***_∨_≥***θ***_0_|*D*_*n*_) for testing *H*_1_. Similar remarks would hold if, as remarked above, *BARTS* were modified to be used in a non-inferiority of equivalence trial.

The size of the test depends on the hypothesized “true” values of the response parameters *θ*=(*θ*_0_,*θ*_1_,…,*θ*_*K*_), on the selected threshold values *δ*,*θ*_*low*_,*ε*,*ε*_1_,*ε*_2_ and, if specified in advance, on the maximal size *N*_max_ of the trial. For clarity, we denote such a hypothesized distribution generating the data by $\mathbb {Q}$, distinct from the mixture distribution *ℙ*_*π*_ used, after being conditioned on current data, in applying *BARTA* and *BARTS*.

Frequentist measures such as true and false positive and negative rates, characterizing the performance of a test, can be computed numerically to a good approximation by performing a sufficiently large number of forward simulations from the selected $\mathbb {Q}$ and then averaging the sampled values. A more extensive consideration of such frequentist measures is here deferred to a Supplement, which contains a large number of figures and tables from simulations run under different parameter settings. Two types of experiments are considered, one concerned with a 2-arm and the other with a 4-arm trial.

**Fig. 1:**
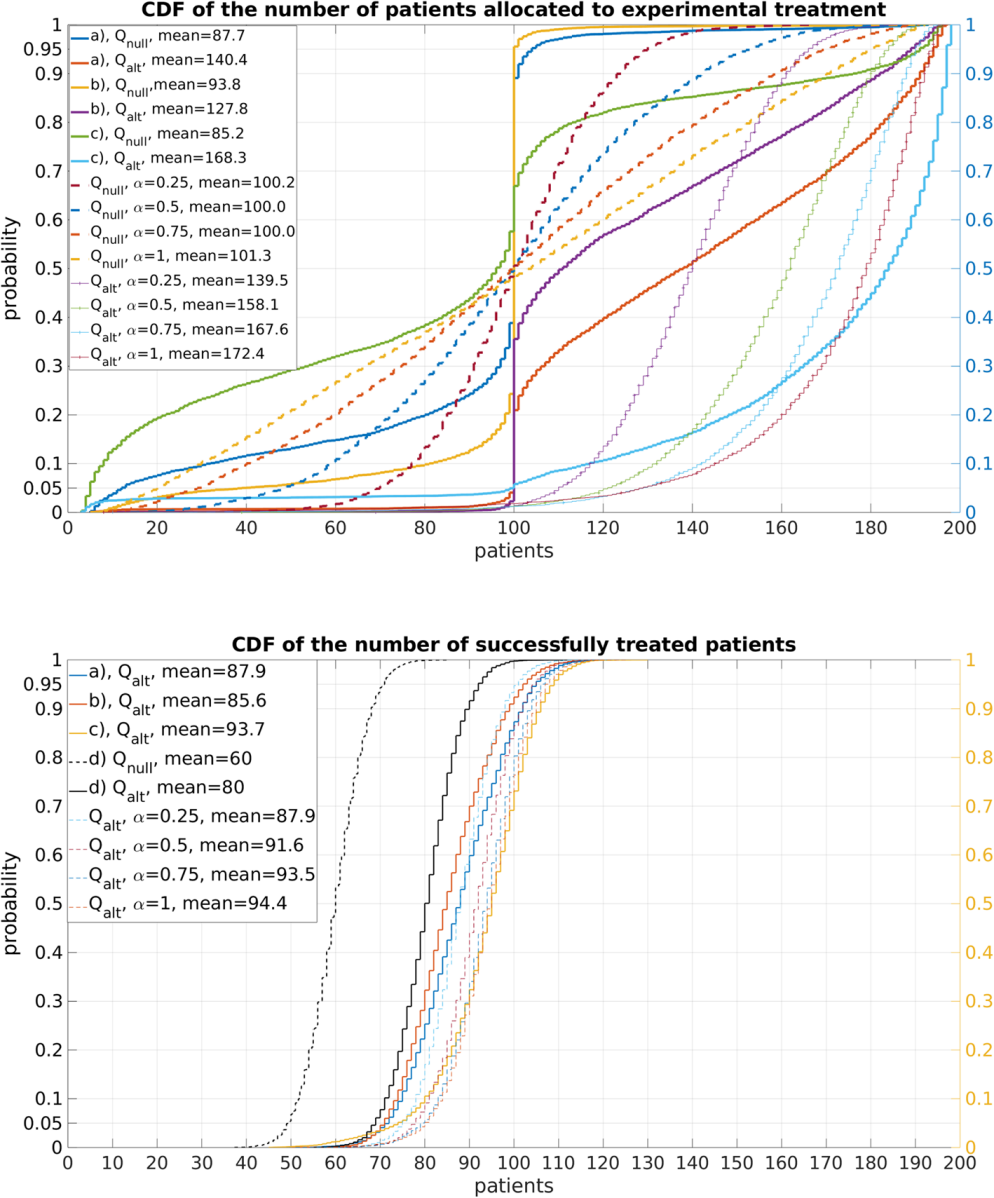
Effect of the choice of the design parameters *ε* and *δ* in *BARTA* on the number of patients allocated to the experimental treatment and on the total number of treatment successes. Cumulative distribution functions of *N*_1_(200) (top) and *S*(200) (bottom) are shown, based on 5000 simulated data sets, under $\mathbb {Q}_{null}$ with true parameter values *θ*_0_=*θ*_1_=0.3 and $\mathbb {Q}_{alt}$ with values *θ*_0_=0.3,*θ*_1_=0.5. Three combinations of the design parameters were used: (a) *ε*=0.1,*δ*=0.1, (b) *ε*=0.05,*δ*=0.1, (c) *ε*=0.2,*δ*=0.05. In addition, (d) represents a completely symmetric treatment allocation. For comparison we also plot the corresponding CDF under the alternative hypothesis obtained by using fractional Thompson’s rule with respective parameters *κ*=0.25,0.5,0.75 and 1

To give just one example of the many frequentist measures considered in the Supplement, we reproduce here Fig. S3, in Fig. 1. It shows, in the top part, the cumulative distribution functions (CDFs) of *N*_1_(200), the number of patients, of the first 200 allocated by *BARTA* to the experimental treatment, in a 2-arm trial when varying the values of the design parameters *ε* and *δ* and considering two different data generating models, $\mathbb {Q}_{null}$ and $\mathbb {Q}_{alt}$. The bottom part makes similar comparisons for *S*(200), the total number of successes from both treatments combined. Also shown are the CDFs of these variables when adaptive treatment allocation of patients was applied by using Thompson’s rule with different values of the fractional exponent *κ*.

Perhaps of most interest here is to note, from the top part of Fig. 1, how the application of *BARTA*, and particularly under $\mathbb {Q}_{null}$ in which case the treatment arms have the same true response rate, leads to often allocating exactly half of the patients to both treatment arms; this happens in trial runs during which the dormant state had not been entered even once. For Thompson’s rule, although the distribution of *N*_1_(200) under $\mathbb {Q}_{null}$ is symmetric, it has a large variance, signalling corresponding instability in treatment allocation. For additional comments on Fig. 1, see the Supplement.

Finally, one may note that such frequentist considerations are of interest essentially only at the design stage when no outcome data are yet available and a trial design needs to be selected and approved. When the trial is then run, it is natural to utilize, at each time *n*, the currently available data *D*_*n*_ and the consequent posterior probabilities such as *ℙ*_*π*_(***θ***_0_+*δ*≥***θ***_∨_|*D*_*n*_),*ℙ*_*π*_(***θ***_*k*_=***θ***_∨_|*D*_*n*_) and *ℙ*_*π*_(***θ***_*k*_≥*θ*_*low*_|*D*_*n*_). Illustrations of this can be found in Figs. S1 and S7 in the Supplement. The same holds also when declaring the final results from a trial that was carried out. In this context it may be useful to recall the well known result from general decision theory: For any prior, the smallest Bayes risk is achieved by minimizing “pointwise” the expected loss with respect to the posterior. In other words, a decision rule which is optimal locally, for each observed sample path, will be optimal also globally, on average.

## Extensions for handling delayed outcome data

Data of the kind considered in namerefsection:no:2 section, where binary outcomes are determined and observed soon after the treatment is delivered, may be rare in practical applications such as drug development. More likely, it takes some time until a response to a treatment can measured in a useful manner. For example, the status of a cancer patient could be determined one month after the treatment was given. Incorporation of such a delay into the model is not technically very difficult, but it necessitates explicit introduction of the recruitment or arrival process, in continuous time, of the patients to the trial. A somewhat different problem arises if the outcome itself is a measurement of time, such as time from treatment to relapse or to death in a cancer trial, or to infection in a vaccine efficacy study. When such information would be needed for adaptive treatment allocation, part of the data are typically right censored. Both types of extensions of the basic Bernoulli model in namerefsection:no:2 section are considered briefly below.

### Fixed delay from treatment to binary outcome

We now consider a model, where a binary outcome is systematically measured after a fixed time period has elapsed from the time at which the patient in question received the treatment. Modelling such a situation, rather obviously, requires that the model is based on a continuous time parameter.

Let, therefore, *t*>0 be a continuous time parameter, and denote by *U*_1_<*U*_2_<…<*U*_*i*_<… the arrival times of the patients to the trial, again using *i*=1,2,… to index the participants. We then assume that the treatment is always given immediately upon arrival, and that the outcome *Y*_*i*_ is measured at time *V*_*i*_=*U*_*i*_+*d*, where *d*>0 is fixed as part of the design. Let $ N(t) = \sum _{i \geq 1}^{}1_{\{ U_{i} \leq t \} },t>0, $ be the counting process of arrivals. At time *t*, outcome measurements are available from only those patients who arrived and were treated before time *t*−*d*. Therefore, the adaptive rule for assigning a treatment to a participant arriving at time *t* can utilize only the data

$$\begin{array}{*{20}l} D_{t}= \{U_{i}, A_{i},C_{i} \left(t \right),C_{i} \left(t \right) Y_{i} :i \leq N(t) \}, \end{array} $$

where the indicator $C_{i} \left (t \right) = 1_{\{U_{i}< t-d\}}$ signals that *Y*_*i*_ has been measured by time *t*.

With a minor change from (4), let

8 $$ {}\begin{aligned} N_{k,1} \left(t \right) &= \sum_{i=1}^{N \left(t \right) }C_{i} \left(t \right)1_{\{ A_{i}=k,Y_{i}=1 \} },\\ N_{k0} \left(t \right) &= \sum_{i=1}^{N \left(t \right) }C_{i} \left(t \right)1_{\{ A_{i}=k,Y_{i}=0 \} },\; 0 \leq k \leq K, 0 < t \leq T_{\max}. \end{aligned}  $$

As before, we assume that the arrival process is not informative about the model parameters, that the participants are conditionally exchangeable given their respective treatment allocations, and that the allocation rule is the same as in The case of Bernoulli outcomes section. The main distinction between the model with instantaneous response times and the present one with delayed measured outcomes is that, in the former case, once the outcome on an arriving patient becomes known, there is no additional information in the data until the next patient arrives and is treated. In the present situation, however, during such a time period some other patients, who had arrived earlier, may complete the required duration *d* from treatment to measured outcome and thereby provide new information to the data that are available. That information can then be utilized when deciding on the treatment of the next arriving patient.

By inspection we find that the basic product form of the likelihood expression (3) can be retained in this case. More concretely, the only change needed in the algorithms of *BARTA* and *BARTS* is that, instead of $L_{n}(\theta) \leftarrow L_{n-1}(\theta)\times \theta _{r(n)}^{Y_{N(n)}} \left (1- \theta _{r(n)} \right)^{1 - Y_{N(n)}},$ the inductive step for updating the likelihood becomes

9 $$\begin{array}{*{20}l} L_{n}(\theta) \leftarrow L_{n-1}(\theta) \prod_{k=0}^{K} \theta_{k}^{N_{k,1} \left(U_{N(n)}\right) - N_{k,1} \left(U_{N(n)-1} \right)}\\ \left(1- \theta_{k} \right)^{N_{k,0} \left(U_{N(n)}\right) - N_{k,0} \left(U_{N(n)-1} \right)}. \end{array} $$

### The case of time-to-event data

Time-to-event data can arise in several different ways. For example, the times from treatment to relapse or death are often used as primary endpoints in cancer trials. Below we show how *BARTA* and *BARTS* need to be modified to apply for such data.

Let *U*_*i*_ be the time of treatment and *V*_*i*_ the time of response for patient *i*, and let *X*_*i*_=*V*_*i*_−*U*_*i*_. Changing the notation slightly, we now denote by $ N(t) = \sum _{i \geq 1}^{} 1_{\{ U_{i} \leq t \}}, \; t>0, $ the process counting the arrivals to the trial. If the data are collected at time *t*, and *U*_*i*_≤*t* and *V*_*i*_>*t* hold for patient *i*, the response time *X*_*i*_ will be right censored. Observed in the data are then the times *Y*_*i*_(*t*)=[(*V*_*i*_∧*t*)−*U*_*i*_]^+^ and the indicators $ C_{i} \left (t \right) =1_{\{ V_{i} \leq t \}} =1_{\{ X_{i}=Y_{i}(t) \}}. $

Suppose now that the original response times *X*_*i*_ arising from treatment *k*, i.e., those for which *A*_*i*_=*k*, are independent and distributed according to some distribution *F*(*x*|*θ*_*k*_) with respective parameter value *θ*_*k*_>0, *k*=0,1,…,*K*. Denote the corresponding densities by *f*(*x*|*θ*_*k*_). As above, we assume that the arrival process is not informative about the model parameters, and that the participants are conditionally exchangeable given their respective treatment allocations. Then the likelihood expression corresponding to data

$$\begin{array}{*{20}l} D_{k,t}= \left\{ U_{i},A_{i}, Y_{i} \left(t \right),C_{i}\left (t \right) :i \leq N(t), A_{i}=k \right\}, \end{array} $$

collected from treatment arm *k* up to time *t*, has the familiar form

10 $$ {}\begin{aligned} L \left(\theta_{k} \vert D_{k,t} \right) = \prod_{i=1}^{N(t) } f(X_{i} \vert \theta_{k})^{C_{i} \left(t \right)1_{\{A_{i}=k\}} }(1 - F(Y_{i}\left(t \right) \vert \theta_{k}))^{(1 - C_{i} \left(t \right))1_{\{A_{i}=k\}} }. \end{aligned}  $$

Such data are in the survival analysis literature commonly referred to as data with *staggered entry*. Due to the assumed conditional independence of the response times across the different treatment arms, given the respective parameters *θ*_*k*_, the combined data

$$\begin{array}{*{20}l} D_{t}= \bigcup_{k=0}^{K} D_{k,t}= \{ U_{i},A_{i}, Y_{i} \left(t \right),C_{i} \left(t \right):i \leq N(t)\}\end{array} $$

give rise to the product form likelihood

11 $$\begin{array}{*{20}l}  L \left(\theta \vert D_{t} \right) = \prod_{k=0}^{K} L \left(\theta_{k} \vert D_{k,t} \right), \end{array} $$

where *θ*=(*θ*_0_,*θ*_1_,…,*θ*_*K*_). Upon specifying a prior for *θ*, the posterior probabilities corresponding to the data *D*_*t*_ can then be computed and utilized in *BARTA* or *BARTS*.

#### **Remarks.**

It is well known that, in Bayesian inference, *Gamma*-distributions are conjugate priors to the likelihood arising from exponentially distributed survival or duration data, with *θ*_*k*_ representing the corresponding intensity parameters. This holds also when such data are right censored, in which case the likelihood (10) corresponding to *D*_*k*,*t*_ has the Poisson form, with $\sum _{i=1}^{N \left (t \right)} C_{i} \left (t \right) 1_{\{ A_{i}=k \}} $ being the number of measured positive outcomes and $\sum _{i=1}^{N \left (t \right) }Y_{i} \left (t \right) 1_{\{ A_{i}=k \}}$ the corresponding *Total Time on Test* (TTT) statistic. Assuming independent *Gamma* (*θ*_*k*_ | *α*_*k*_,*β*_*k*_)-priors for the respective treatment arms *k*=0,1,…,*K*, the posterior for *θ*_*k*_ corresponding to data *D*_*k*,*t*_ becomes

12 $$ {}\begin{aligned} &p \left(\theta_{k} \vert D_{k,t} \right)\\ &= \text{Gamma}\biggl(\theta_{k}\; \vert \; \alpha_{k}+ \sum_{i=1}^{N \left(t \right) } C_{i} \left(t \right) 1_{\{ A_{i}=k \}},\beta_{k}+ \sum_{i=1}^{N \left(t \right) }Y_{i} \left(t \right) 1_{\{ A_{i}=k \}} \biggr), \end{aligned}  $$

and the joint posterior *p*(*θ* | *D*_*t*_) is the product distribution of these independent marginals. □

When considering the application of *BARTA* or *BARTS* in this exponential response time model, the natural target would often be to decrease, rather than increase, the value of the intensity parameter corresponding to an experimental treatment in the trial. Moreover, for measuring the degree of such potential improvements, use of hazard ratios, or relative risks, seems often more appropriate than of absolute differences. Criteria such as *ℙ*_*π*_(***θ***_*k*_≥*θ*_*low*_|*D*_*n*_)<*ε*_1_ and $\P _{\pi } \left (\boldsymbol {\theta }_{0} + \delta \geq \max \limits _{\ell \in \mathbb {T}} \boldsymbol {\theta }_{\ell } \big \vert D_{n} \right)<\varepsilon _{2} $ applied previously in *BARTS* should then be replaced by corresponding requirements of the form *ℙ*_*π*_(***θ***_*k*_≤*θ*_*high*_|*D*_*t*_) <*ε*_1_ and $\P _{\pi } \left (\rho \boldsymbol {\theta }_{0} \leq \min \limits _{\ell \in \mathbb {T}} \boldsymbol {\theta }_{\ell } \big \vert D_{t} \right)<\varepsilon _{2},$ where *ρ*<1 is a given safety margin protecting the control arm from inadvertent dropping. Writing *ρ*= exp{−*δ*} and using *η*_*k*_=− log*θ*_*k*_ as model parameters brings us back to the absolute scale, with the last inequality becoming the requirement $\boldsymbol {\eta }_{0} + \delta \geq \max \limits _{\ell \in \mathbb {T}}{\boldsymbol {\eta }_{\ell }}.$

### Notes on application to vaccine trials

An important and timely special case of time-to-event data are data coming from large scale phase III vaccine trials. When a newly developed vaccine candidate has reached the stage when it is tested in humans for efficacy, the trial participants are usually healthy individuals and the control treatment is either placebo or some existing vaccine that has been already approved for wider use. In such trials adaptive treatment allocation is less likely to be an issue, whereas it would be important to arrive at some reasonably definitive conclusion about efficacy already before reaching the planned study endpoint *N*_*max*_. For this reason, in the recent trials for testing COVID-19 candidate vaccines in humans, the design has allowed for from two to five ‘looks‘ into the data before trial completion, usually defined as times at which some pre-specified number of infections have been observed. To our knowledge, most of these trials have applied frequentist group sequential methods for testing, adjusting the targeted significance level by suitably defined spending functions. This standard practice was followed in spite of that, arguably, in trials for experimental vaccines such as the COVID-19 candidates, for which phase II had been already successfully completed, Type 1 errors could be considered less worrisome than Type 2 errors.

Entertaining the idea that such vaccine trials had been designed by using the Bayesian framework as presented in The case of time-to-event data, this task could have been accomplished by applying *BARTS* and thereby selecting suitable values for its design parameters *ρ*,*θ*_*high*_,*ε*_1_,*ε*_2_ and *N*_*max*_, letting finally *ε*=*ε*_2_ to inactivate the separately defined adaptive mechanism for treatment allocation. For example, considering the case of a single experimental vaccine, the value *ρ*=0.4 would signify the target of sixty percent decrease in the value of the intensity parameter *θ*_1_ compared to the placebo control *θ*_0_, with a corresponding reduction in the expected number of infected individuals among those vaccinated.

The trial could then be run, and it would stop with declared *success* if a posterior probability $\P _{\pi }\bigl (\rho \boldsymbol {\theta }_{0} < \boldsymbol {\theta }_{1} \big \vert D_{i}^{*}\bigr) < \varepsilon _{2}$ were obtained for some *i*≤*N*_*max*_. On the other hand, *futility* would be declared if either $\P _{\pi }\bigl (\boldsymbol {\theta }_{1} \leq \theta _{high} \big \vert D_{i}^{*}\bigr) < \varepsilon _{1}$ or $\P _{\pi }\bigl (\rho \boldsymbol {\theta }_{0} \geq \boldsymbol {\theta }_{1} \big \vert D_{i}^{*}\bigr) < \varepsilon _{2}$ were established for such *i*. In either case, the monitoring of these probabilities could in principle be done in an open book form, and not just in a few ‘looks‘ made at pre-planned check points.

A somewhat different approach to modeling and analyzing vaccine trial data can be outlined as follows. Suppose that the design is fixed by allocating, at time *t*=0, *n*_1_ individuals to the vaccine group and *n*_0_ individuals to the placebo group. Denote by 0<*T*_1,1_<*T*_1,2_<... the times at which the individuals in the former group become infected and by 0<*T*_0,1_<*T*_0,2_<... the corresponding times in the latter group. Expressed in terms of counting processes, $N_{1}(t) = \sum _{m\geq 1}{ 1}_{ \{T_{1,m} \leq t \}}$ and $N_{0}(t) = \sum _{m\geq 1}{ 1}_{ \{T_{1,m} \leq t \}}$ count the number of infections up to time *t* in these two groups. We then assume that infections occur at respective rates (*n*_1_−*N*_1_(*t*−))*λ*_1_(*t*) and (*n*_0_−*N*_0_(*t*−))*λ*_0_(*t*), where *λ*_1_(*t*) and *λ*_0_(*t*) are unknown functions of the follow-up time *t*. In practice, *n*_1_ and *n*_0_ are large, of the order 10.000 or more, while *N*_1_(*t*) and *N*_0_(*t*) can during the observation interval be at most a few hundred. Therefore, {*N*_1_(*t*);*t*≥0} and {*N*_0_(*t*);*t*≥0} can be approximated quite well by Poisson processes with respective intensities *n*_1_*λ*_1_(*t*) and *n*_0_*λ*_0_(*t*).

Suppose that these processes are (conditionally) independent given their intensities. Then the likelihood corresponding to the data *D*_*t*_={*N*_0_(*s*),*N*_1_(*s*);*s*≤*t*}, combined from both groups and up to time *t*, gets the familiar Poisson-form expression

13 $$\begin{array}{*{20}l}  {}L(\lambda_{0},\lambda_{1} \vert D_{t}) \!= \! \prod_{k=0}^{1}\ \exp \biggl\{ \!- \!\int_{0}^{t}{n_{k}\lambda_{k}(s)ds}\biggr\} \prod_{m \leq N_{k}(t)}n_{k}\lambda_{k}(T_{k,m}).\end{array} $$

Assuming that the processes {*T*_0,*m*_;*t*≥1} and {*T*_1,*m*_;*t*≥1} do not have exact ties, we now consider their superposition {0<*T*_1_<*T*_2_<...} and the corresponding counting process $N(t) = N_{0}(t) + N_{1}(t) = \sum _{m\geq 1}{ 1}_{ \{T_{m} \leq t \}}$, which then has intensity *n*_0_*λ*_0_(*t*)+*n*_1_*λ*_1_(*t*). In what follows, for the purposes of statistical inference, this superposition is decomposed back into its components. For this, we define a sequence {*δ*(*T*_*m*_);*m*≥1} of indicators, letting {*δ*(*T*_*m*_)=1} if {*N*_0_(*T*_*m*_)−*N*_0_(*T*_*m*_−)=1}. Expressed in concrete terms, the event {*δ*(*T*_*m*_)=1} occurs if the *m*^*th*^ individual in the trial who was recorded as being infected happens to belong to the placebo group, and {*δ*(*T*_*m*_)=0} if to the vaccine group. It is well known that the conditional probabilities of these events, given *λ*_0_(.),*λ*_1_(.) and {*N*(*T*_*m*_)−*N*(*T*_*m*_−)=1}, are equal respectively to *n*_0_*λ*_0_(*T*_*m*_)(*n*_0_*λ*_0_(*T*_*m*_)+*n*_1_*λ*_1_(*T*_*m*_))^−1^ and *n*_1_*λ*_1_(*T*_*m*_)(*n*_0_*λ*_0_(*T*_*m*_)+*n*_1_*λ*_1_(*T*_*m*_))^−1^.

Estimation of the function *λ*_0_(.), describing the infection pressure in the non-vaccinated population, may be possible by utilizing data sources that are external to the trial, but estimation of *λ*_1_(.) would be hard. This problem can be circumvented if we are ready to impose a proportionality assumption, according to which, although the rates at which infections occur in the vaccine and placebo groups generally vary in time, their ratio is a constant *ρ*>0. Expressed in symbols, we assume then that *λ*_1_(*t*)=*ρλ*_0_(*t*),*t*≥0. The smaller the value of *ρ*, the better protected, according to this model, the vaccinated individuals are. The value 1−*ρ* is what is commonly called *vaccine efficacy at reducing infection susceptibility*, abbreviated as *VE*_*S*_ (e.g., Halloran et al. [52]).

The postulated proportionality property appears to be reasonable if all trial participants are vaccinated approximately at the same time, in which case *t* refers to time from vaccination, and if both groups, due to randomization, can be assumed to be exposed to approximately the same infection pressure. If the trial participants have been recruited from different geographical regions with highly varying levels of infection pressure, a stratified analysis based on a common vaccine efficacy value might still be possible. However, if vaccination takes place over a longer time period, it becomes difficult to differentiate from each other the effects of infection pressure, varying in the population with calendar time, and that of individual level susceptiblity, which is likely to depend on the build-up of the immune response and thereby on the time from vaccination.

A different matter, which has received much attention recently in connection of COVID-19 vaccine trials, is the dependence of *ρ* on age, due to the immune response in the older age groups generally developing more slowly. Stratification of the analyses by using some age threshold has been applied, but the selected thresholds have varied. This is a problem for statistical analysis as long as the numbers of infected individuals in some age groups remain low.

Supposing now a common value for *ρ*, there are two alternative approaches to be selected from: Either (i) considering joint inferences on the pair (*λ*_0_(.),*ρ*), using the “full” likelihood (13) for this purpose and introducing a separate model for a description of *λ*_0_(.), or (ii) following a path well known from the context of the Cox proportional hazards model and employing a corresponding *partial likelihood* expression (e.g., Yip and Chen [53]). In a stratified analysis, the (partial) likelihood expressions would become products across the considered strata. Here we consider briefly the approach based on partial likelihood. A comparative assessment of these approaches is beyond the scope of this presentation.

By inserting the assumed form *λ*_1_(.)=*ρλ*_0_(.) of the intensity *λ*_1_(.) into (13), it can be written, after some re-arrangement and cancellation of terms, in the form

$${}\begin{aligned} L(\lambda_{0},\rho \vert D_{t}) = & \exp\biggl\{-(n_{0} + n_{1}\rho) \int_{0}^{t} \lambda_{0}(s)ds\biggr\} (n_{0} + n_{1}\rho)^{N(t)}\prod_{m \leq N(t)} \lambda_{0}(T_{m})& \\ & \times \prod_{m \leq N(t)} \biggl(\frac{n_{0} }{n_{0} + n_{1}\rho} \biggr)^{\delta(T_{m})} \biggl(\frac{n_{1} \rho}{n_{0} + n_{1}\rho}\biggr)^{1 - \delta(T_{m})}. & \end{aligned} $$

The latter product in this expression simplifies further into

14 $$ {}\begin{aligned} L_{part}(\rho \vert D_{t}) &= {\biggl(\frac{n_{0}}{n_{0} + n_{1}\rho} \biggr)^{\sum\limits_{m \leq N(t)}\delta(T_{m})}} {\biggl(\frac{n_{1} \rho}{n_{0} + n_{1}\rho }\biggr)^{\sum\limits_{m \leq N(t)}(1 - \delta(T_{m}))}}\\ &= \theta^{N_{0}(t)}(1 - \theta)^{N(t) - N_{0}(t)}, \end{aligned}  $$

where we have denoted *θ*=*n*_0_(*n*_0_+*n*_1_*ρ*)^−1^. This is the sought-after partial likelihood and, parameterized in this way, it has the familiar Binomial form. The word *partial* signifies the fact that the parts in the “full” likelihood that were omitted in the derivation of (14) also contain the unknown model parameter *ρ*. We now proceed by employing the approximation where the partial likelihood is treated as if it were the “full”. On specifying a *Beta*(. | *α*,*β*)-prior for *θ*, and using the conjugacy property of the *Beta-Binomial* distribution family, we would get the posterior *p*(*θ* | *D*_*t*_)=*Beta*(*θ* | *α*+*N*_0_(*t*),*β*+*N*(*t*)−*N*_0_(*t*)), and further the posterior for ***ρ*** by noting that *ρ*=*n*_0_(1−*θ*)/*n*_1_*θ*.

However, a *Beta*-prior may not be fully appropriate for this particular application. More naturally we could postulate, for example, the *Uniform*(0,1) prior for *ρ*. It would correspond to the assumption that infectivity in the vaccine group cannot be larger than in the placebo group, but all values of vaccine efficacy between 0 and 100 percent are a priori equally likely. This would entail for *θ* a prior density, which is no longer of *Beta*-form. With the conjugacy property lacking in this case, the posterior can nevertheless be computed easily by applying Markov Chain Monte Carlo sampling.

While adaptive treatment allocation appears to be less of an issue in vaccine trials, there will be more interest in how, and when, results from such trials could be appropriately reported. At times such as the current SARS-CoV-2 pandemic, there is much pressure towards making the results from vaccine trials available as soon as a pre-specified level of certainty can be assured. Again, consistent with the likelihood principle, all monitoring of posterior probabilities could be done in an open book form, and not just in a few ’looks’ at pre-planned check points. For example, the trial could be run, and it could stop with declared success at time *t* if the posterior probability *ℙ*_*π*_(*VE*_*S*_≥*ve*^∗^|*D*_*t*_)>1−*ε*_1_ were obtained, with *ve*^∗^ a pre-specified minimal target value and *ε*_1_ having a small value such as 0.05 or 0.01. A similar criterion could be set up for declaring futility.

**Fig. 2:**
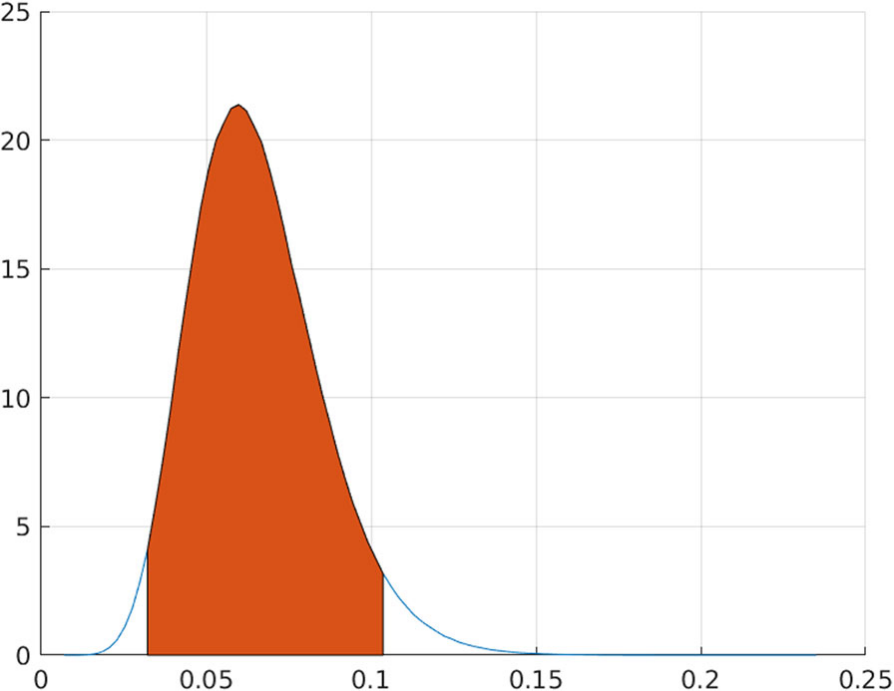
Posterior density of ***ρ*** based on Moderna, Inc. COVID-19 primary efficacy data, with posterior mode at 0.0595 and 95% HPD interval (0.030,0.105)

To give an example from a real study, Moderna, Inc. announced on November 30, 2020 (Moderna Inc. [54]) a primary efficacy analysis of their phase III COVID-19 Vaccine Candidate. The announcement, based on a randomized, 1:1 placebo-controlled study of 30.000 participants, reported 185 infections in the placebo group and 11 in the vaccine group, leading to the point estimate 11/185=0.059 of *ρ* and thereby efficacy estimate 0.941. We computed the posterior density *p*(*ρ*|*D*_*t*_) of ***ρ***, using these data *N*_0_(*t*)=185 and *N*_1_(*t*)=11 and assuming the uniform prior for ***ρ*** as described above. The result, together with the 95 percent HPDI (0.030,0.105), is shown in Fig. 2. The corresponding HPDI for *VE*_*S*_=1−*ρ* is then (0.895,0.970).

#### **Remarks.**

A practical advantage of the Poisson process approximation entertained above is that only the numbers *N*_0_(*t*) and *N*_1_(*t*) are needed for computing the posterior of ***ρ*** at time *t*. If *n*_0_ and *n*_1_ are not large enough to justify such an approximation, statistical inference based on partial likelihood is still possible, but it then necessitates monitoring of the sizes of the two risk sets. The exact times of infection are not required, but the ordering in which members of either the placebo or of the vaccine groups become infected needs to be known. As in the case of the Cox proportional hazards model, the partial likelihood expression is then somewhat more involved and the computations more slow. □

In the above approach and analysis we have assumed that the risk set sizes are reduced only due to the trial participants becoming infected. This may not be so, as there may be various other reasons why they may be lost from follow-up. If the resulting right censoring concerns a large proportion of the participants, this has to be accounted for in the analysis. It does not create a conceptually difficult problem, but it requires that the sizes of the risk sets, both in the vaccine and the placebo groups, are known at the times at which new infections are registered. The simple power form expression (14) for partial likelihood is then not valid any more, and needs to be replaced by the product

15 $$ \begin{aligned} {}L_{part}(\rho \vert D_{t}) = \prod_{T_{m}\leq t} {\biggl(\frac{R_{0,T_{m}}}{R_{0,T_{m}}+R_{1,T_{m}}\rho}\biggr)^{\delta(T_{m})}} {\biggl(\frac{R_{1,T_{m}} \rho}{R_{0,T_{m}} + R_{1,T_{m}}\rho }\biggr)^{1 - \delta(T_{m})}}, \end{aligned}  $$

where $R_{0,T_{m}}$ and $R_{1,T_{m}}$ are the sizes of the two risk sets at time *T*_*m*_. It is, in fact, a simple form of the familiar expression used for the Cox proportional hazards model, connected to the latter by the transformation *ρ*= exp{−*β*}.

Currently, several vaccines against COVID-19 have been successfully tested in placebo controlled Phase III trials and, somewhat depending on the country, have then been approved by the relevant regulatory authorities for wider use in their respective population. In addition to the original efficacy trials, there are now several studies on the population level effectiveness of COVID-19 vaccines (e.g., Dagan et al. [55], Vasileiou et al. [56]). On the other hand, in the present situation in which several vaccines that are demonstrably efficacious against both infection and the more serious forms of COVID-19 disease are available, it may be difficult to find support, for a number of different reasons, to additional large-scale placebo controlled trials for testing new candidate vaccines, cf. Krause et al. [57].

A possible alternative to such testing would be to use one or more of these existing vaccines as controls, and then make a comparative study. Such a design presents two major challenges, however. The first difficulty is demonstrated clearly by the Moderna study described briefly above: Of the approximately 15.000 individuals in the vaccine group only 11 were infected during the trial. If the candidate vaccine has at all comparable efficacy, as would naturally be desirable, the number of infected individuals in the vaccine group of a similar size, and assuming a comparable infection pressure in the study population, could not be expected to be much larger. With such small frequencies from both treatment arms in the trial, it would not be possible to arrive at a sufficiently firm conclusion concerning the desired target of *superiority* or *non-inferiority*, and this would be the case regardless of the statistical paradigm that were applied for such purpose.

To overcome this problem, it would therefore be almost mandatory to seek regulatory approval to a design in which healthy volunteers, some vaccinated by the candidate and some by an already approved vaccine, say Vaccine*, used as a control treatment, are exposed to the virus under a carefully specified protocol. The possibility of a *human challenge* design, albeit with placebo controls, was already discussed at the time when no efficacious vaccine was available (WHO [58], Eyal et al. [59], Richards [60]), and it is still considered relevant now (Eyal and Lipsitch [61]). One could anticipate that in a challenge trial, naturally depending on the level of viral exposure that would be applied, a much smaller number of participants would be needed for reaching a statistically valid conclusion on comparability. If desired, such a design could be extended to involve more than a single candidate and/or control vaccine. Note that adaptive sequential recruitment and Bayesian decision making, as exemplified by *BARTS*, would find here their natural place: It would not be necessary to fix the group sizes in advance; the trial could be run with newly recruited individuals until the desired level 1−*ε*_1_ of certainty, according to the updated posterior probabilities, has been reached.

A second issue arising in the context of such a design concerns statistical modeling and inference in a situation in which information comes from different data sources: While the design may lead to an efficacy estimate where the candidate vaccine is compared to another in routine use, this estimate cannot be readily converted to a corresponding *VE*_*S*_-estimate, where the candidate vaccine is compared to placebo. For practical consideration, this latter estimate could be the one of most interest. An approximate solution to this problem could be provided by assuming that the relative *VE*_*S*_-efficacy measures obtained from different trials, viz. an ’old’ trial for testing Vaccine* vs. placebo, and the ’new’ trial for testing the candidate vaccine vs. Vaccine*, act multiplicatively on each other, which would correspond to the structure of the Cox proportional hazards model. This would then yield a synthetic *VE*_*S*_-estimate for comparing the candidate vaccine to placebo, with a corresponding posterior derived by applying Bayesian inferential tools providing an uncertainty quantification. The relevance of this idea of combining estimates from different trials needs to be given careful scrutiny, however, and in particular since the dominant virus variant may have changed in between.

## Discussion

Clinical trials are an instrument for making informed decisions. In phase II trials, the usual goal is to make a comparative evaluation on the success rates of one or more experimental treatments to a standard or control, and in multi-arm trials, also to each other. More successful treatments among the considered alternatives, if found, can then be selected for further study, possibly in phase III.

With this as the stated goal for a trial, the conclusions should obviously be drawn as fast as possible, but not jumping ahead of the evidence provided by the acquired data. Both aspects can be accounted for by applying a suitable adaptive design, allowing for a continuous monitoring of the outcome data, and then utilizing in the execution of the trial the information that the data contain. Still, there is always the antagonism *Exploration* versus *Exploitation*: From the perspective of an individual patient in the trial, under postulated exchangeability, the optimal choice of treatment would be to receive the one with the largest current posterior mean of the success rate, as this would correspond to the highest predictive probability of treatment success. However, as demonstrated in Villar et al. [30], this *Current Belief* (CB) strategy leads to a very low probability of ultimately detecting the best treatment arm among the considered alternatives and would therefore be a poor choice when considering the overall aims of the trial.

Finding an appropriate balance between these two competing interests is a core issue in the design and execution of clinical trials, and can realistically be made only in each concrete context. For example, in trials involving medical conditions such as uncomplicated urinary infections, or acute ear infections in children, use of balanced nonadaptive 1:1 randomization to both symptomatic treatment and antibiotics groups appears fully reasonable. A very different example is provided by the famous ECMO trial on the use of the potentially life-saving technique of extracorporeal membrane oxygenation in treating newborn infants with severe respiratory failure (e.g., Bartlett et al. [62], Wolfson [63]). While statisticians advising clinical researchers have the responsibility of making available the best methods in their tool kit, there may well be overriding logistic, medical or ethical arguments which determine the final choice of the trial design. It has been even suggested that randomized clinical trials as such can present a scientific/ethical dilemma for clinical investigators, see Royall [64].

Bayesian inferential methods are naturally suited to sequential decision making over time. In the present context, this involves deciding at each time point whether to continue accrual of more participants to the trial or to stop, either temporarily or permanently, and if such accrual is continued, selecting the treatment arm to which the next arriving participant is allocated. The current joint posterior distribution of the success parameters captures then the essential information in the data that is needed for such decisions.

The posterior probabilities used for formulating the *BARTS* algorithm, when considered as functions of the accumulated data *D*_*n*_, can be viewed as test statistics in sequential tests of null hypotheses against corresponding alternatives. This link between the Bayesian and the frequentist inferential approaches makes it possible to compute, for the selected design parameters, the values of traditional performance criteria such as false positive rate and power. In the present approach, specifying a particular value for the trial size has no real theoretical bearing, and would serve mainly as an instrument for resource planning. Instead, the emphasis in the design is on making an appropriate choice of its parameters, the *ε*’s and *δ*, which control the execution of the trial, and on the direct consideration of posterior probabilities of events of the form {***θ***_*k*_=***θ***_∨_} and {***θ***_0_+*δ*≥***θ***_∨_} when monitoring outcome data from the trial.

An important difference to the methods based on classical hypothesis testing is that posterior probabilities, being conditioned on the observed data, are directly interpretable and meaningful concepts as such, without reference to their quantile value in a sampling distribution conditioned on the null. This is true regardless of whether the trial design applies adaptive treatment allocation and selection while the trial is in progress, or whether only a final posterior analysis is performed when an initially prescribed number of trial participants have been treated and their outcomes observed.

Large differences between the success parameters, if present, will often be detected early without need to wait until reaching a planned maximal trial size. On the other hand, if the joint posterior stems from an interim analysis, it forms a principled basis for predicting, in the form the consequent posterior predictive distribution, what may happen in the future if the trial is continued (e.g., Spiegelhalter et al. [14], Yin et al. [65], Hobbs et al. [66]). Note, however, that future outcomes are uncertain even in the fictitious situation in which the true values of the success parameters were known. Therefore, from the perspective of decision making, the predictive distribution involves only “more uncertainty” than the posterior, not less.

Another advantage of the direct consideration of posterior probabilities is that the joint posterior of the success parameters may contain useful empirical evidence for further study even when no firm final conclusion from the trial has been made. This is in contrast to classical hypothesis testing, where, unless the observed significance level is below the selected *α*-level so that the stated null hypothesis is rejected, the conclusion from the trial remains hanging in mid-air, without providing much guidance on whether some parts of the study would perhaps deserve further experimentation and consequent closer assessment.

The standard paradigm of null hypothesis significance testing (NHST), and particularly the version where the observed *p*-value is compared mechanistically to a selected *α*-level such as 0.05, have been criticised increasingly sharply in the recent statistical literature (e.g., Wasserstein and Lazar [67], Greenland et al. [68]). In spite of this, the corresponding strong emphasis on controlling the frequentist Type 1 error rate at a pre-specified fixed level has been largely adopted in the Bayesian clinical trials literature as well (e.g., Shi, Yin, et al. [69], Stallard et al. [70]). These error rates are conditional probabilities, evaluated from a sampling distribution under an assumed null hypothesis $\mathbb {Q}_{null}$ and in practice computed during the design stage when no actual outcome data from the trial are yet available. In contrast, in the Bayesian clinical trials methodology as outlined here, error control against false positives is performed continuously while the trial is run by applying bounds of the form $\P _{\pi }\bigl (\boldsymbol {\theta }_{0}+\delta \geq \boldsymbol {\theta }_{\vee }\big \vert D_{i}^{*}\bigr) < \varepsilon _{2}$, where the considered posterior probabilities are conditioned on the currently available trial data $D_{i}^{*}$. For this reason, in our view, calibration of Bayesian trial designs on a selected fixed frequentist Type 1 error rate (e.g., Thall et al. [46]) does not form a natural basis for comparing such designs. More generally, the role of testing a null hypothesis and the consequent emphasis on Type 1 error rate should not enjoy primacy over other relevant criteria in drawing concrete conclusions from a clinical trial (Greenland [71]). Even posterior inferences alone are not sufficient for rational decision making in such a context, and should therefore optimally be combined with appropriately selected utility functions (e.g., D.V. Lindley in Grieve et al. [72]).

If the trial is continued into phase III, this can be done in a seamless fashion by using the joint posterior of the selected treatments from phase II as the prior for phase III. In particular, if some treatment arms have been dropped during phase II, the trial can be continued into phase III as if the selected remaining treatments had been the only ones present from the very beginning. Recall, however, from the remarks made in namerefsection:no:2 section that such treatment elimination, as encoded into *BARTS*, contains a violation of the likelihood principle.

If *BARTS* is employed in phase III, and considering that phase III trials are commonly targeted at providing confirmatory evidence on the safety and efficacy of the new experimental treatment against the current standard treatment used as a control, it may be a reasonable idea to lower the threshold values *ε*_1_ and *ε*_2_ from their levels used in phase II, and thereby apply stricter criteria for final approval.

No statistical method is uniformly superior to others on all accounts. Important criticisms against the use of adaptive randomization in clinical trials have been presented, e.g., in Thall et al. [46] and Wathen and Thall [32]. In Thall et al. [46], computer simulations were used to compare adaptive patient allocation based on Thompson’s rule (Thompson [11], Villar et al. [30]) in its original and fractional forms, in a two-arm 200-patient clinical trial, to an equally randomized group sequential design. The main argument against using methods applying adaptive randomization was their potential instability, that is, there was, in the authors’ view, unacceptably large (frequentist) $\mathbb {Q}$-probability of allocating more patients to the inferior treatment arm, the opposite of the intended effect. Although these simulations were restricted to Thompson’s rule, the criticism in Thall et al. [46] was directed more generally towards applying adaptive randomization and would therefore in principle apply to our rules *BARTA* and *BARTS* as well. The results from our simulation experiments, shown in graphical form in Figs. 1, S4, S10 and S11 in the Supplement, do not support such a firm negative conclusion, however. This holds provided that the deviations from balance in the opposite directions are not weighted completely differently, and particularly so if the possibility of actually dropping a treatment arm is deferred to a somewhat later time from the beginning of the trial. A precautionary approach to the design, from a frequentist perspective, could apply a sandwich structure, starting with a symmetric burn-in, followed by an adaptive treatment allocation realized by *BARTA* or Thompson’s rule, and finally coupling in *BARTS* for actual treatment selection.

Another criticism presented in Thall et al. [46] was that, for trial data collected from a trial applying adaptive randomization, the considered tests had lower power than in the case of equal randomization, provided that the tests were calibrated to have the same Type 1 error rate. This question is discussed in subsections B.1.3 and D of the Supplement. In these experiments, adaptive treatment allocation methods based on *BARTA* designs (a) and (b), and on Thompson’s rule with fractional power *κ*=0.25, demonstrated frequentist performance quite comparable to what was observed when applying the fully symmetric block randomization design (d).

All adaptive methods favoring treatment arms with relatively more successes in the past will inevitably introduce some degree of bias in the estimation of the respective success parameters, see Bauer and Köhne [73] and Villar et al. [30]. A comprehensive review of the topic is provided in Robertson et al. [74]. We have only considered this matter briefly in the simulation experiments described in the Supplement, and instead emphasized the, in our view, more important aspect of the mutual comparison of the performance of different treatment arms in the trial. All biases in these experiments were relatively small and in the same direction, downward, and are therefore unlikely to have had a strong influence on the conclusions that were drawn.

Our main focus has been on trials with binary outcome data, where individual outcomes could be measured soon after the treatment was delivered. More complicated data situations were outlined in namerefsection:no:3. The important case of normally distributed outcome data was by-passed here; there is a large body of literature relating to it, e.g., Spiegelhalter et al. [16], Gsponer et al. [75] and Gerber, Gsponer, et al. [49]. A complication with the normal distribution is that, unless the variance is known to a good approximation already from before, there are two free parameters to be estimated for each treatment. If a suitable yardstick at the start is missing, many observations are needed before it becomes possible to separate the statistical variability of the outcome measures from true differences in the treatment effects.

In principle, the logic behind *BARTA* and *BARTS* remains valid and these rules can be applied for different types of outcome data, requiring only the ability to update the posterior distributions of the model parameters of interest when more data become available. The computation of the posteriors is naturally much less involved if the prior and the likelihood are conjugate to each other. Vague priors, or models containing more than a single parameter to be updated, will necessarily require more outcome data before adaptive actions based on *BARTA* or *BARTS* can kick in.

If such updating is not done systematically after each individual outcome is measured, for example, for logistic reasons, but less frequently in batches, *BARTA* and *BARTS* can still be used in interim analyses at the times at which the batches are completed. The same holds if updating is done at regularly spaced points in time. Such thinning of the data sequence has the effect that some of the actions that would have been otherwise implied by *BARTA* or *BARTS* are then postponed to a later time or even omitted. In designing a concrete trial, one then needs to find an appropriate balance between, on one hand, the costs saved in logistics and computation, and on the other, the resulting loss of information and the effect this may have to the quality of the inferences that can be drawn.

## Supplementary Information

**Additional file 1** Supplementary Materials.

## Data Availability

Only publicly available or simulated data were used. The R package *barts* written by Mikko Marttila generating simulated data sets and implementing the methods is freely available at https://github.com/Orion-Corporation/barts
