# Supplementary material for: Adaptive treatment allocation and selection in multi-arm clinical trials: a Bayesian perspective
Source: BMC Med Res Methodol. 2022 Feb 20;22:50. doi: 10.1186/s12874-022-01526-8 (PMC8858379; doi:10.1186/s12874-022-01526-8)
Supplement: Supplementary file 1 — Additional file 1 Supplementary Materials. [file 12874_2022_1526_MOESM1_ESM.pdf]

Supplement for the article: Adaptive treatment allocation and selection in multi-arm clinical trials: a Bayesian perspective

Elja Arjas*[1] and Dario Gasbarra[2]

[1] University of Helsinki and University of Oslo
[2] University of Helsinki

December 21, 2021

*corresponding author, elja.arjas@helsinki.fi

A Pseudocodes for BARTA and BARTS

BARTA *Adaptive rule for treatment allocation.*

---

if $\pi\left(\boldsymbol{\theta}_0+\delta \geq \boldsymbol{\theta}_{\vee}\right)<\varepsilon$ **then**
  $I_{0,0} \leftarrow 0$;
else
  $I_{0,0} \leftarrow 1$;
end
for $k \leftarrow 1$ **to** K *(experimental treatment arms)* **do**
  if $\pi\left(\boldsymbol{\theta}_k=\boldsymbol{\theta}_{\vee}\right)<\varepsilon$ **then**
    $I_{k,0} \leftarrow 0$;
  else
    $I_{k,0} \leftarrow 1$;
  end
end
$I_0 \leftarrow (I_{0,0}, I_{1,0}, ..., I_{K,0})$;
$L_0(\theta) \leftarrow 1$;
$n \leftarrow 0$;
$N(0) \leftarrow 0$;
while $N(n)<N_{\max}$ **do**
  $n \leftarrow n+1$;
  if $I_{r(n),n-1}=0$ **then**
    $N(n) \leftarrow N(n-1)$;
    $I_n \leftarrow I_{n-1}$;
    $L_n(\theta) \leftarrow L_{n-1}(\theta)$;
  else
    (in this case $I_{r(n),n-1}=1$*);*
    $N(n) \leftarrow N(n-1)+1$*;*
    $A_{N(n)} \leftarrow r(n)$*;*
    $L_n(\theta) \leftarrow L_{n-1}(\theta) \times \theta_{r(n)}^{Y_{N(n)}}\left(1-\theta_{r(n)}\right)^{1-Y_{N(n)}}$*;*
    for $k \leftarrow 1$ **to** K *(experimental treatment arms)* **do**
      if $\mathbb{P}_\pi\left(\boldsymbol{\theta}_k=\boldsymbol{\theta}_{\vee} \middle| D_n\right)<\varepsilon$ **then**
        $I_{k,n} \leftarrow 0$*;*
      else
        $I_{k,n} \leftarrow 1$*;*
      end
    end
    if $\mathbb{P}_\pi\left(\boldsymbol{\theta}_0+\delta \geq \boldsymbol{\theta}_{\vee} \middle| D_n\right)<\varepsilon$ **then**
      $I_{0,n} \leftarrow 0$*;*
    else
      $I_{0,n} \leftarrow 1$*;*
    end
  end
end

BARTS *Adaptive rule for treatment allocation and selection.*

```
if π(θ_0 + δ ≥ θ_∨) < ε then
    I_{0,0} ← 0;
else
    I_{0,0} ← 1;
end
for k ← 1 to K (experimental treatment arms) do
    if π(θ_k = θ_∨) < ε then
        I_{k,0} ← 0;
    else
        I_{k,0} ← 1;
    end
end
I_0 ← (I_{0,0}, I_{1,0}, ..., I_{K,0});
𝕋 ← {0, 1, ..., K};
N(0) ← 0;
L_0(θ) ← 1;
n ← 0 ;
while N(n) < N_max do
    n ← n + 1;
    if I_{r(n),n−1} = 0 then
        N(n) ← N(n − 1);
        I_n ← I_{n−1};
        L_n(θ) ← L_{n−1}(θ) ;
    else
        in this case (r(n) ∈ 𝕋) and (I_{r(n),n−1} = 1);
        N(n) ← N(n − 1) + 1;
        A_{N(n)} ← r(n);
        L_n(θ) ← L_{n−1}(θ) × θ_{r(n)}^{Y_{N(n)}} (1 − θ_{r(n)})^{1−Y_{N(n)}};
        for k ∈ 𝕋 \ {0} (experimental treatment arms) do
            if ℙ_π(θ_k ≥ θ_low | D_n) < ε_1 or ℙ_π(θ_k = max_{ℓ∈𝕋} θ_ℓ | D_n) < ε_2 then
                𝕋 ← 𝕋 \ {k};
                I_{k,n} ← 0 ;
                n_{k,last} ← n;
            else if ℙ_π(θ_k = max_{ℓ∈𝕋} θ_ℓ | D_n) < ε then
                I_{k,n} ← 0;
            end
            else
                I_{k,n} ← 1;
            end
        end
        if 0 ∈ 𝕋 then
            if ℙ_π(θ_0 + δ ≥ θ_low | D_n) < ε_1 or ℙ_π(θ_0 + δ ≥ max_{ℓ∈𝕋} θ_ℓ | D_n) < ε_2 then
                𝕋 ← 𝕋 \ {0};
                I_{0,n} ← 0;
                n_{0,last} ← n;
            else if ℙ_π(θ_0 + δ ≥ max_{ℓ∈𝕋} θ_ℓ | D_n) < ε then
                I_{0,n} ← 0;
            end
            else
                I_{0,n} ← 1;
            end
    end
end
```

B Illustrations of the methods by using simulation experiments

Here we illustrate the application of the methods for treatment allocation (*BARTA*) and for treatment selection (*BARTS*) by performing a number of simulation experiments from hypothesized probability distributions $\mathbb{Q}$. For this, we consider different choices for the "true" parameter values $\theta = (\theta_0, \theta_1, \ldots, \theta_K)$, varying also the values of the threshold parameters δ, ε and ε_2, and of the maximal trial size $N_{\max}$. For simplicity, and since we do not aim at modeling any contextual real data, we let $\theta_{low} = 0$ and $\varepsilon_1 = 0$ in all simulations.

Before entering the more detailed discussion of the simulation experiments, we consider briefly the choice of the prior distribution for the parameter $\boldsymbol{\theta} = (\boldsymbol{\theta}_0, \boldsymbol{\theta}_1, \ldots, \boldsymbol{\theta}_K)$. In these experiments we are using systematically independent *Uniform*(0,1)-priors for all coordinates $\boldsymbol{\theta}_k$, corresponding to the hyperparameter values $\alpha_k = \beta_k = 1$ of the *Beta*-distributions. This choice is not intended as a practical guideline, nor to be representative of choices that would be commonly made in real data situations. Instead, it is thought to be appropriate to be used as an illustration of the workings of *BARTA* and *BARTS*, as all essential information for running the trial then comes from the registered outcome data from the trial itself. Particularly on the treatment used as the control arm, there is usually a fair amount of background information from earlier experiments for specifying a more informative prior; for the relevant literature on this topic see, e.g., Spiegelhalter *et al.* [5], Thall & Simon [8], Spiegelhalter *et al.* [4] and Neuenschwander *et al.* [3].

A pair (α_k, β_k) of hyperparameters of the *Beta*-distribution is commonly thought to represent prior information equivalent to α_k successes and β_k failures from a treatment arm before initiating the trial. If the selected values α_k and β_k for some particular treatment arm k are such that their sum $\alpha_k + \beta_k$ is larger than that of the others, say $\alpha_l + \beta_l$, it may be a good idea to postpone the application of *BARTA* on arm k from the start of the trial, and use it to allocate the first participants to those other arms l until the sum $\alpha_l + \beta_l + S_l(i) + F_l(i)$ reaches the level of $\alpha_k + \beta_k$. Intuitively speaking, treatments are then compared to each other only after the joint posterior is based in the same number of (pseudo)observations from all arms.

Remark. Postulating prior independence of the different treatment effects, although very convenient from practical and computational perspectives, is unlikely to correspond exactly to honest initial beliefs held at the time a trial is designed. For example, in most trials involving binary outcomes, it would be natural to assume that $\boldsymbol{\theta}_k$ corresponding to an experimental treatment would not be widely different from $\boldsymbol{\theta}_0$ corresponding to the control. In a more elaborate modeling, such dependence could perhaps be accounted for by employing a suitable copula model, e.g., Meester & Mackay [2]. The prior dependence is then inherited by the posterior; however, its influence on the conclusions that can be drawn from the trial is likely to diminish considerably as more empirical outcome data become available.

B.1 Simulation studies with a 2-arm trial: Experiment 1

Our first simulation experiment mimics the setting of the two-arm trial described in Villar *et al.* [9], Section 5.1. In this comparison of a single experimental treatment to a control, the hypothesis $H_0 : \theta_1 \leq \theta_0$ was tested against the alternative $H_1 : \theta_1 > \theta_0$ by using Fisher's exact test at the significance level of $\alpha = 0.05$ for Type 1 error. Two alternative parameter settings

were considered in the simulations leading to the numerical results shown in Table 5 of Villar *et al.* [9], with Type 1 error rate computed at parameter values $\theta_0 = \theta_1 = 0.3$, henceforth denoted by $\mathbb{Q}_{null}$, and the power of rejecting H_0 computed at $\theta_0 = 0.3, \theta_1 = 0.5$, denoted by $\mathbb{Q}_{alt}$. The simulations and the tests were based on fixed trial size $N_{\max} = 148$.

In the present approach, instead of first collecting all planned outcome data and then performing a test at a given level of significance, the trial would be run in an adaptive manner, continuously updating the posterior probabilities specified in *BARTA* and/or *BARTS*, and then proceeding in an inductive manner according to these rules.

B.1.1 Monitoring the operation of *BARTA* in Experiment 1

We considered three different settings of the design parameters for *BARTA*: (a) $\delta = 0.1, \varepsilon = 0.1$, (b) $\delta = 0.1, \varepsilon = 0.05$, and (c) $\delta = 0.05, \varepsilon = 0.2$. Note that larger values for δ and smaller values for ε correspond to a higher degree of conservatism towards moving a treatment arm from active to dormant state, and conversely. The choice (b) is therefore more conservative than (a), while (c) is more liberal.

As an illustration of the workings of *BARTA*, we performed an experiment emulating a real trial with maximal size $N_{\max} = 500$, using a single realization generated from $\mathbb{Q}_{alt}$ and applying *BARTA* for treatment allocation with thresholds (a). For this, we considered the values of the list index n at which a new patient was allocated to either of the two treatments, i.e., $N(n) - N(n-1) = 1$, thereby skipping an index if it corresponds to an arm in the dormant state. For such values of n and until $N(n) = 500$, we monitored in Figure S1 the development of the posterior probabilities $\mathbb{P}_\pi(\boldsymbol{\theta}_0 + \delta \geq \boldsymbol{\theta}_\vee | D_n) = \mathbb{P}_\pi(\boldsymbol{\theta}_0 + \delta \geq \boldsymbol{\theta}_1 | D_n)$ and $\mathbb{P}_\pi(\boldsymbol{\theta}_1 = \boldsymbol{\theta}_\vee | D_n) = \mathbb{P}_\pi(\boldsymbol{\theta}_1 \geq \boldsymbol{\theta}_0 | D_n)$, of the posterior expectations $\mathbb{E}_\pi(\boldsymbol{\theta}_0 | D_n)$ and $\mathbb{E}_\pi(\boldsymbol{\theta}_1 | D_n)$, and of the activity indicators $I_{0,n}$ and $I_{1,n}$. Note that these functions depend only on the corresponding "condensed" simulated data $\{D_i^*, 1 \leq i \leq 500\}$.

According to *BARTA* design (a), the control arm is in dormant state for patient i if $\mathbb{P}_\pi(\boldsymbol{\theta}_0 + 0.1 \geq \boldsymbol{\theta}_1 | D_i^*) < 0.1$. The values of i for which this was the case in the considered simulation are shown in Figure S1 in grey color. For such i, no new patients were allocated to the control treatment, and therefore the corresponding cumulative sum of activity indicators $I_{0,n}$ and the posterior expectation $\mathbb{E}_\pi(\boldsymbol{\theta}_0 | D_i^*)$ remained constant. In contrast, the experimental arm was active during the entire follow-up due to all posterior probabilities $\mathbb{P}_\pi(\boldsymbol{\theta}_1 \geq \boldsymbol{\theta}_0 | D_i^*)$ staying above the threshold $\varepsilon = 0.1$. Had also *BARTS* been applied, say, with threshold values $\varepsilon_1 = 0$ and $\varepsilon_2 = 0.05$, the control arm would have been dropped from the trial at the first i for which $\mathbb{P}_\pi(\boldsymbol{\theta}_0 + 0.1 \geq \boldsymbol{\theta}_1 | D_i^*) < 0.05$. In the considered simulation this happened at $i = 365$. In Figure S1 this is indicated in dark grey.

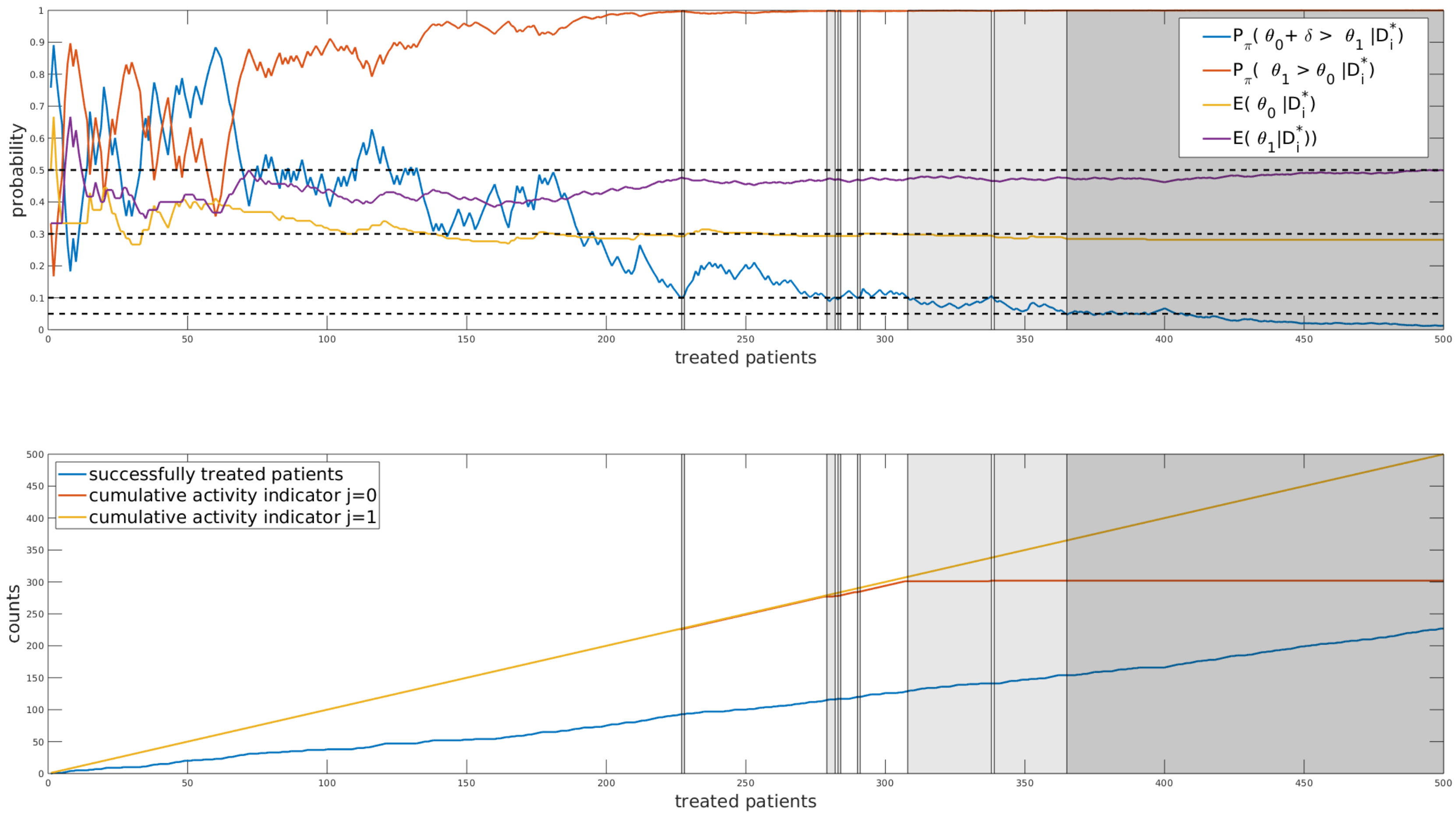

Figure S1: An example of monitoring the execution of a 2-arm trial of size $N_{\max} = 500$, simulated from $\mathbb{Q}_{alt}$ with parameter values $\theta_0 = 0.3$ and $\theta_1 = 0.5$. *BARTA* with design parameter values $\varepsilon = 0.1$ and $\delta = 0.1$ was applied for treatment allocation, with the control arm in dormant state indicated in grey color. The effect of also involving *BARTS* for treatment selection, with $\varepsilon_1 = 0$ and $\varepsilon_2 = 0.05$, is indicated in darker grey. Top: Time-evolution of the posterior probabilities $\mathbb{P}_\pi(\boldsymbol{\theta}_0 + \delta \geq \boldsymbol{\theta}_\vee | D_i^*) = \mathbb{P}_\pi(\boldsymbol{\theta}_0 + \delta \geq \boldsymbol{\theta}_1 | D_i^*)$ and $\mathbb{P}_\pi(\theta_1 = \boldsymbol{\theta}_\vee | D_i^*) = \mathbb{P}_\pi(\theta_1 > \boldsymbol{\theta}_0 | D_i^*)$, and of the posterior expectations $\mathbb{E}_\pi(\boldsymbol{\theta}_j | D_i^*), j = 0, 1; 1 \leq i \leq 500$. Bottom: Cumulative sums of activity indicators of the treatment arms and the cumulative number of treatment successes. For more details, see text.

B.1.2 Effect of the design parameters on treatment allocation

Next, we study the effect of the choice of the design parameters ε and δ in *BARTA* on some frequentist type key characteristics of a trial. Figure S2 illustrates this effect for the joint distribution of the activity indicators I_0 and I_1, considered as a function of the number of treated patients. Empirical probabilities are shown, based on 5000 simulated trials of size $N_{\max} = 500$, under $\mathbb{Q}_{null}$ with true parameter values $\theta_0 = \theta_1 = 0.3$ (left), and under $\mathbb{Q}_{alt}$ with $\theta_0 = 0.3, \theta_1 = 0.5$ (right).

For $\mathbb{Q}_{alt}$, $\theta_0 + \delta = 0.3 + 0.1 < 0.5 = \theta_1$, and therefore the posterior probabilities $\mathbb{P}_\pi(\boldsymbol{\theta}_0 + \delta \geq \boldsymbol{\theta}_1 \big| D_i^*)$ tend to be small, at least for larger values of i. When they are below the threshold ε, compliance with *BARTA* forces the control arm to be dormant. We can see this happening in Figure S2 on the right, where the $\mathbb{Q}_{alt}$ probability of $\{I_0 = 0, I_1 = 1\}$ clearly dominates that of $\{I_0 = 1, I_1 = 0\}$. The effect is strongest in the liberal parameter setting (c), and weakest but still quite strong in the conservative alternative (b). In contrast, under $\mathbb{Q}_{null}$, with $\theta_0 = \theta_1$, the configuration $\{I_0 = 1, I_1 = 1\}$ remains the most likely alternative for all considered values of i, with the strongest tendency to do so in the conservative design (b) and the weakest in the liberal (c). A third aspect to be noted on the left of Figure S2 is that the configuration $\{I_0 = 1, I_1 = 0\}$ was always much more likely than $\{I_0 = 0, I_1 = 1\}$, due to the control arm being protected by the positive safety margin δ.

Finally, Figure S2 shows the expectations of $\mathbb{E}_\pi\big(\boldsymbol{\theta}_k|D_i^*\big)$ and $\mathbb{E}_\pi\big(\boldsymbol{\theta}_k|D_i^*\big)$, $(1 \leq i \leq 500, k = 0, 1)$, computed from these simulations under $\mathbb{Q}_{null}$ and $\mathbb{Q}_{alt}$. For small i all these values are close to 0.5, originating from the *Uniform*$(0, 1)$-priors assumed in all simulations. With more data, the curves stabilize close to the true parameter values, but exhibit then a small negative bias. This is an aspect shared by all adaptive methods favoring in treatment allocation arms with relatively more successes in the past, see e.g. Villar *et al.* [9]. Given that the main goal of each on-going trial is the mutual comparison of the different treatments involved, and that this assessment is here made with respect to the joint posterior based on the current trial data, the frequentist property of a small bias in the estimation of the individual treatment success parameters, in the same direction, does not seem very crucial.

A complementary point of view is presented in Figure S3 showing the cumulative distribution functions (CDFs) of $N_1(200)$, the number of patients out of the first 200 allocated by *BARTA* to the experimental treatment, and of $S(200)$, the total number of successes from both treatments combined. Corresponding results from considering the first 100 and 500 patients are shown in Figures S10 and S11 included in part C of this Supplement.

The CDFs in these figures are based on simulated data sets from $\mathbb{Q}_{null}$ and $\mathbb{Q}_{alt}$ by using the same parameter settings (a), (b) and (c) of *BARTA* as in Figure S2. In addition, the corresponding CDFs are shown in design (d), in which case the adaptive treatment allocation property of *BARTA* was inactivated by applying threshold value $\varepsilon = 0$, thereby leading to a completely symmetric block randomization. Finally, for a comparison, also shown are the CDFs of these variables when adaptive treatment allocation of patients was applied by using Thompson's rule with fractional exponents $\kappa = 0.25, 0.50, 0.75$ and 1.00. Note that $\kappa = 0$ would correspond to treatment allocation by tossing a fair coin, and therefore the corresponding CDF of $S(200)$ would be very similar to that obtained under *BARTA* design (d).

The top part of Figure S3 shows how the application of *BARTA*, under $\mathbb{Q}_{null}$, leads to often allocating exactly half of the patients to both treatment arms, which happens in trial runs

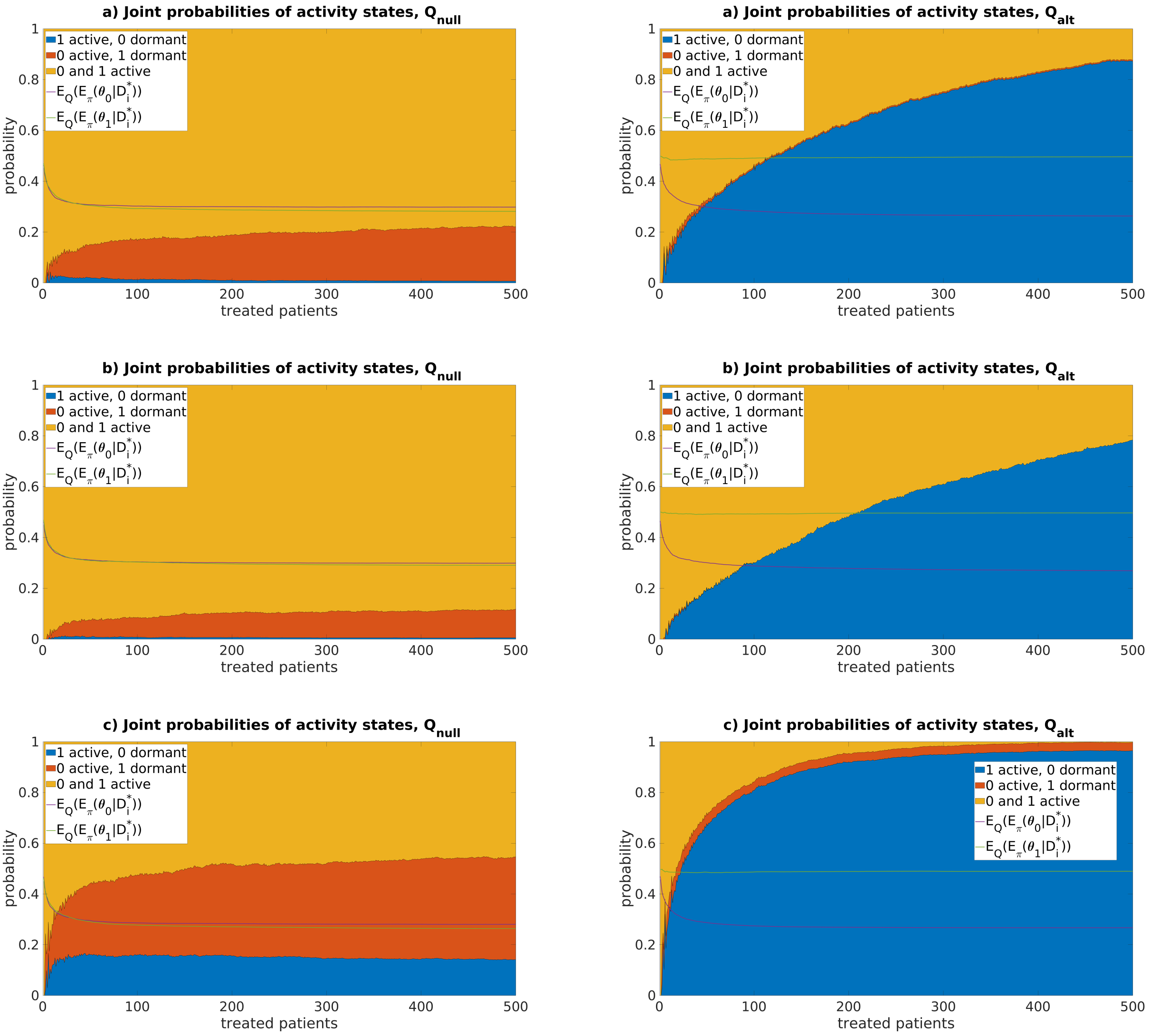

Figure S2: Effect of the choice of the values of the design parameters ε and δ on the joint activity states of the two treatment arms when applying *BARTA* for treatment allocation. Joint probabilities of the different combinations of active and dormant states are shown, as functions of the number i of treated trial participants. The results are based on 5000 simulated data sets of size $N_{\max} = 500$, under $\mathbb{Q}_{null}$ with true parameter values $\theta_0 = \theta_1 = 0.3$ (left) and $\mathbb{Q}_{alt}$ with values $\theta_0 = 0.3, \theta_1 = 0.5$ (right). Three combinations of the design parameters were used: (a) $\varepsilon = 0.1$, $\delta = 0.1$ (top), (b) $\varepsilon = 0.05$, $\delta = 0.1$ (middle), (c) $\varepsilon = 0.2$, $\delta = 0.05$ (bottom). Also shown are the expectations $\mathbb{E}_{\mathbb{Q}_{null}}\big(\mathbb{E}_\pi\big(\boldsymbol{\theta}_k|D_i^*\big)\big)$ and $\mathbb{E}_{\mathbb{Q}_{alt}}\big(\mathbb{E}_\pi\big(\boldsymbol{\theta}_k|D_i^*\big)\big), (1 \le i \le 500, k = 0, 1)$, computed from these simulations.

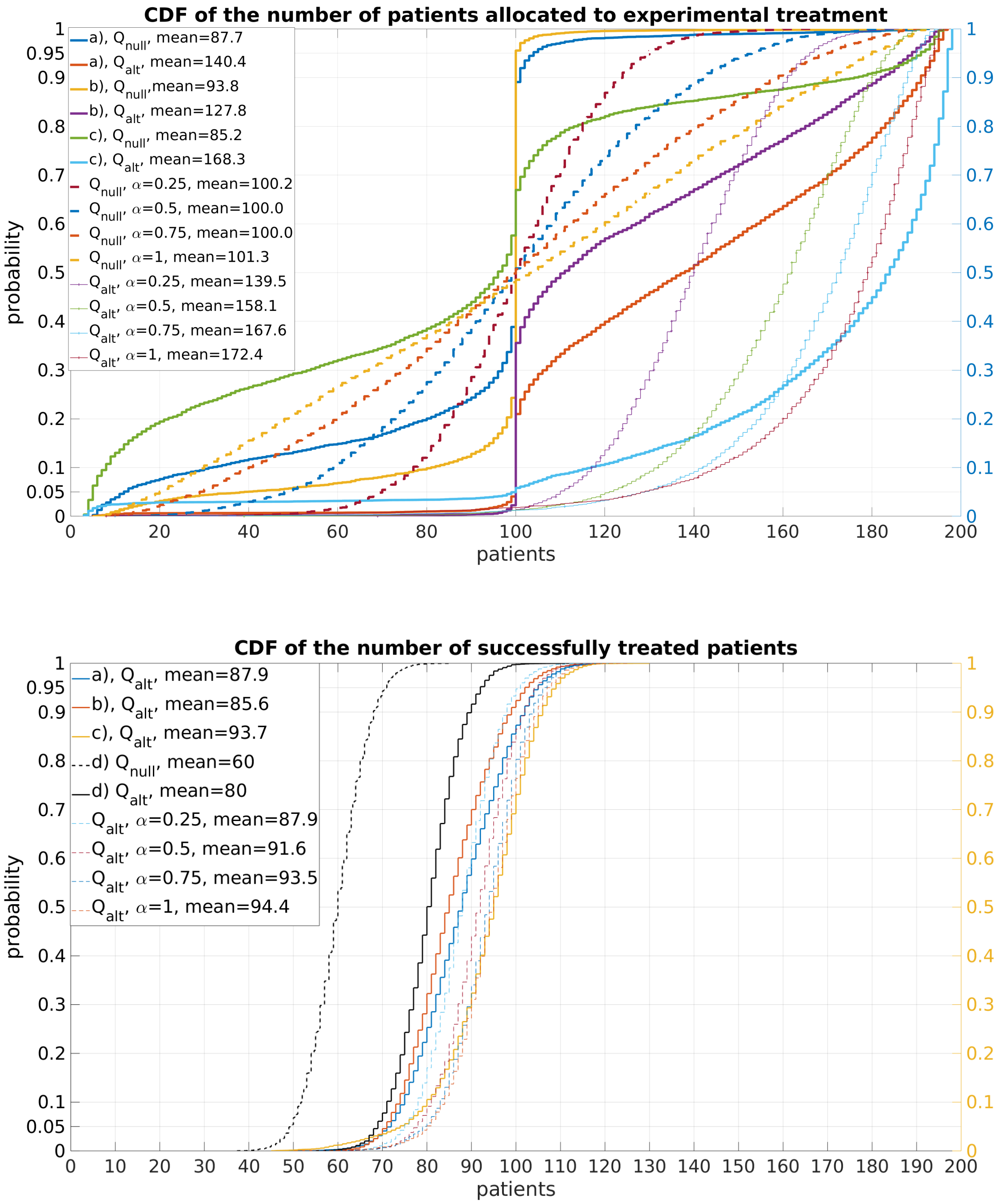

Figure S3: Effect of the choice of the design parameters ε and δ in *BARTA* on the number of patients allocated to the experimental treatment and on the total number of treatment successes. Cumulative distribution functions of $N_1(200)$ (top) and $S(200)$ (bottom) are shown, based on 5000 simulated data sets, under $\mathbb{Q}_{null}$ with true parameter values $\theta_0 = \theta_1 = 0.3$ and $\mathbb{Q}_{alt}$ with values $\theta_0 = 0.3, \theta_1 = 0.5$. Three combinations of the design parameters were used: (a) $\varepsilon = 0.1$, $\delta = 0.1$, (b) $\varepsilon = 0.05$, $\delta = 0.1$, (c) $\varepsilon = 0.2$, $\delta = 0.05$. In addition, (d) represents a completely symmetric treatment allocation. For comparison we also plot the corresponding CDF under the alternative hypothesis obtained by using fractional Thompson's rule with respective parameters $\kappa = 0.25, 0.5, 0.75$ and 1.

during which the dormant state had not been entered even once. Overall, due to the protective safety margin $\delta > 0$, *BARTA* has a tendency of allocating more patients to the control arm. For Thompson's rule, the distribution of $N_1(200)$ under $\mathbb{Q}_{null}$ is symmetric, but it has a large variance, signalling corresponding instability in treatment allocation.

Under $\mathbb{Q}_{alt}$, the better performance of the experimental treatment is usually detected rather early in the trial, and then, with more evidence from the data, all adaptive rules use progressively stronger control in directing patients to this better treatment. However, there is a small probability that, accidentally, more patients are given the inferior control treatment. It is clear, as is also illustrated by Figure S1, that the risk for this to happen is highest early in the trial when there are only few observed outcomes. In the present simulations, following *BARTA* with design parameters (a), (b) and (c), these $\mathbb{Q}_{alt}$-probabilities were, respectively, 0.041, 0.023 and 0.049.

In the bottom part of Figure S3, the CDFs for $S(200)$ under $\mathbb{Q}_{null}$ are identical in all designs, due to both treatment arms having the same true response rate 0.3. For $\mathbb{Q}_{alt}$, employing the symmetric block randomization scheme *BARTA* (d) gives $\mathbb{E}_{\mathbb{Q}_{alt}}(S(200)) = 80$. If all patients could be given the better experimental treatment, the resulting optimal expected value would be 100. In Figure S3, the expectations $\mathbb{E}_{\mathbb{Q}_{alt}}(S(200))$ for different adaptive schemes range from 85.6 for *BARTA* design (b), to 94.4 for Thompson's rule with $\kappa = 1$.

Employing an initial burn-in period. The potential problem of accidentally allocating more patients to an inferior treatment arm can be mitigated by delaying the workings of the adaptive mechanism of *BARTA*, or Thompson's rule, by employing the symmetric block randomization scheme (d) until a fixed number of patients have been assigned to all treatments. To have an idea of the size of the effect of this modification in the present example, we carried out a simulation study identical to that leading to Figure S3 except that, of the considered 200 patients, the first 30 were divided evenly to the two treatments, 15 to both. The result is shown in Figure S4.

The probabilities of imbalance in the unwanted direction are now lower, respectively 0.013, 0.005 and 0.019 for *BARTA* designs (a), (b) and (c). On the other hand, delaying the adaptive mechanism from taking effect until outcome data from the first 30 patients are available obviously lowers, in case of $\mathbb{Q}_{alt}$, the expected number of treatment successes by small amounts. Alternative versions of burn-in in adaptive designs have been considered, e.g., in Thall & Wathen [6] and Thall *et al.* [7].

B.1.3 Effect of adaptive treatment allocation on frequentist performance measures

There are no free lunches, and these potential gains in terms of either more efficacious treatments given to more patients in the trial, or smaller numbers of treated patients needed for being able to select the better treatment, are to be weighed against corresponding potentially stronger statistical inferences that might be obtained from more balanced designs. For a numerical comparison, we applied a design where adaptive patient allocation was applied following either *BARTA* or Thompson's rule, and an assessment of the results, including the possibility of dropping a treatment arm, was only allowed at the time at which a pre-specified number $i = N_{max}$ of patients had been treated. Here we consider the choice $N_{max} = 200$, reporting the results from experiments with $N_{max} = 100$ and 500 in part D of this Supplement.

In a trial with only two treatments, dropping either one is taken to mean selection of the other.

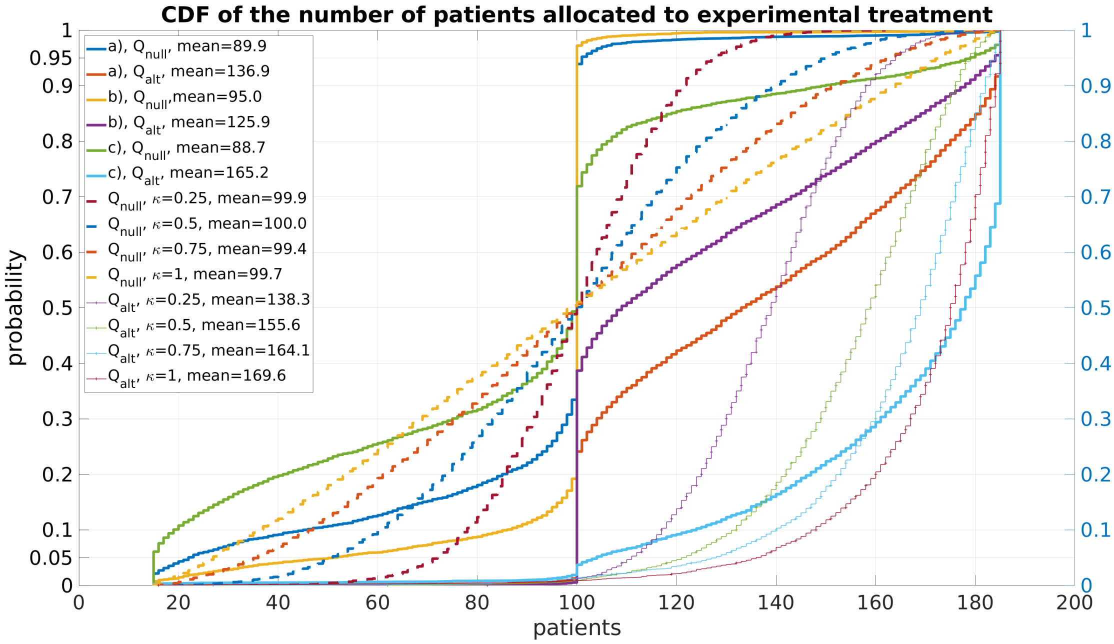

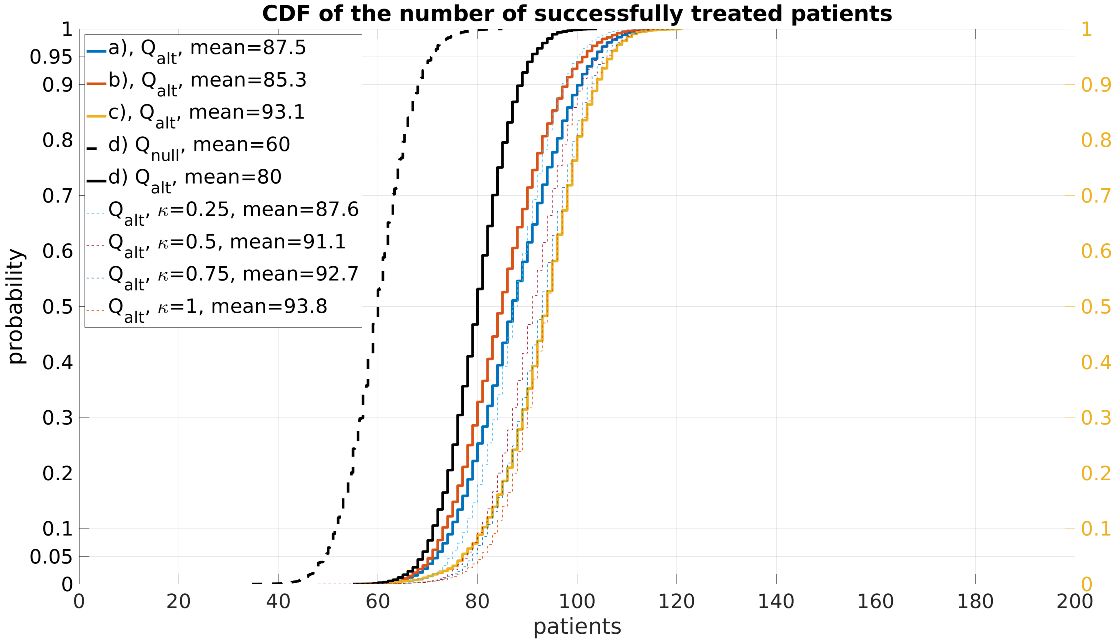

Figure S4: Effect of employing a symmetric burn-in period of $n_0 = 30$ patients in $BARTA$, on the number of patients allocated to the experimental treatment and on the total number of treatment successes. Cumulative distribution functions of $N_1(200)$ (top) and $S(200)$ (bottom) are shown, based on 5000 simulated data sets, under $\mathbb{Q}_{null}$ with true parameter values $\theta_0 = \theta_1 = 0.3$ and $\mathbb{Q}_{alt}$ with values $\theta_0 = 0.3, \theta_1 = 0.5$. Three combinations of the design parameters were used: (a) $\varepsilon = 0.1$, $\delta = 0.1$, (b) $\varepsilon = 0.05$, $\delta = 0.1$, (c) $\varepsilon = 0.2$, $\delta = 0.05$. In addition, (d) represents a completely symmetric treatment allocation. For comparison we also plot the corresponding CDF under the alternative hypothesis obtained by using fractional Thompson's rule with respective parameters $\kappa = 0.25, 0.5, 0.75$ and 1.

The final analysis made at N_{max} need not necessarily use the same threshold values as *BARTA*, and therefore we use new notations ε_0 and δ_0 for them. Accordingly, when performing such an analysis at $i = N_{max}$, the control arm is dropped if $\mathbb{P}_\pi(\boldsymbol{\theta}_0 + \delta_0 \geq \boldsymbol{\theta}_1 | D^*_{N_{max}}) \leq \varepsilon_0$ and the experimental arm if $\mathbb{P}_\pi(\boldsymbol{\theta}_1 \geq \boldsymbol{\theta}_0 | D^*_{N_{max}}) \leq \varepsilon_0$. Obviously, at most one of these criteria can be satisfied for given data $D^*_{N_{max}}$ when $\varepsilon_0 < 0.5$. But it is also possible that neither of them is satisfied, in which case no firm decision concerning treatment selection is made at N_{max}.

Even then, however, there is the possibility of studying the joint posterior $\mathbb{P}_\pi((\boldsymbol{\theta}_0, \boldsymbol{\theta}_1) \in \cdot | D^*_{N_{max}})$ for the purpose of drawing further inferences from the results of the trial. For example, one can print the posterior CDF $\mathbb{P}_\pi(\boldsymbol{\theta}_1 - \boldsymbol{\theta}_0 \leq x | D^*_{N_{max}}), -1 \leq x \leq 1$, and then decide, the study protocol permitting this, whether to continue the trial by recruiting more participants. This may then lead to either one of the two selection criteria being satisfied at a later point in time.

We now study how the application of different versions of adaptive treatment allocation influences the strength of statistical inferences, viewed from a frequentist perspective, that can be drawn from trial data in Experiment 1. For this, we consider the probabilities $\mathbb{Q}(\mathbb{P}_\pi(\boldsymbol{\theta}_0 + \delta_0 \geq \boldsymbol{\theta}_1 | \mathbf{D}^*_{N_{max}}) \leq \varepsilon_0)$ and $\mathbb{Q}(\mathbb{P}_\pi(\boldsymbol{\theta}_1 \geq \boldsymbol{\theta}_0 | \mathbf{D}^*_{N_{max}}) \leq \varepsilon_0)$ for both $\mathbb{Q} = \mathbb{Q}_{null}$ and $\mathbb{Q} = \mathbb{Q}_{alt}$, choosing $\delta_0 = 0.05$ and $\varepsilon_0 = 0.05$. We applied *BARTA* with design parameters (a), (b), (c) and (d), and Thompson's rule with fractional exponents $\kappa = 0.25, 0.50, 0.75$ and 1.0. Our simulation experiment consisted of 5000 repetitions of a trial up to 500 patients. From each simulated data set we then computed numerical values for the posterior probabilities $\mathbb{P}_\pi\big(\boldsymbol{\theta}_1 \geq \boldsymbol{\theta}_0 \big| D^*_{N_{max}}\big)$ and $\mathbb{P}_\pi\big(\boldsymbol{\theta}_0 + \delta_0 \geq \boldsymbol{\theta}_1 \big| D^*_{N_{max}}\big)$, and drew, at $N_{max} = 200$, the resulting CDFs under $\mathbb{Q}_{null}$ and $\mathbb{Q}_{alt}$, shown in Figure S5. The corresponding figures at $N_{max} = 100$ and $N_{max} = 500$ are included in part D of this Supplement as Figures S12 and S13.

Under $\mathbb{Q}_{null}$, the CDFs of $\mathbb{P}_\pi\big(\boldsymbol{\theta}_1 \geq \boldsymbol{\theta}_0 \big| \mathbf{D}^*_{200}\big)$ for different designs, shown in the bottom part of Figure S5, are almost linear, which would correspond to the *Uniform*$(0, 1)$ sampling distribution and a correspondingly large variance. This is the case particularly in the designs following Thompson's rule, where the two treatment arms are considered symmetrically. For *BARTA* the deviations from linearity are clearer, and most evident in the case of *BARTA* design (c). The overall shape of the CDFs of $\mathbb{P}_\pi\big(\boldsymbol{\theta}_0 + 0.05 \geq \boldsymbol{\theta}_1 \big| \mathbf{D}^*_{200}\big)$ in the top part of Figure S5 is convex, signalling that the $\mathbb{Q}_{null}$-density of these posterior probabilities tends to increase as their values increase. The reason is the threshold $\delta_0 = 0.05$ providing extra protection for the control arm arm against being dropped.

The CDFs generated under $\mathbb{Q}_{alt}$ behave very differently. Those of $\mathbb{P}_\pi\big(\boldsymbol{\theta}_1 \geq \boldsymbol{\theta}_0 \big| \mathbf{D}^*_{200}\big)$ in the bottom part of Figure S5 show a high concentration of values close to 1, and those of $\mathbb{P}_\pi\big(\boldsymbol{\theta}_0 + 0.05 \geq \boldsymbol{\theta}_1 \big| \mathbf{D}^*_{200}\big)$ in the top part of Figure S5 a somewhat lower but still high concentration close to 0. The main difference between these CDFs stems from the opposite directions of the inequalities between $\boldsymbol{\theta}_0$ and $\boldsymbol{\theta}_1$, and the difference in concentration is again due to the threshold $\delta_0 = 0.05$.

Based on these results, we then computed numerical values for the commonly used operating characteristics, the true and false positive and negative rates, shown in Table S1. More exactly, we use the following terminology:

false positive rate $= \mathbb{Q}_{null}(\mathbb{P}_\pi(\boldsymbol{\theta}_0 + \delta_0 \geq \boldsymbol{\theta}_1 | \mathbf{D}^*_{N_{max}}) \leq \varepsilon_0)$,

true negative rate $= \mathbb{Q}_{null}(\mathbb{P}_\pi(\boldsymbol{\theta}_1 \geq \boldsymbol{\theta}_0 | \mathbf{D}^*_{N_{max}}) \leq \varepsilon_0)$,

true positive rate $= \mathbb{Q}_{alt}(\mathbb{P}_\pi(\boldsymbol{\theta}_0 + \delta_0 \geq \boldsymbol{\theta}_1 | \mathbf{D}^*_{N_{max}}) \leq \varepsilon_0)$ and

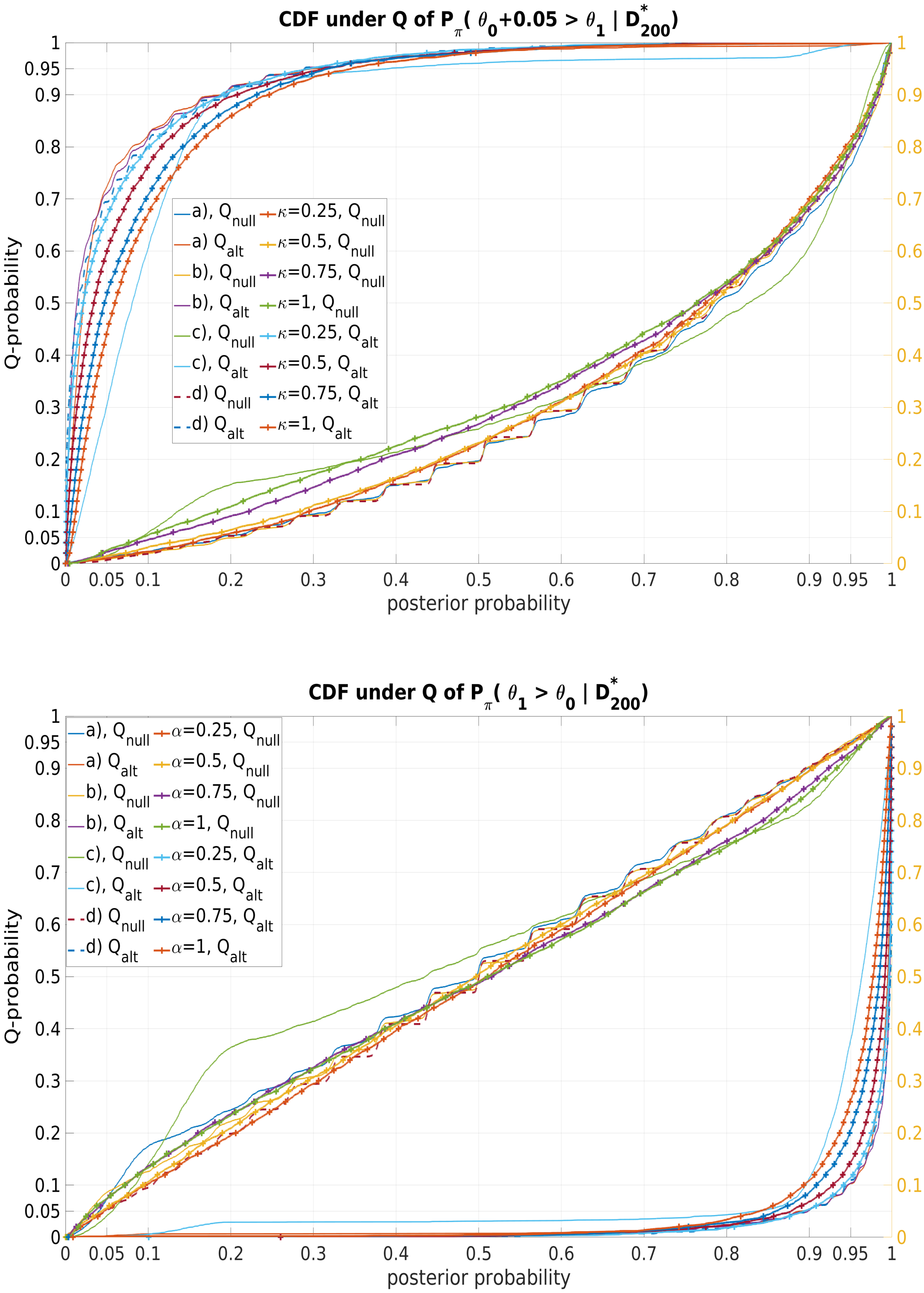

Figure S5: Effect of the design parameters ε and δ of *BARTA*, and κ of Thompson's rule, on the CDFs of the posterior probabilities $\mathbb{P}\big(\theta_0 + 0.05 \geq \theta_1 \big| D^*_{200}\big)$ (top) and $\mathbb{P}\big(\theta_1 \geq \theta_0 \big| D^*_{200}\big)$ (bottom) in the 2-arm trial of Experiment 1 when applying *BARTA* for treatment allocation and making a final assessment at $i = N_{\max} = 200$. The results are based on 5000 data sets generated under $\mathbb{Q}_{null}$ and $\mathbb{Q}_{alt}$ when using the following combinations of design parameters: (a) $\varepsilon = 0.1, \delta = 0.1$, (b) $\varepsilon = 0.05, \delta = 0.1$, (c) $\varepsilon = 0.2, \delta = 0.05$.

$\varepsilon_0 = 0.05, \delta_0 = 0.05$	(a)	(b)	(c)	(d)	$\kappa = 0.25$	$\kappa = 0.5$	$\kappa = 0.75$	$\kappa = 1$
$\mathbb{Q}_{null}$: false positive	0.014	0.009	0.014	0.007	0.011	0.014	0.023	0.025
$\mathbb{Q}_{null}$: true negative	0.074	0.086	0.040	0.052	0.054	0.056	0.073	0.074
$\mathbb{Q}_{null}$: inconclusive	0.912	0.906	0.946	0.941	0.935	0.929	0.904	0.901
$\mathbb{Q}_{alt}$: true positive	0.723	0.711	0.303	0.694	0.665	0.598	0.516	0.443
$\mathbb{Q}_{alt}$: false negative	0.002	0.001	0.000	~ 0	~ 0	~ 0	0.001	0.001
$\mathbb{Q}_{alt}$: inconclusive	0.275	0.288	0.696	0.306	0.335	0.402	0.483	0.555

Table S1: True and false positive and negative rates when applying *BARTA* or Thompson's rule and threshold values $\varepsilon_0 = 0.05$ and $\delta_0 = 0.05$ in a trial of size $N_{max} = 200$.

false negative rate $= \mathbb{Q}_{alt}(\mathbb{P}_\pi(\boldsymbol{\theta}_1 \geq \boldsymbol{\theta}_0 | \mathbf{D}^*_{N_{max}}) \leq \varepsilon_0)$.

In addition, the probabilities $\mathbb{Q}(\mathbb{P}_\pi(\boldsymbol{\theta}_0 + \delta_0 \geq \boldsymbol{\theta}_1 | \mathbf{D}^*_{N_{max}}) > \varepsilon_0, \mathbb{P}_\pi(\boldsymbol{\theta}_1 \geq \boldsymbol{\theta}_0 | \mathbf{D}^*_{N_{max}}) > \varepsilon_0)$ are called *inconclusive rates*, respectively, under $\mathbb{Q} = \mathbb{Q}_{null}$ and $\mathbb{Q} = \mathbb{Q}_{alt}$.

The following conclusions are now immediate from Table S1. For $N_{max} = 200$, the false positive rates are small, below 2.5 percent, for all considered versions of adaptive treatment allocation. This is true even for the "liberal" design parameters (c) in *BARTA* for which there was a non-negligible probability, about five percent, of serious imbalance in treatment allocation in the unwanted direction. The false negative rates are very small for all considered designs. Under $\mathbb{Q}_{null}$, the trial remains inconclusive with probability at least ninety percent, which is consistent with the fact that then there is no difference between the true response rates θ_0 and θ_1. Finally, the true positive rate (*power*) is on the moderate level of approximately seventy percent when applying *BARTA* with design parameter values (a), (b) and (d), and almost as high for Thompson's rule with $\kappa = 0.25$. Recall here that (d) means symmetric block randomization, which can thought to provide a suitable yardstick for such comparisons of power. For larger values of κ, for which the adaptive mechanism is stronger, these rates are smaller. Of all considered alternatives, the smallest true positive rate is obtained for the design parameters (c).

Corresponding tables for $N_{max} = 100$ and $N_{max} = 500$ are provided, with comments, as Table S2 and Table S3 in Section D of this Supplement.

Employing an initial burn-in period. We also studied the effect of the burn-in period, described above in subsection A.1.2, on the frequentist performance measures in Table S1. For this, we drew CDFs (not shown) similar to those in Figure S5, and then worked out numerical values for the true and false positive and negative rates. The results, with some comments, can be found in Table S4 in Section D.

Remarks on other test variants. Somewhat different numerical values are obtained if the positive safety margin δ_0 protecting the control arm from being dropped is given the value $\delta_0 = 0$. With this extra protection removed, the rates of positive findings, both true and false, will naturally increase, while the rates of negative results remain unchanged. Another modification is to change the presently used decision criterion $\mathbb{P}_\pi(\boldsymbol{\theta}_1 \geq \boldsymbol{\theta}_0 | \mathbf{D}^*_{N_{max}}) \leq \varepsilon_0$ for dropping the experimental arm into $\mathbb{P}_\pi(\boldsymbol{\theta}_1 \geq \boldsymbol{\theta}_0 + \delta_0 | \mathbf{D}^*_{N_{max}}) \leq \varepsilon_0$, in which case it would be symmetric to the condition $\mathbb{P}_\pi(\boldsymbol{\theta}_0 + \delta_0 \geq \boldsymbol{\theta}_1 | \mathbf{D}^*_{N_{max}}) \leq \varepsilon_0$ for dropping the control arm. If made, such a conclusion (in effect, declaring *futility*) is made more easily. The true and false

negative rates become then larger, while the rates of positive results remain unaltered. For both variants, the inconclusive rates are larger than when applying the original criteria. Numerical values for these two variants, with $N_{max} = 200$, are provided in Section D in Tables S5 and S6.

It depends on the concrete context whether either one of these alternative criteria would be considered more appropriate than the version used in the construction of Table S1. All three represent different forms of *superiority* trials. After a suitable modification in the definition of the posterior probabilities that are considered, the same basic ideas and algorithms would also apply in *non-inferiority* and *equivalence* trials (e.g., Lesaffre [1]).

B.1.4 Effect of the design parameters on adaptive treatment selection

We then modified the design by employing the adaptive *BARTS* algorithm for treatment selection. In the definition of *BARTS*, $n_{k,last}$ is the last list index value for which treatment arm k has not been dropped from the trial, $0 \leq k \leq K$. Denote then by $N_{k,last}$ the index of the last patient receiving treatment k. Figure S6 shows the probabilities $\mathbb{Q}(N_{0,last} \leq i, N_{1,last} > i)$ of having dropped the control arm, $\mathbb{Q}(N_{0,last} > i, N_{1,last} \leq i)$ of having dropped the experimental arm, and $\mathbb{Q}(N_{0,last} > i, N_{1,last} > i)$ of not having done either of these, all considered at the time i patients had been treated. Note that, since the possibility of dropping both treatment arms in the same trial has been ruled out, the first two probabilities can be written in the shorter form $\mathbb{Q}(N_{0,last} \leq i)$ and $\mathbb{Q}(N_{1,last} \leq i)$. Empirical estimates of these probabilities are shown, based on 5000 simulated samples from $\mathbb{Q}_{null}$ (left) and $\mathbb{Q}_{alt}$ (right). The earlier threshold values (a), (b) and (c) for ε and δ were again applied, but combining them with $\varepsilon_1 = 0$ and $\varepsilon_2 = 0.05$ for *BARTS*.

In Figure S6, on the left, the curve $\mathbb{Q}_{null}(N_{0,last} \leq i), 1 \leq i \leq 500$, forming the upper boundary of the blue band "1 active, 0 dropped", depicts the false positive rate evaluated at i. On the right, $\mathbb{Q}_{alt}(N_{0,last} \leq i)$ is the true positive rate, or power at i. The widths of the brown bands in this figure can be interpreted similarly, with $\mathbb{Q}_{null}(N_{1,last} \leq i)$ on the left being the true negative rate evaluated at i, and $\mathbb{Q}_{alt}(N_{1,last} \leq i)$ on the right the false negative rate. The latter probabilities are small, at most 0.05 for the considered design parameter values (a), (b) and (c). The widths of the yellow bands represent the inconclusive rates at i.

From this follows that also the areas of these colored bands Figure S6 have meaningful interpretations in terms of expected values. The area of the blue region from 1 to i is the expected value, with respect to $\mathbb{Q}_{null}$ (left) and to $\mathbb{Q}_{alt}$ (right), of the number of patients among the first i, who were directed to the experimental treatment in the situation in which the control arm had already been dropped. The areas of the brown bands can be interpreted in a similar fashion, with the roles of the two treatments interchanged. The area of the yellow band from 1 to i is the expected value, again with respect to $\mathbb{Q}_{null}$ (left) and to $\mathbb{Q}_{alt}$ (right), of the random variable $\min\{N_{0,last}, N_{1,last}, i\}$.

Finally, Figure S6 shows the expectations of $\mathbb{E}_\pi\big(\boldsymbol{\theta}_k|D_i^*\big)$ and $\mathbb{E}_\pi\big(\boldsymbol{\theta}_k|D_i^*\big), (1 \leq i \leq 500, k = 0, 1)$, computed from these simulations under $\mathbb{Q}_{null}$ and $\mathbb{Q}_{alt}$. Overall, the behaviour of these curves is similar to those in Figure S2, although the negative bias seems here slightly larger. Apparently, this difference is due to *BARTS* imposing a stronger control on treatment allocation.

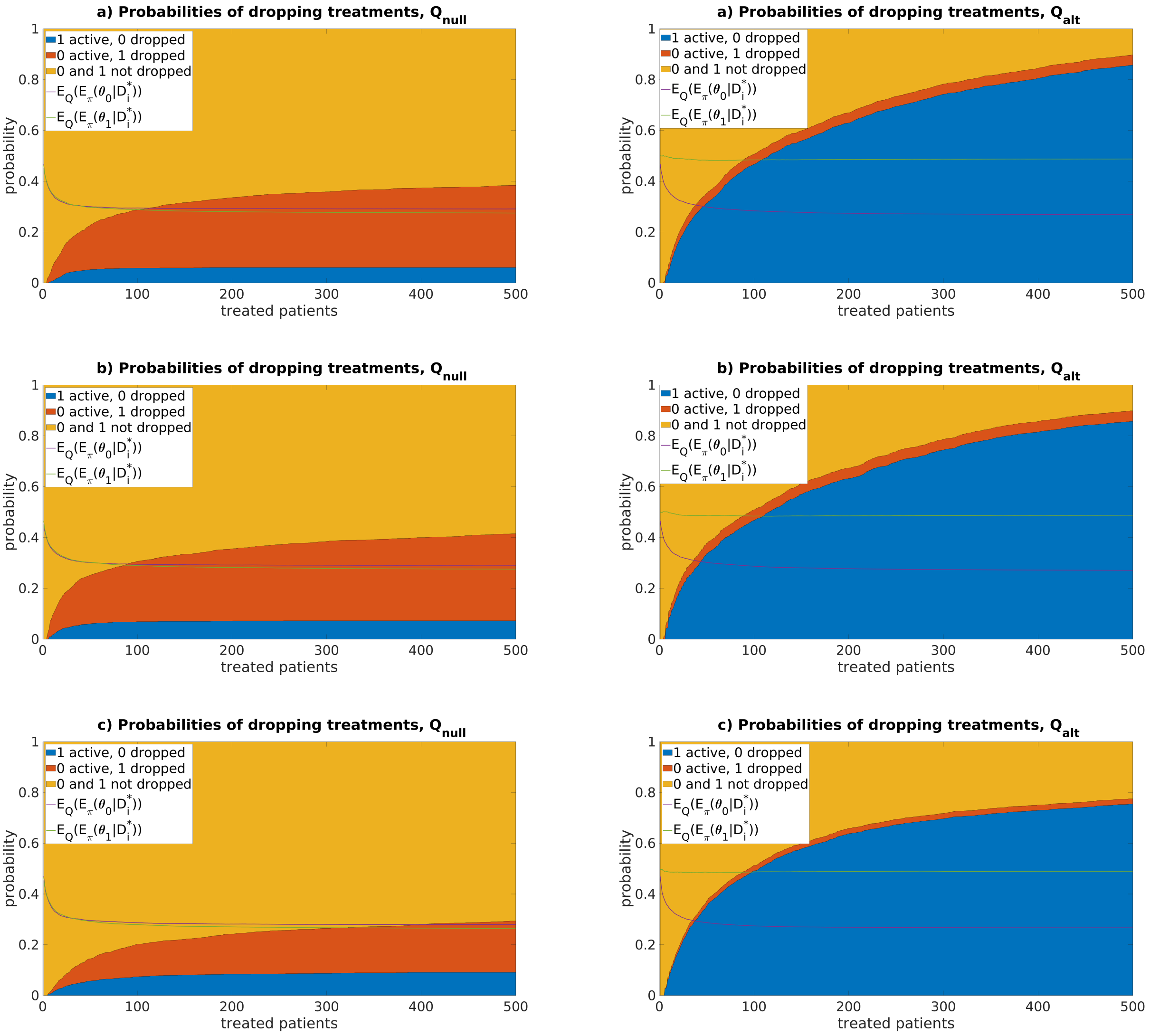

Figure S6: Probabilities of dropping treatments when applying *BARTS*, together with expectations of the success parameters, both shown as functions of the cumulative number of treated patients. The results are based on 5000 simulated data sets of size $N_{\max} = 500$, under $\mathbb{Q}_{null}$ with true parameter values $\theta_0 = \theta_1 = 0.3$ (left) and $\mathbb{Q}_{alt}$ with values $\theta_0 = 0.3, \theta_1 = 0.5$ (right). Three combinations of design parameters were considered: (a) $\varepsilon = 0.1, \varepsilon_1 = 0, \varepsilon_2 = 0.05$, $\delta = 0.1$ (top), (b) $\varepsilon = 0.05, \varepsilon_1 = 0, \varepsilon_2 = 0.05$, $\delta = 0.1$ (middle), (c) $\varepsilon = 0.2, \varepsilon_1 = 0, \varepsilon_2 = 0.05$, $\delta = 0.05$ (bottom). Also shown are the expectations $\mathbb{E}_{\mathbb{Q}_{null}}\left(\mathbb{E}_\pi\left(\boldsymbol{\theta}_k|D_i^*\right)\right)$ and $\mathbb{E}_{\mathbb{Q}_{alt}}\left(\mathbb{E}_\pi\left(\boldsymbol{\theta}_k|D_i^*\right)\right), (1 \leq i \leq 500, k = 0, 1)$, computed from these simulations. For more details, see text.

B.2 Simulation studies with a 4-arm trial: Experiment 2

Our second simulation experiment is modeled following the set-up of Table 7 in Villar *et al.* [9], describing a trial with $K = 3$ experimental arms and a control arm. The hypotheses were $H_0 : \theta_k \leq \theta_0$ for all $k, 1 \leq k \leq 3$, and its logical complement $H_1 : \theta_k > \theta_0$ for at least one $k, 1 \leq k \leq 3$. Considered as a multiple hypothesis testing problem, applying significance level $\alpha = 0.05$ and the Bonferroni correction, H_0 was tested separately against each alternative $H_{1k} : \theta_k > \theta_0$ at level $\alpha/3$. The numerical results shown in Table 7 of Villar *et al.* [9] were based on using the fixed trial size of $N_{\max} = 80$, together with parameter values $(\theta_0, \theta_1, \theta_2, \theta_3) = (0.3, 0.3, 0.3, 0.3)$ for computing the family-wise error rate (FWER), and $(\theta_0, \theta_1, \theta_2, \theta_3) = (0.3, 0.4, 0.5, 0.6)$ for computing the power of concluding H_1. The small trial size was justified by thinking of a rare disease setting, where the number of patients in the trial could be a large proportion of all patients with the considered condition. Below, we continue using the shorthand notations $\mathbb{Q}_{null}$ and $\mathbb{Q}_{alt}$ for these two parameter settings.

B.2.1 Monitoring the operation of *BARTA* in Experiment 2

As in Experiment 1, we first monitored the execution of this trial, based on a single simulation from $\mathbb{Q}_{alt}$, and thereby applying *BARTA* for treatment allocation with threshold values $\varepsilon = 0.1$ and $\delta = 0.1$. Figure S7 presents an example based on such simulated data, showing the time-evolution of the posterior probabilities $\mathbb{P}_\pi\big(\boldsymbol{\theta}_0 + \delta \geq \boldsymbol{\theta}_\vee \big| D_i^*\big)$ and $\mathbb{P}_\pi\big(\boldsymbol{\theta}_k = \boldsymbol{\theta}_\vee \big| D_i^*\big), 1 \leq k \leq 3$, of the the posterior expectations $\mathbb{E}_\pi\big(\boldsymbol{\theta}_k | D_i^*\big)$ and of the cumulative sums of the activity indicators I_k, $0 \leq k \leq 3$, all considered at times at which i patients had been treated and up to maximal trial size $N_{\max} = 500$.

From the top display we can see how, with some luck in the simulation that was carried out, the posterior probabilities $\mathbb{P}_\pi\big(\boldsymbol{\theta}_0 + \delta \geq \boldsymbol{\theta}_\vee \big| D_i^*\big), \mathbb{P}_\pi\big(\boldsymbol{\theta}_1 = \boldsymbol{\theta}_\vee \big| D_i^*\big)$ and $\mathbb{P}_\pi\big(\boldsymbol{\theta}_2 = \boldsymbol{\theta}_\vee \big| D_i^*\big)$ started progressively to take on values below the given threshold $\varepsilon = 0.10$ and finally stayed there during the remaining simulation run. In contrast, after considerable early variation, the posterior probabilities $\mathbb{P}_\pi\big(\boldsymbol{\theta}_3 = \boldsymbol{\theta}_\vee \big| D_i^*\big)$ corresponding to the highest true response rate $\theta_3 = 0.6$ stayed consistently above that threshold level, and actually started to dominate the others from approximately $i = 120$ onward. The cumulative activity indicators for all treatment arms in the bottom display of Figure S7 show clearly when each of these arms was active or dormant. In this simulation, there was some back-and-forth movement between these two states, but finally treatment arms $0, 1$ and 2, respectively after $153, 174$, and 68 treated patients, remained dormant. The dotted line shows the cumulative numbers of successes in the simulation, ending up with the total $S(500) = 294$, not much short of the optimal expected value 300 that would have been obtained if all 500 patients had been allocated to the best treatment with success rate $\theta_3 = 0.6$.

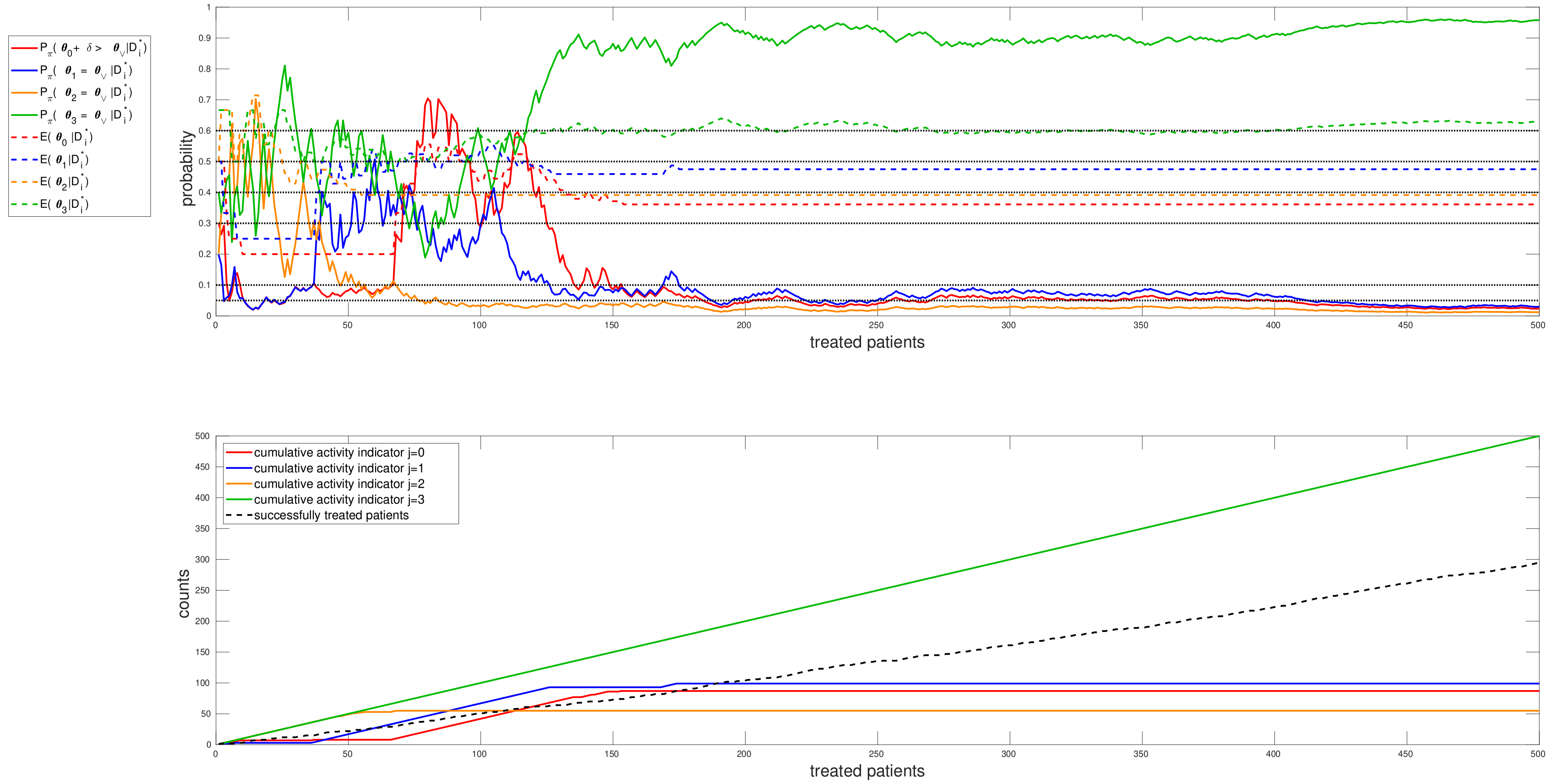

Figure S7: An example of monitoring the execution of a 4-arm trial in a simulated clinical trial of size $N_{\max} = 500$ when applying *BARTA* for treatment allocation. Top: Time-evolution of the posterior probabilities $\mathbb{P}_\pi(\theta_0 + \delta \geq \theta_\vee | D_i^*)$ and $\mathbb{P}_\pi(\theta_k = \theta_\vee | D_i^*), 1 \leq k \leq 3$, and of the posterior expectations $\mathbb{E}_\pi(\theta_k | D_i^*)$, $0 \leq k \leq 3; 1 \leq i \leq 500$. Bottom: Cumulative sums of activity indicators I_k, $0 \leq k \leq 3$, and of the total number of successful treatments, both as functions of the number of treated patients. The simulation was performed with true parameter values $\theta_0 = 0.3, \theta_1 = 0.4, \theta_2 = 0.5$ and $\theta_3 = 0.6$, by using *BARTA* with design parameters $\varepsilon = 0.1$ and $\delta = 0.1$. For more details, see text.

B.2.2 Effect of the design parameters on treatment allocation

Next, as in Experiment 1, we studied the effect of the choice of the design parameters ε and δ in *BARTA* on some selected frequentist type key characteristics of the trial. For this, we simulated 2000 data sets of size $N_{\max} = 500$, under both $\mathbb{Q}_{null}$ and $\mathbb{Q}_{alt}$. The same three combinations of the design parameters were used as before: **a)** $\varepsilon = 0.1$, $\delta = 0.1$, **b)** $\varepsilon = 0.05$, $\delta = 0.1$, **c)** $\varepsilon_1 = 0.2$, $\delta = 0.05$. For the analysis, $\boldsymbol{\theta}_0, \ldots, \boldsymbol{\theta}_3$ were assumed to be a priori independent and uniformly distributed on $(0, 1)$.

In a 4-arm trial there would in principle be $2^4 - 1 = 15$ possibilities of forming combinations of active and dormant states at a given i, and it would be hard to present such results in an easily understandable graphical form. The main aim of the trial of this type is to find out whether one of the experimental treatments would be better than the others, and in particular, better than the control. In view of this, we call treatment k *maximal at i* if $\mathbb{P}_\pi\big(\boldsymbol{\theta}_k = \boldsymbol{\theta}_\vee | D_i^*\big) \geq \mathbb{P}_\pi\big(\boldsymbol{\theta}_\ell = \boldsymbol{\theta}_\vee | D_i^*\big)$ $\forall \ell \neq k$, and then focus our attention on events of the form {treatment k is maximal, control treatment is dormant}. The results are shown in Figure S8. In the subfigures, the width of each of the 4 bands at i corresponds to the $\mathbb{Q}$-probability of a respective event. The three lower bands represent the $\mathbb{Q}$-probabilities of {treatment k is maximal at i, $I_{0,i} = 0$}, $1 \leq k \leq 3$, and the upper band (violet) the $\mathbb{Q}$-probabilities of $\{I_{0,i} = 1\}$.

In the present 4-arm experiment, we can think of all three experimental arms combined as competing, and being evaluated against, the control arm, in a way analogous to the single experimental treatment in Experiment 1. Seen from this angle, the sum of the widths of the three lower bands of Figure S8 corresponds to the width of the lowest band in Figure S2, while that of the top one in the former corresponds to the sum of the top two in the latter.

On the left of Figure S8, describing $\mathbb{Q}_{null}$, the violet band corresponding to $\{I_{0,i} = 1\}$ is broader than the other three, not only because the assumed initial state $\{I_{0,1} = 1\}$, but because the control arm is protected by $\delta = 0.1$ against being moved to the dormant state. The other three bands are similar to each other due to the assumed symmetry of the experimental treatments 1, 2 and 3 under $\mathbb{Q}_{null}$. All these probabilities stabilize rather quickly with growing i, well before $i = 100$.

On the right, corresponding to $\mathbb{Q}_{alt}$, the violet band becomes narrower with growing i, losing ground mainly to the yellow band, which represents the $\mathbb{Q}_{alt}$-probabilities of the events {treatment 3 is maximal at i, $I_{0,i} = 0$}. The widths of the three lower bands, yellow, brown and blue, are seen to follow the same order as the corresponding true response parameter values. Approximate values of these probabilities can be read from Figure S8 as well. For example, considering design (a) at $i = 500$, we get $\mathbb{Q}_{alt}$(treatment 3 is maximal at $500, I_{0,500} = 0) = 0.763$. Overall, designs (a) and (b) led to very similar $\mathbb{Q}_{alt}$-probabilities, while the more liberal design (c), which allowed for more variability during the early stages of the trial, gave rise to somewhat broader brown and blue bands.

B.2.3 Effect of the design parameters on treatment selection

We then employed also *BARTS*, in order to study the ability of this algorithm to drop possibly inferior treatment arms from the trial and thereby to act as a selection mechanism for those performing better. Using data simulated under $\mathbb{Q}_{null}$ and $\mathbb{Q}_{alt}$, the same three combinations of design parameters as in Experiment 1 were again considered: (a) $\varepsilon = 0.1, \varepsilon_1 = 0, \varepsilon_2 = 0.05$,

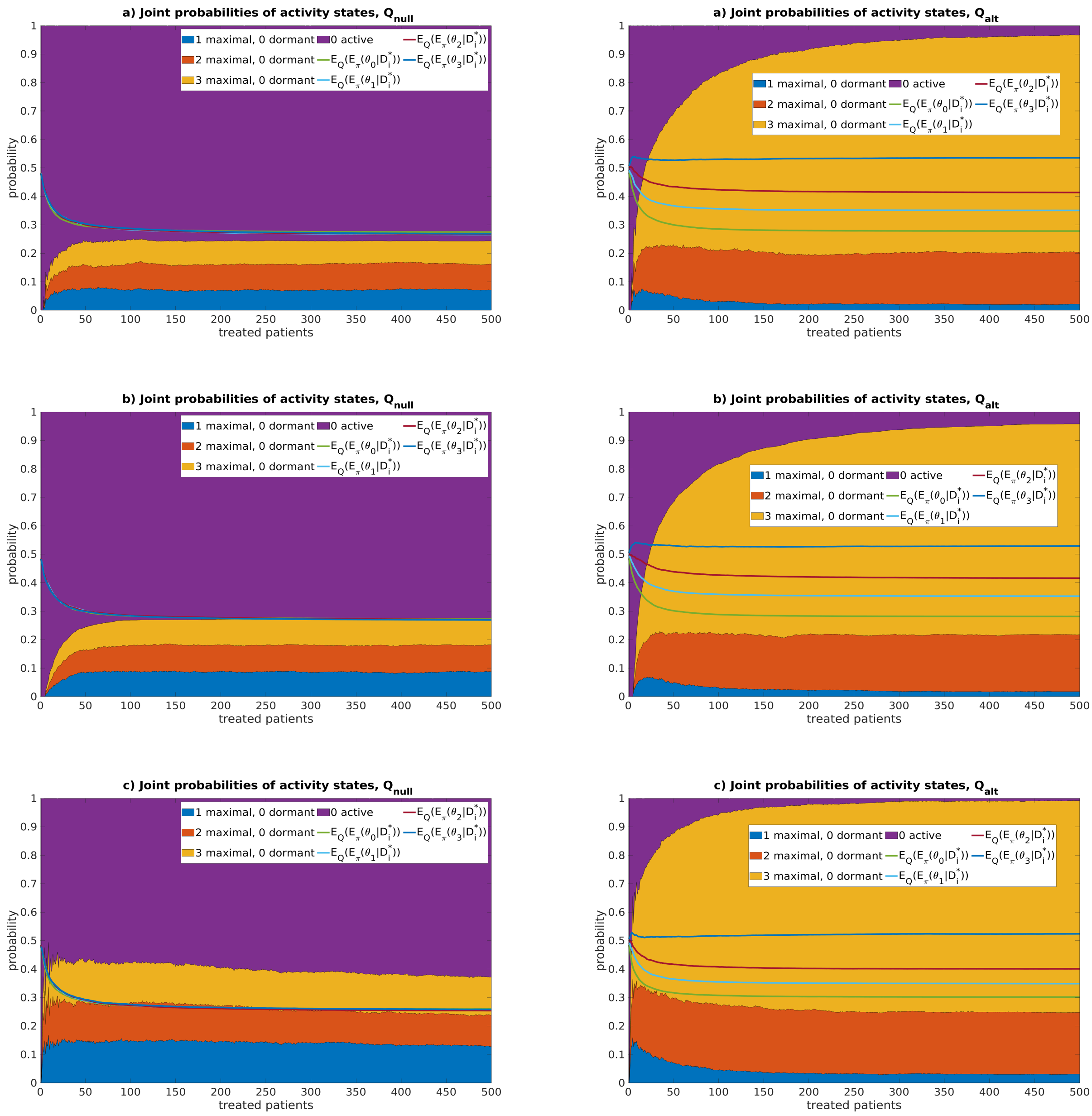

Figure S8: Effect of the choice of the values of the design parameters ε and δ in the 4-arm trial of Experiment 2 when applying *BARTA* for treatment allocation. Joint probabilities of some combinations of active and dormant states are shown, as functions of the number i of treated patients. The results are based on 2000 data sets of size $N_{\max} = 500$, under $\mathbb{Q}_{null}$ (left) and $\mathbb{Q}_{alt}$ (right). Three combinations of the design parameters were used: (a) $\varepsilon = 0.1$, $\delta = 0.1$ (top), (b) $\varepsilon = 0.05$, $\delta = 0.1$ (middle), (c) $\varepsilon_1 = 0.2$, $\delta = 0.05$ (bottom). In the subfigures, the width of each of the 4 bands corresponds to the $\mathbb{Q}$-probability of a respective event in the box. Also shown are the expectations $\mathbb{E}_{\mathbb{Q}_{null}}\big(\mathbb{E}_\pi\big(\boldsymbol{\theta}_k|D_i^*\big)\big)$ and $\mathbb{E}_{\mathbb{Q}_{alt}}\big(\mathbb{E}_\pi\big(\boldsymbol{\theta}_k|D_i^*\big)\big)$, $(1 \le i \le 500, 1 \le k \le 3)$, computed from these simulations. For more details, see text.

$\delta = 0.1$, (b) $\varepsilon = 0.05, \varepsilon_1 = 0, \varepsilon_2 = 0.05$, $\delta = 0.1$, (c) $\varepsilon = 0.2, \varepsilon_1 = 0, \varepsilon_2 = 0.05$, $\delta = 0.05$.

The results are shown in Figure S9. The main distinction to Figure S8 is that, in the definition of the four colored bands, the events $\{I_{0,i} = 0\}$ have here been replaced by $\{N_{0,last} \leq i\}$. The widths of the three lowest bands therefore represent the $\mathbb{Q}$-probabilities of the events $\{\text{treatment } k \text{ is maximal at } i, N_{0,last} \leq i\}$, $1 \leq k \leq 3$, while that of the violet band is the $\mathbb{Q}$-probability of $\{N_{0,last} > i\}$. As in the case of Experiment 1, these events have operational meanings comparable to corresponding key concepts used in hypothesis testing. Thus, on the left of Figure S9, the sum of the three lower bandwidths at i is the false positive rate when observing outcome data from i patients. If its size is of major concern to a person considering the design from a frequentist perspective, it can be made somewhat smaller in a similar fashion as suggested in the context of Experiment 1, by employing a form of a burn-in period and activating adaptive treatment allocation only after some fixed number of patients have been treated in all four arms.

C Additional figures to subsection B.1.2

Figures S10 and S11 below complement Figure S3, where we illustrated the effect of the design parameters of *BARTA* and Thompson's rule on treatment allocation in a two-arm trial with $N_{max} = 200$, and on the consequent total number of treatment successes. Here we do the same for $N_{max} = 100$ in Figure S10 and for $N_{max} = 500$ in Figure S11.

For data generated under $\mathbb{Q}_{null}$, the overall shape of the CDFs in Figures S10 and S11 remains remarkably close to that in Figure S3, where the trial size was $N_{max} = 200$. The differences become more evident when considering $\mathbb{Q}_{alt}$, in which case the adaptive rules can use their potential to allocate more patients to the treatment with higher true success rate. But learning from data takes time, and therefore the gains from using such adaptive rules become progressively more evident as the trial size increases. Thus, the expected number of successes can be increased by approximately 10 percent by employing a strong adaptive treatment allocation rule when $N_{max} = 100$, by 15 percent when $N_{max} = 200$, and 20 percent when $N_{max} = 500$.

Another point of interest in the case of $\mathbb{Q}_{alt}$ is the probability of unwanted imbalance, allocating more patients to the inferior control arm than to the better experimental one. The highest risk for this to happen is in the case of *BARTA* design (c), for which it was found to be approximately 5 percent when $N_{max} = 200$. The corresponding percentages for $N_{max} = 100$ and $N_{max} = 500$ were, respectively, 10 and 3. *BARTA* (c) appears to be the only design, among those considered, for which there is a non-negligible probability that the imbalance turns out to be serious. For the other designs, including different versions of Thompson's rule, the probabilities are much smaller, and very small for $N_{max} = 500$.

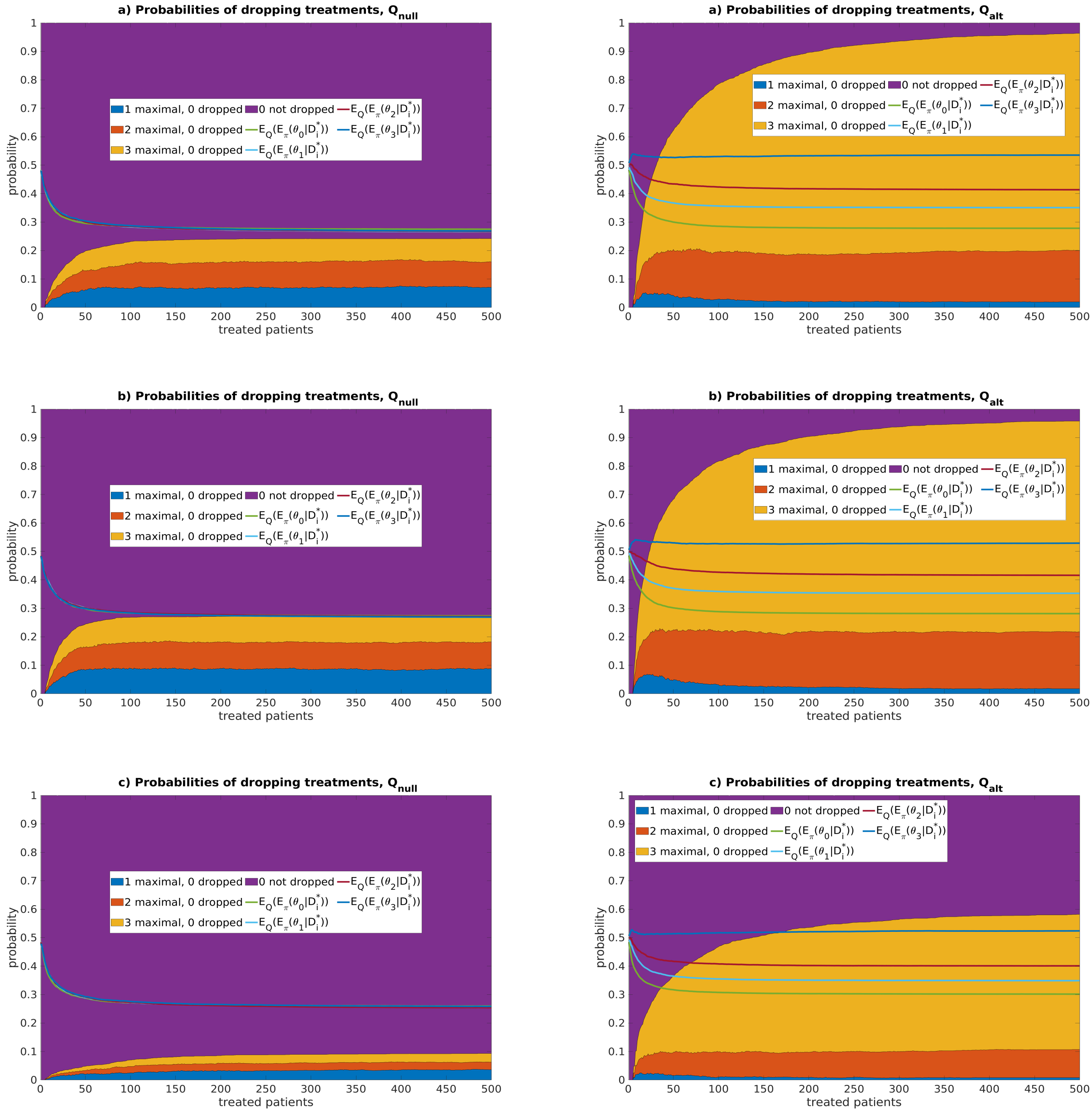

Figure S9: Effect of the design parameters ε and δ in the 4-arm trial of Experiment 2 when applying *BARTS* for treatment selection. Joint probabilities of some combinations of selected treatments are shown, as functions of the number i of treated patients. The results are based on 2000 data sets of size $N_{\max} = 500$, under $\mathbb{Q}_{null}$ (left) and $\mathbb{Q}_{alt}$ (right). Three combinations of design parameters were considered: (a) $\varepsilon = 0.1, \varepsilon_1 = 0, \varepsilon_2 = 0.05$, $\delta = 0.1$ (top), (b) $\varepsilon = 0.05, \varepsilon_1 = 0, \varepsilon_2 = 0.05$, $\delta = 0.1$ (middle), (c) $\varepsilon = 0.2, \varepsilon_1 = 0, \varepsilon_2 = 0.05$, $\delta = 0.05$ (bottom). In the subfigures, the width of each of the 4 bands corresponds to the $\mathbb{Q}$-probability of a respective event in the box. Also shown are the expectations $\mathbb{E}_{\mathbb{Q}_{null}}\big(\mathbb{E}_\pi\big(\boldsymbol{\theta}_k|D_i^*\big)\big)$ and $\mathbb{E}_{\mathbb{Q}_{alt}}\big(\mathbb{E}_\pi\big(\boldsymbol{\theta}_k|D_i^*\big)\big), (1 \le i \le 500, 1 \le k \le 3)$, computed from these simulations. For more details, see text.

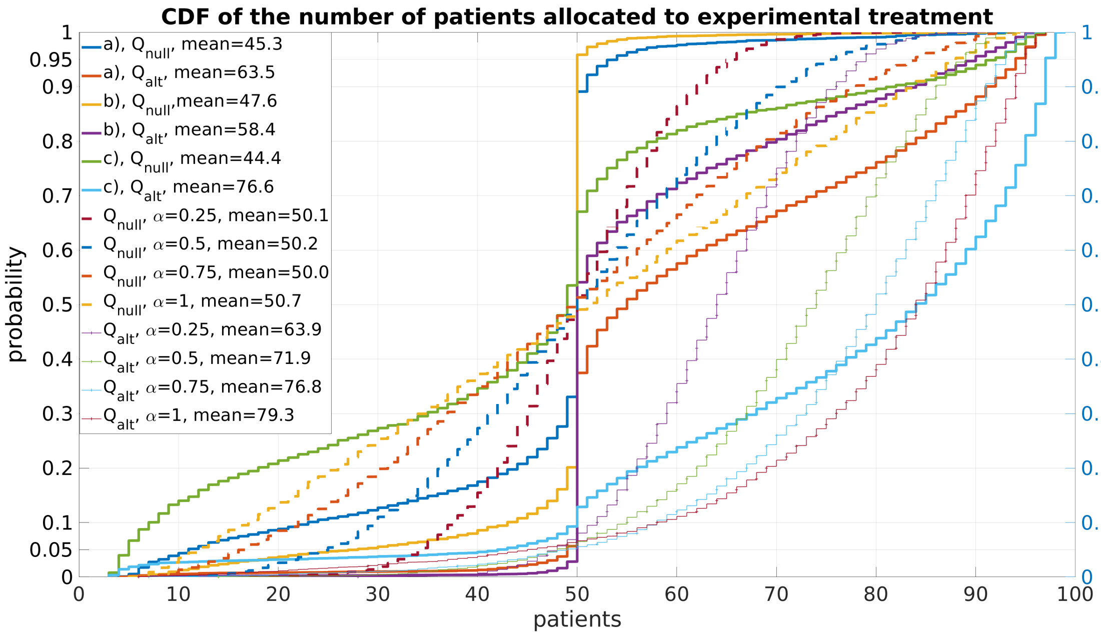

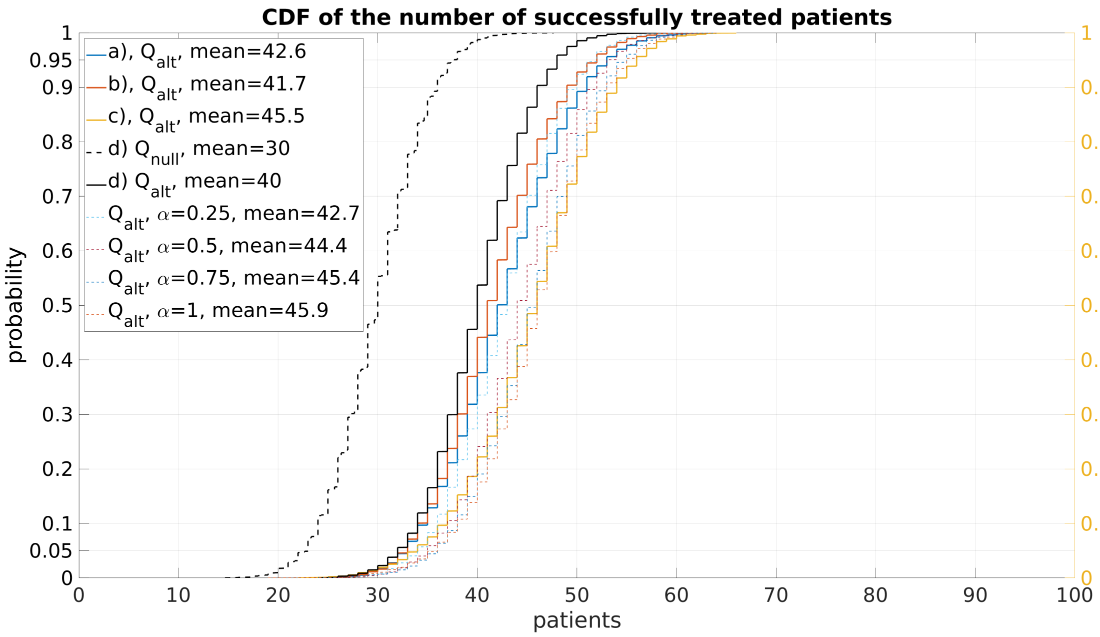

Figure S10: Effect of the choice of the threshold parameters ε and δ in *BARTA* on the number of patients allocated to the experimental treatment and on the total number of treatment successes. Cumulative distribution functions of $N_1(100)$ (top) and $S(100)$ (bottom) are shown, based on 5000 simulated data sets, under $\mathbb{Q}_{null}$ with true parameter values $\theta_0 = \theta_1 = 0.3$ and $\mathbb{Q}_{alt}$ with values $\theta_0 = 0.3, \theta_1 = 0.5$. Three combinations of the design parameters were used: (a) $\varepsilon = 0.1$, $\delta = 0.1$, (b) $\varepsilon = 0.05$, $\delta = 0.1$, (c) $\varepsilon = 0.2$, $\delta = 0.05$. In addition, (d) represents a completely symmetric treatment allocation. For comparison we also plot the corresponding CDF under the alternative hypothesis obtained by using fractional Thompson's rule with respective parameters $\kappa = 0.25, 0.5, 0.75$ and 1.

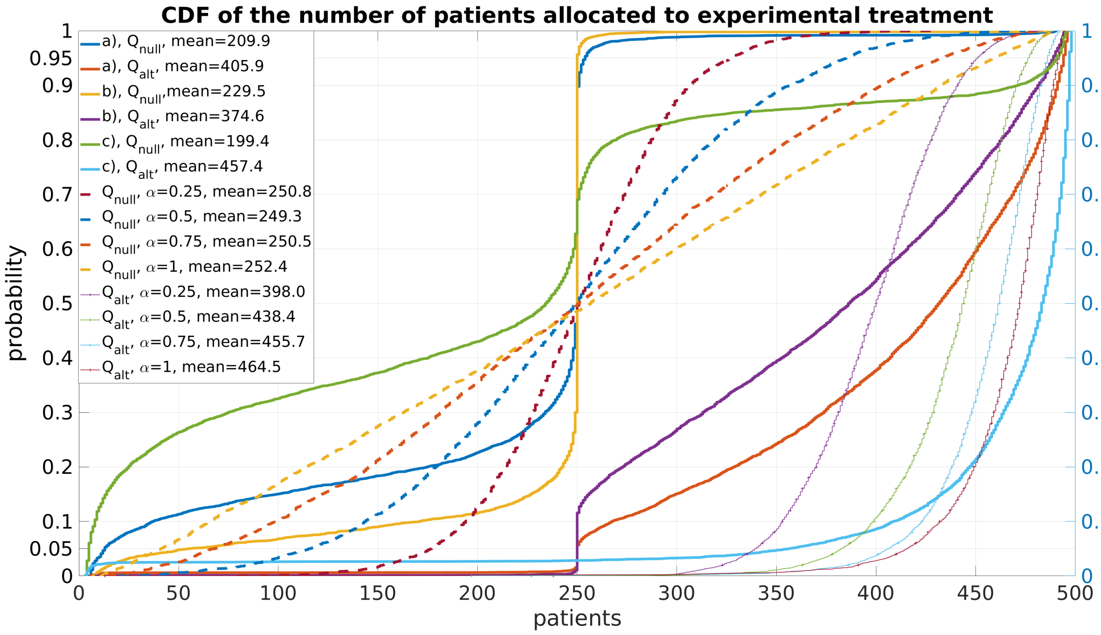

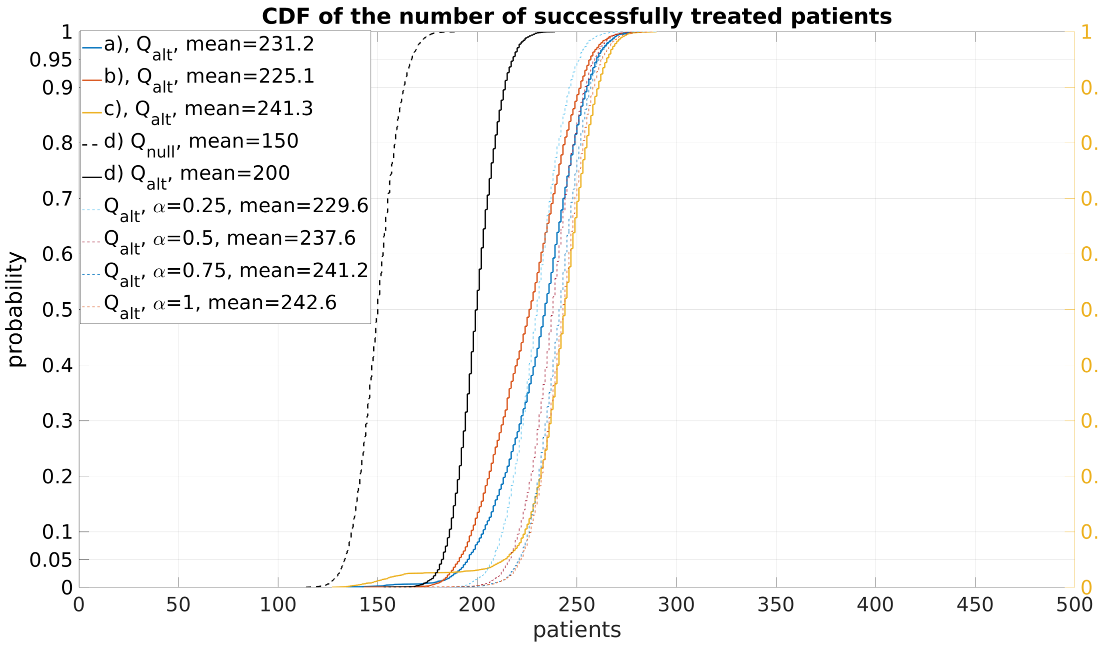

Figure S11: Effect of the choice of the threshold parameters ε and δ in *BARTA* on the number of patients allocated to the experimental treatment and on the total number of treatment successes. Cumulative distribution functions of $N_1(500)$ (top) and $S(500)$ (bottom) are shown, based on 5000 simulated data sets, under $\mathbb{Q}_{null}$ with true parameter values $\theta_0 = \theta_1 = 0.3$ and $\mathbb{Q}_{alt}$ with values $\theta_0 = 0.3, \theta_1 = 0.5$. Three combinations of the design parameters were used: (a) $\varepsilon = 0.1$, $\delta = 0.1$, (b) $\varepsilon = 0.05$, $\delta = 0.1$, (c) $\varepsilon = 0.2$, $\delta = 0.05$. In addition, (d) represents a completely symmetric treatment allocation. For comparison we also plot the corresponding CDF under the alternative hypothesis obtained by using fractional Thompson's rule with respective parameters $\kappa = 0.25, 0.5, 0.75$ and 1.

D Additional figures and tables to subsection B.1.3

Effect of trial size on frequentist performance

In subsection B.1.3 above we studied the performance of different adaptive designs in terms of true and false positive and negative rates, by considering trial size $N_{max} = 200$ in Figure S5 and Table S1. Below we present corresponding results for $N_{max} = 100$ in Figure S12 and Table S2, and for $N_{max} = 500$ in Figure S13 and Table S3. When combined, these results give us an idea about how such measures depend on the size of the trial.

Figures S12 and S13 bear close similarity to Figure S5. The main differences can be seen in the CDFs arising from data generated under $\mathbb{Q}_{alt}$. The CDFs of the posterior probabilities $\mathbb{P}_\pi(\boldsymbol{\theta}_1 \geq \boldsymbol{\theta}_0 | \mathbf{D}^*_{N_{max}})$ move to the right as N_{max} grows from 100 to 200 and then to 500, thereby signalling that these probabilities become stochastically larger with growing trial size. A similar movement, somewhat slower and in the opposite direction, is seen in the CDFs of $\mathbb{P}_\pi(\boldsymbol{\theta}_0 + 0.05 \geq \boldsymbol{\theta}_1 | \mathbf{D}^*_{N_{max}})$ with growing N_{max}.

The following conclusions can now be made from Tables S1, S2 and S3. Under $\mathbb{Q}_{null}$, the false positive rates are generally somewhat smaller for larger trial sizes, but remain under 0.025 even in the case of $N_{max} = 100$. The true negative rates are usually larger, by a few percentage points, when the trial size is changed from 100 to 200 and then to 500, and the inconclusive rates correspondingly smaller, typically attaining values on either side of ninety percent. The false negative rates are very small for all considered designs.

In contrast, as can be expected, the true positive rate (*power*) under $\mathbb{Q}_{alt}$ depends strongly on the size of the trial. As reported in B.1.3, for $N_{max} = 200$ it has the moderate level of approximately seventy percent for *BARTA* designs (a), (b) and (d), and almost as high for Thompson's rule with $\kappa = 0.25$. For these same designs and $N_{max} = 100$, the true positive rates are lower, on both sides of 45 percent, but for $N_{max} = 500$ already in the range of 95 percent. Again, of interest is to note that, in terms of these frequentist measures, three adaptive rules perform as well as the symmetric block randomization design (d). For Thompson's rule, larger values of κ lead to greater instability in the behavior of the adaptive mechanism and consequent weaker frequentist performance. Of all considered alternatives, the smallest true positive rate is obtained for the design (c) of *BARTA*. The false negative rates are very small for all considered designs.

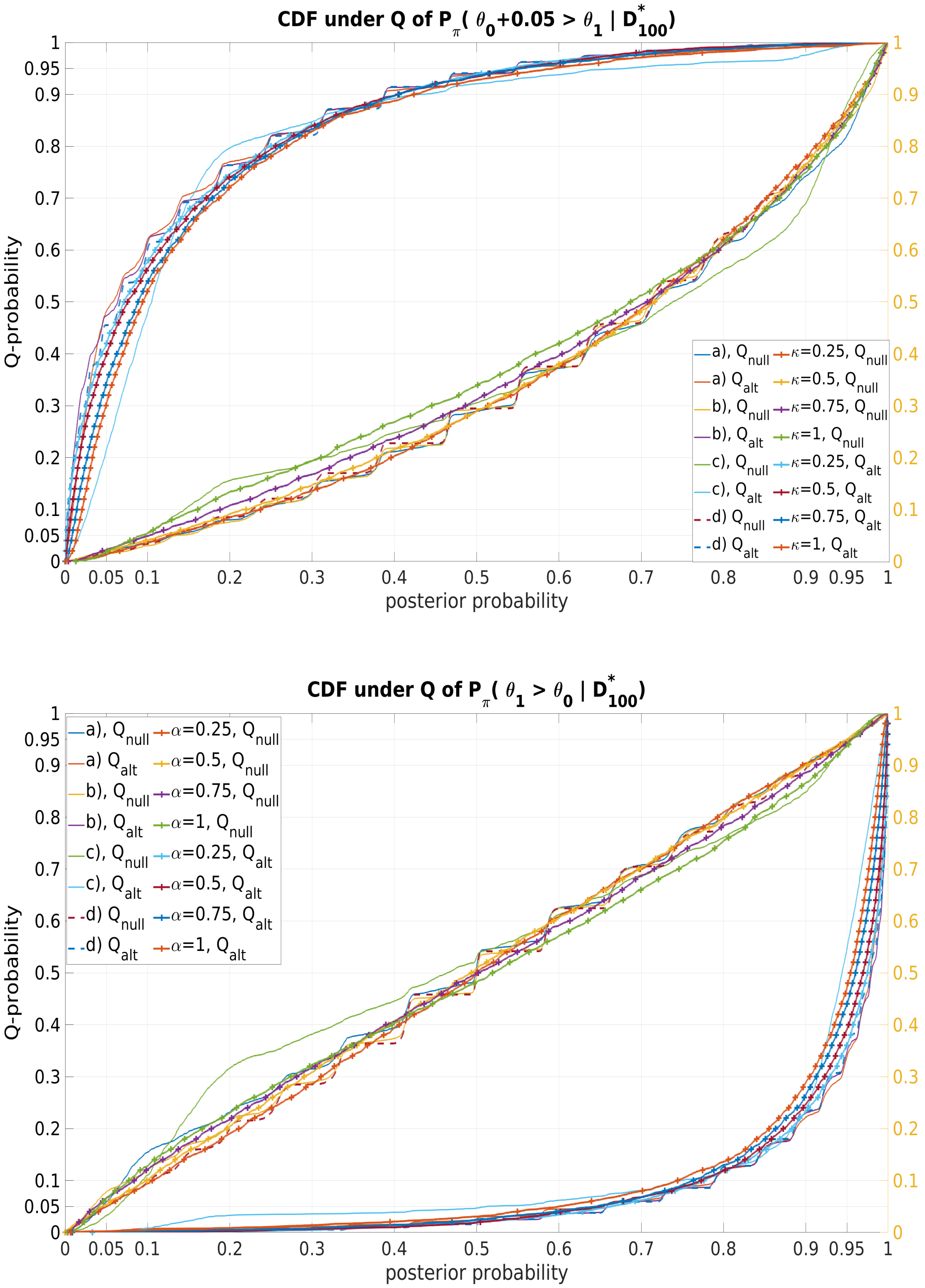

Figure S12: Effect of the design parameters ε and δ of *BARTA*, and κ of Thompson's rule, on the CDFs of the posterior probabilities $\mathbb{P}(\theta_0 + 0.05 \geq \theta_1 | D^*_{100})$ (top) and $\mathbb{P}(\theta_1 \geq \theta_0 | D^*_{100})$ (bottom) in the 2-arm trial of Experiment 1 when applying *BARTA* for treatment allocation and making a final assessment at $i = N_{\max} = 100$. The results are based on 5000 data sets generated under $\mathbb{Q}_{null}$ and $\mathbb{Q}_{alt}$ when using the following combinations of design parameters: (a) $\varepsilon = 0.1, \delta = 0.1$, (b) $\varepsilon = 0.05, \delta = 0.1$, (c) $\varepsilon = 0.2, \delta = 0.05$.

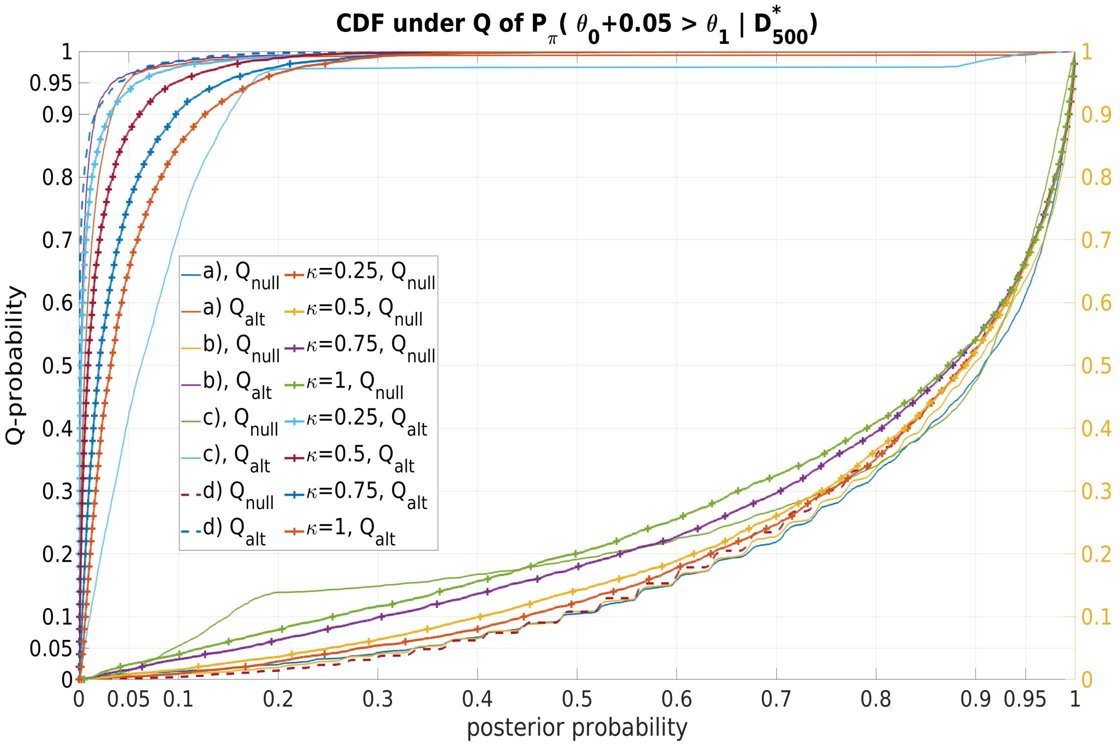

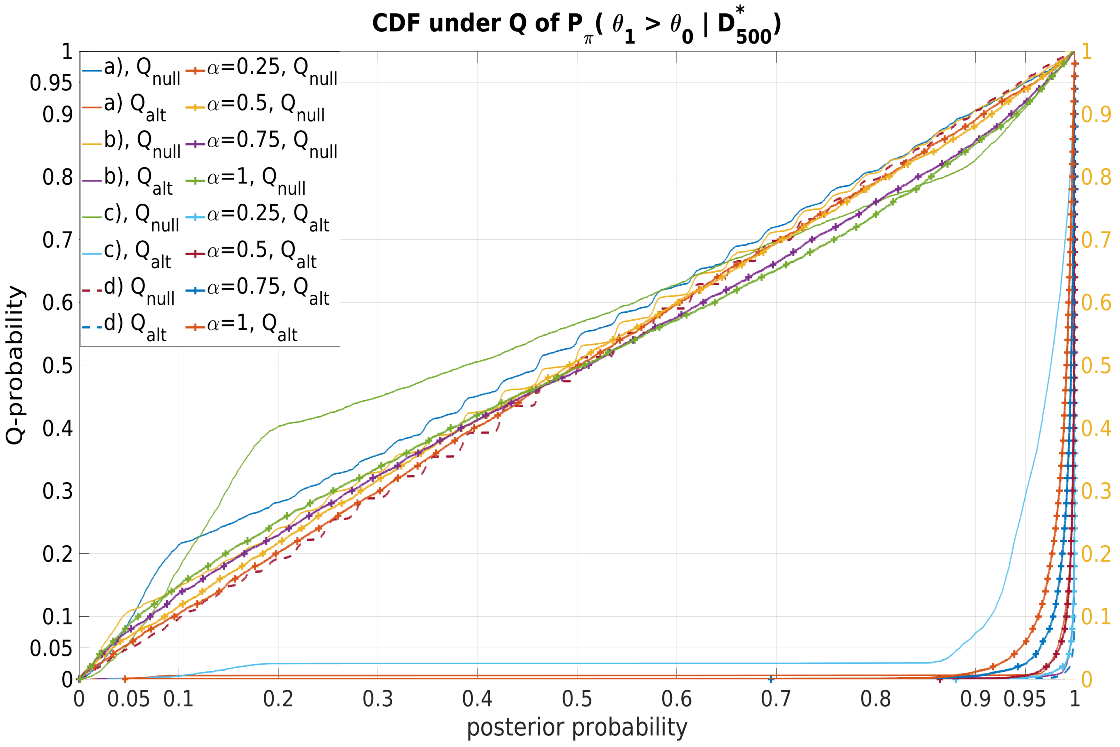

Figure S13: Effect of the design parameters ε and δ of *BARTA*, and κ of Thompson's rule, on the CDFs of the posterior probabilities $\mathbb{P}(\theta_0 + 0.05 \geq \theta_1 | D^*_{500})$ (top) and $\mathbb{P}(\theta_1 \geq \theta_0 | D^*_{500})$ (bottom) in the 2-arm trial of Experiment 1 when applying *BARTA* for treatment allocation and making a final assessment at $i = N_{\max} = 500$. The results are based on 5000 data sets generated under $\mathbb{Q}_{null}$ and $\mathbb{Q}_{alt}$ when using the following combinations of design parameters: (a) $\varepsilon = 0.1, \delta = 0.1$, (b) $\varepsilon = 0.05, \delta = 0.1$, (c) $\varepsilon = 0.2, \delta = 0.05$.

$\varepsilon_0 = 0.05, \delta_0 = 0.05$	(a)	(b)	(c)	(d)	$\kappa = 0.25$	$\kappa = 0.5$	$\kappa = 0.75$	$\kappa = 1$
$\mathbb{Q}_{null}$: false positive	0.020	0.013	0.012	0.016	0.013	0.020	0.022	0.018
$\mathbb{Q}_{null}$: true negative	0.058	0.078	0.032	0.051	0.050	0.054	0.066	0.066
$\mathbb{Q}_{null}$: inconclusive	0.922	0.908	0.956	0.933	0.937	0.926	0.911	0.917
$\mathbb{Q}_{alt}$: true positive	0.482	0.473	0.215	0.455	0.419	0.398	0.344	0.303
$\mathbb{Q}_{alt}$: false negative	0.003	0.001	0.002	~ 0	~ 0	0.001	0.001	0.002
$\mathbb{Q}_{alt}$: inconclusive	0.516	0.525	0.783	0.545	0.581	0.601	0.655	0.695

Table S2: True and false positive and negative rates when applying *BARTA* or Thompson's rule and threshold values $\varepsilon_0 = 0.05$ and $\delta_0 = 0.05$ in a trial of size $N_{max} = 100$.

$\varepsilon_0 = 0.05, \delta_0 = 0.05$	(a)	(b)	(c)	(d)	$\kappa = 0.25$	$\kappa = 0.5$	$\kappa = 0.75$	$\kappa = 1$
$\mathbb{Q}_{null}$: false positive	0.009	0.004	0.014	0.001	0.005	0.008	0.015	0.024
$\mathbb{Q}_{null}$: true negative	0.092	0.108	0.059	0.049	0.057	0.068	0.077	0.086
$\mathbb{Q}_{null}$: inconclusive	0.899	0.888	0.927	0.950	0.939	0.924	0.908	0.890
$\mathbb{Q}_{alt}$: true positive	0.954	0.964	0.421	0.959	0.937	0.873	0.757	0.650
$\mathbb{Q}_{alt}$: false negative	0.002	0.001	0.001	~ 0	~ 0	~ 0	~ 0	~ 0
$\mathbb{Q}_{alt}$: inconclusive	0.044	0.035	0.578	0.041	0.063	0.127	0.243	0.350

Table S3: True and false positive and negative rates when applying *BARTA* or Thompson's rule and threshold values $\varepsilon_0 = 0.05$ and $\delta_0 = 0.05$ in a trial of size $N_{max} = 500$.

Employing an initial burn-in period

In Table S4 we consider the effect of the design modification, where the first 30 patients are divided evenly, by using a block randomization, to the two treatments. Adaptive treatment allocation is then applied after this, either in the form of *BARTA* or Thompson's rule, and the performance measures are evaluated at $N_{max} = 200$ from a simulation experiment of 5000 repetitions. The numerical values in Table S4 are compared naturally to those in Table S1, where the design was the same except that no burn-in was used.

Overall, the differences are small. The largest change is in the values of true positive rate (power) for *BARTA* design (c), which has increased from 0.303 in Table S1 to 0.443 due to the stabilizing initial burn-in. Smaller changes can be seen in the false positive rates for *BARTA* (c) and Thompson's rule with $\kappa = 0.75$ and $\kappa = 1$, where burn-in has trimmed down these already rather low rates by small amounts. The conclusion from this experiment is that, in a trial of size $N_{max} = 200$, employing an initial burn-in period has a small to modest stabilizing effect on the frequentist performance of those adaptive designs in which the adaptive mechanism was strongest.

Remarks on other test variants

In the **first variant**, we consider in Table S5 the case $\delta_0 = 0$, where the special protection against dropping the control arm in the final test at N_{max} has been removed. Thus we write *false positive rate* $= \mathbb{Q}_{null}(\mathbb{P}_\pi(\boldsymbol{\theta}_0 \geq \boldsymbol{\theta}_1 | \mathbf{D}^*_{N_{max}}) \leq \varepsilon_0)$, *true negative rate* $= \mathbb{Q}_{null}(\mathbb{P}_\pi(\boldsymbol{\theta}_1 \geq$

$\varepsilon_0 = 0.05, \delta = 0.05$	(a)	(b)	(c)	(d)	$\kappa = 0.25$	$\kappa = 0.5$	$\kappa = 0.75$	$\kappa = 1$
$\mathbb{Q}_{null}$: false positive	0.014	0.010	0.019	0.008	0.011	0.015	0.015	0.020
$\mathbb{Q}_{null}$: true negative	0.077	0.085	0.061	0.050	0.056	0.057	0.068	0.064
$\mathbb{Q}_{null}$: inconclusive	0.909	0.905	0.920	0.942	0.934	0.928	0.917	0.915
$\mathbb{Q}_{alt}$: true positive	0.727	0.702	0.443	0.689	0.676	0.615	0.533	0.464
$\mathbb{Q}_{alt}$: false negative	~ 0	~ 0	0.001	~ 0	~ 0	~ 0	~ 0	0.001
$\mathbb{Q}_{alt}$: inconclusive	0.272	0.298	0.556	0.311	0.324	0.385	0.467	0.535

Table S4: True and false positive and negative rates when applying *BARTA* or Thompson's rule and threshold values $\varepsilon_0 = 0.05$ and $\delta_0 = 0.05$ and a *burn-in period* of $n_0 = 30$ patients in a trial of size $N_{max} = 200$.

$\varepsilon_0 = 0.05, \delta_0 = 0$	(a)	(b)	(c)	(d)	$\kappa = 0.25$	$\kappa = 0.5$	$\kappa = 0.75$	$\kappa = 1$
$\mathbb{Q}_{null}$: false positive	0.050	0.046	0.082	0.051	0.053	0.056	0.068	0.075
$\mathbb{Q}_{null}$: true negative	0.074	0.086	0.040	0.052	0.054	0.056	0.073	0.074
$\mathbb{Q}_{null}$: inconclusive	0.876	0.868	0.878	0.896	0.892	0.888	0.859	0.851
$\mathbb{Q}_{alt}$: true positive	0.897	0.896	0.622	0.891	0.886	0.857	0.794	0.739
$\mathbb{Q}_{alt}$: false negative	0.002	0.001	0.001	~ 0	~ 0	~ 0	0.001	0.001
$\mathbb{Q}_{alt}$: inconclusive	0.101	0.103	0.377	0.109	0.114	0.143	0.204	0.260

Table S5: True and false positive and negative rates when applying *BARTA* or Thompson's rule and threshold values $\varepsilon_0 = 0.05$ and $\delta_0 = 0$ in a trial of size $N_{max} = 200$. First test variant, see text.

$\boldsymbol{\theta}_0|\mathbf{D}^*_{N_{max}}) \leq \varepsilon_0)$, *true positive rate* $= \mathbb{Q}_{alt}(\mathbb{P}_\pi(\boldsymbol{\theta}_0 \geq \boldsymbol{\theta}_1|\mathbf{D}^*_{N_{max}}) \leq \varepsilon_0)$ and *false negative rate* $= \mathbb{Q}_{alt}(\mathbb{P}_\pi(\boldsymbol{\theta}_1 \geq \boldsymbol{\theta}_0|\mathbf{D}^*_{N_{max}}) \leq \varepsilon_0)$. *Inconclusive rates* are the probabilities $\mathbb{Q}(\mathbb{P}_\pi(\boldsymbol{\theta}_0 \geq \boldsymbol{\theta}_1|\mathbf{D}^*_{N_{max}}) > \varepsilon_0), \mathbb{P}_\pi(\boldsymbol{\theta}_1 \geq \boldsymbol{\theta}_0|\mathbf{D}^*_{N_{max}}) > \varepsilon_0)$, for $\mathbb{Q} = \mathbb{Q}_{null}$ and $\mathbb{Q} = \mathbb{Q}_{alt}$. As noted in the main text, this change from the original criteria implies that, compared to the respective values provided in Table S1, all positive rates are now larger, whereas the negative rates remain intact. Of the former, the rates for *BARTA* (a), (b) and (d), and for Thompson's rule with $\kappa = 0.25$, are again quite similar, with false positive rates varying on both sides of five percent and true positive rates (*power*) reaching levels of almost ninety percent. The frequentist performance of the other designs is somewhat weaker, deteriorating with increasing instability of the allocation rule.

In the **second variant** of the final test, the experimental arm is dropped if $\mathbb{P}_\pi(\boldsymbol{\theta}_1 \geq \boldsymbol{\theta}_0 + \delta_0|\mathbf{D}^*_{N_{max}}) \leq \varepsilon_0$. Therefore, in Table S6 we write *false positive rate* $= \mathbb{Q}_{null}(\mathbb{P}_\pi(\boldsymbol{\theta}_0 + \delta_0 \geq \boldsymbol{\theta}_1|\mathbf{D}^*_{N_{max}}) \leq \varepsilon_0)$, *true negative rate* $= \mathbb{Q}_{null}(\mathbb{P}_\pi(\boldsymbol{\theta}_1 \geq \boldsymbol{\theta}_0 + \delta_0|\mathbf{D}^*_{N_{max}}) \leq \varepsilon_0)$, *true positive rate* $= \mathbb{Q}_{alt}(\mathbb{P}_\pi(\boldsymbol{\theta}_0 + \delta_0 \geq \boldsymbol{\theta}_1|\mathbf{D}^*_{N_{max}}) \leq \varepsilon_0)$ and *false negative rate* $= \mathbb{Q}_{alt}(\mathbb{P}_\pi(\boldsymbol{\theta}_1 \geq \boldsymbol{\theta}_0 + \delta_0|\mathbf{D}^*_{N_{max}}) \leq \varepsilon_0)$. The probabilities $\mathbb{Q}(\mathbb{P}_\pi(\boldsymbol{\theta}_0 + \delta_0 \geq \boldsymbol{\theta}_1|\mathbf{D}^*_{N_{max}}) > \varepsilon_0, \mathbb{P}_\pi(\boldsymbol{\theta}_1 \geq \boldsymbol{\theta}_0 + \delta_0|\mathbf{D}^*_{N_{max}}) > \varepsilon_0)$, for $\mathbb{Q} = \mathbb{Q}_{null}$ and $\mathbb{Q} = \mathbb{Q}_{alt}$, are *inconclusive rates*. This change means that the negative rates, both true and false, are now larger than the respective values in Table S1, while the positive rates remain intact. The true negative rates, which were below ten percent in Table S1, vary in Table S6 on both sides of twenty percent. The inconclusive rates under $\mathbb{Q}_{null}$ are now lower than in Table S5, but still rather high, between seventy-five and eighty percent. The false negative rates are slightly higher than in Table S1, but still very low for all allocation rules. The considered

$\varepsilon_0 = 0.05, \delta_0 = 0.05$	(a)	(b)	(c)	(d)	$\kappa = 0.25$	$\kappa = 0.5$	$\kappa = 0.75$	$\kappa = 1$
$\mathbb{Q}_{null}$: false positive	0.014	0.009	0.014	0.007	0.011	0.014	0.023	0.025
$\mathbb{Q}_{null}$: true negative	0.236	0.216	0.201	0.196	0.187	0.195	0.210	0.197
$\mathbb{Q}_{null}$: inconclusive	0.750	0.776	0.785	0.797	0.803	0.791	0.768	0.778
$\mathbb{Q}_{alt}$: true positive	0.723	0.711	0.303	0.694	0.665	0.598	0.516	0.443
$\mathbb{Q}_{alt}$: false negative	0.005	0.001	0.004	~ 0	~ 0	~ 0	0.001	0.002
$\mathbb{Q}_{alt}$: inconclusive	0.272	0.288	0.693	0.306	0.335	0.402	0.483	0.555

Table S6: True and false positive and negative rates when applying *BARTA* or Thompson's rule and threshold values $\varepsilon_0 = 0.05$ and $\delta_0 = 0.05$ in a trial of size $N_{max} = 200$. Second test variant, see text.

performance measures of *BARTA* (a), (b) and (d), and of Thompson's rule with $\kappa = 0.25$, are again quite similar to each other.

References

1. Lesaffre, E. Superiority, equivalence, and non-inferiority trials. *Bulletin of the NYU hospital for joint diseases* **66** (2008).
2. Meester, S. G. & Mackay, J. A parametric model for cluster correlated categorical data. *Biometrics,* 954–963 (1994).
3. Neuenschwander, B., Capkun-Niggli, G., Branson, M. & Spiegelhalter, D. J. Summarizing historical information on controls in clinical trials. *Clinical Trials* **7.** PMID: 20156954, 5–18. eprint: https://doi.org/10.1177/1740774509356002 (2010).
4. Spiegelhalter, D. J., Abrams, K. R. & Myles, J. P. *Bayesian approaches to clinical trials and health-care evaluation* (John Wiley & Sons, 2004).
5. Spiegelhalter, D. J., Freedman, L. S. & Parmar, M. K. B. Bayesian approaches to randomized trials. *Journal of the Royal Statistical Society: Series A (Statistics in Society)* **157,** 357–387 (1994).
6. Thall, P. & Wathen, J. Practical Bayesian Adaptive Randomization in Clinical Trials. *European journal of cancer (Oxford, England : 1990)* **43,** 859–66 (Apr. 2007).
7. Thall, P. F., Fox, P. S. & Wathen, J. K. Statistical controversies in clinical research: scientific and ethical problems with adaptive randomization in comparative clinical trials. *Annals of oncology : official journal of the European Society for Medical Oncology* **26 8,** 1621–8 (2015).
8. Thall, P. F. & Simon, R. Practical Bayesian Guidelines for Phase IIB Clinical Trials. *Biometrics* **50,** 337 (June 1994).
9. Villar, S. S., Bowden, J. & Wason, J. Multi-armed Bandit Models for the Optimal Design of Clinical Trials: Benefits and Challenges. *Statistical Science* **30,** 199–215 (May 2015).
